# Epigenetic signature predicts overall survival clear cell renal cell carcinoma

**DOI:** 10.1186/s12935-020-01640-x

**Published:** 2020-11-23

**Authors:** Yejinpeng Wang, Liang Chen, Lingao Ju, Kaiyu Qian, Xinghuan Wang, Yu Xiao, Gang Wang

**Affiliations:** 1 Department of Urology, Zhongnan Hospital of Wuhan University, Wuhan, China; 2 Department of Biological Repositories, Zhongnan Hospital of Wuhan University, Wuhan, China; 3 Human Genetics Resource Preservation Center of Hubei Province, Wuhan, China; 4 Human Genetics Resource Preservation Center of Wuhan University, Wuhan, China; 5 Medical Research Institute, Wuhan University, Wuhan, China; 6 Laboratory of Precision Medicine, Zhongnan Hospital of Wuhan University, Wuhan, China

**Keywords:** Epigenetic signature, Clear cell renal cell carcinoma, Overall survival, Methylation, Nomogram

## Abstract

**Background:**

Recently, increasing study have found that DNA methylation plays an important role in tumor, including clear cell renal cell carcinoma (ccRCC).

**Methods:**

We used the DNA methylation dataset of The Cancer Genome Atlas (TCGA) database to construct a 31-CpG-based signature which could accurately predict the overall survival of ccRCC. Meanwhile, we constructed a nomogram to predict the prognosis of patients with ccRCC.

**Result:**

Through LASSO Cox regression analysis, we obtained the 31-CpG-based epigenetic signature which were significantly related to the prognosis of ccRCC. According to the epigenetic signature, patients were divided into two groups with high and low risk, and the predictive value of the epigenetic signature was verified by other two sets. In the training set, hazard ratio (HR) = 13.0, 95% confidence interval (CI) 8.0–21.2, *P* < 0.0001; testing set: HR = 4.1, CI 2.2–7.7, *P* < 0.0001; entire set: HR = 7.2, CI 4.9–10.6, *P* < 0.0001, Moreover, combined with clinical indicators, the prediction of 5-year survival of ccRCC reached an AUC of 0.871.

**Conclusions:**

Our study constructed a 31-CpG-based epigenetic signature that could accurately predicted overall survival of ccRCC and staging progression of ccRCC. At the same time, we constructed a nomogram, which may facilitate the prediction of prognosis for patients with ccRCC.

## Background

Renal cell carcinoma (RCC) is a cancer that originates in the renal epithelial cells and accounts for more than 90% of renal cancer, of which clear cell RCC (ccRCC) is most common subtype and causes the most deaths [1]. According to statistics, ccRCC caused 14,400 deaths in 2017 [2]. The TNM staging system is still the most commonly used clinical tool to stratify ccRCC patients, however, it is not enough to reflect the biological heterogeneity of tumor and accurately predict the prognosis of ccRCC patients [3], and a better marker is urgently needed to help us to accurately predict.

The aberrant methylation status of CpG islands located in the promoter region of tumor genes is becoming increasingly important in the search for new potential biomarkers for cancer [4–6], because these aberrant are relatively stable and potentially reversible [7]. Combining multiple markers, rather than using only one marker to construct a prognostic model, will make the results more stable and reliable, and improve its predictive value [8]. Many studies have used multiple targets to construct the prognostic model of ccRCC [9, 10]. Here, we used the methylation data of the ccRCC of TCGA database to construct a 31-CpG-based signature.

In this study, we used the methylation data of the ccRCC of TCGA database to construct a 31-CpG-based epigenetic signature, which can accurately predict the overall survival rate of ccRCC through the most recently used The Least Absolute Shrinkage And Selection Operator method (LASSO) algorithm. We then verified this epigenetic marker using two validation sets (testing set and entire set). Furthermore, we constructed a nomogram to facilitate clinicians to accurately predict overall survival in ccRCC patients. Our results identified a new 31-CpG-based epigenetic marker that may be a new target for predicting overall survival of ccRCC.

## Methods

### Study population and data collection

**Fig. 1:**
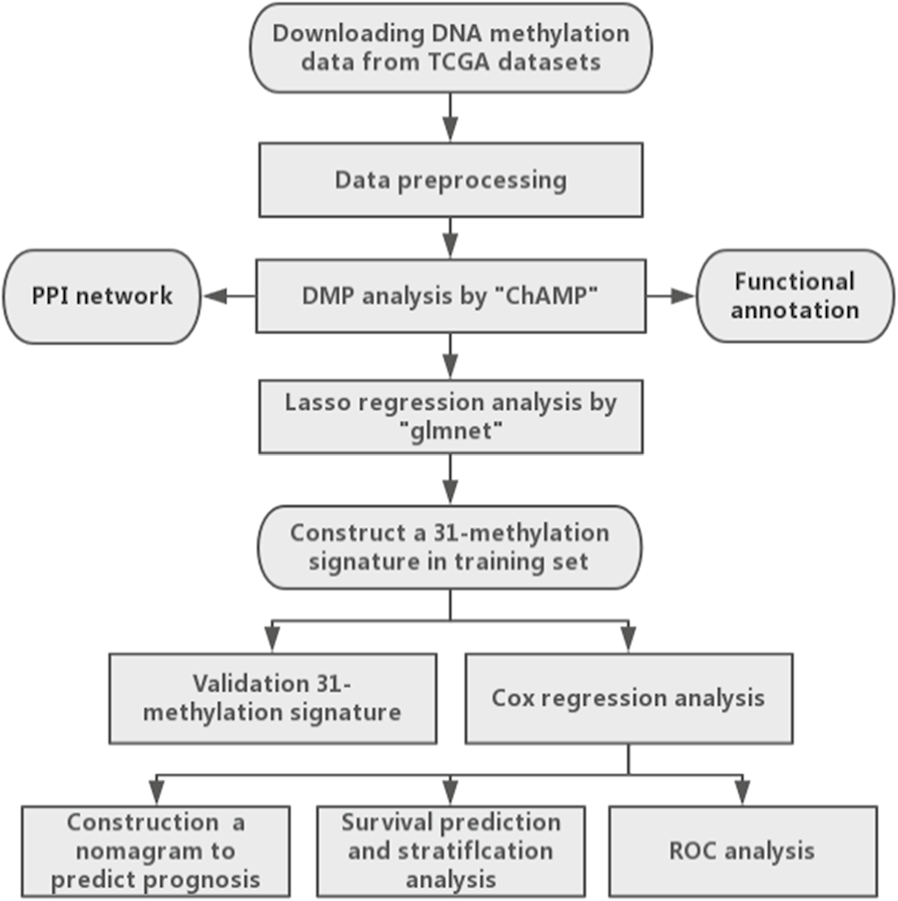
Flow diagram of the study

A total of 319 cases of ccRCC were randomly assigned to two groups, which had a 2:1 ratio. The former was defined as the training set (n = 213) and the latter as testing set (n = 106). The corresponding clinical follow-up information of all cases included the survival time, survival outcome, tumor staging, grading and other information of the cases. The level three of RNA-seq (Illumina RNASeqV2) and the Infinium HumanMethylation450 BeadChip array (Illumina) data were downloaded from The Cancer Genome Atlas (TCGA) database (http://cancergenome.nih.gov/), The details were listed in Table 1, and the flow chart of our entire experiment was shown in Fig. 1.

**Table 1:** Clinical characteristics of ccRCC patients

Variable	Training set	Testing set	Entire set	Chi-square Test	
	n = 213	n = 106	n = 319	χ^2^	*P*
	No. (%)	No. (%)	No. (%)		
Censor	65 (30.5)	40 (37.7)	105 (32.9)		
Age (years)					
≥ 65	75 (35.2)	36 (34.0)	111 (34.8)	0.049	0.825
< 65	138 (64.8)	70 (66.0)	208 (65.2)		
Gender					
Female	80 (37.6)	34 (32.1)	114 (35.7)	0.927	0.336
Male	133 (62.4)	72 (67.9)	205 (64.3)		
Pathological stage					
Stage I	106 (49.8)	49 (46.2)	155 (48.6)	1.766	0.779
Stage II	22 (10.3)	9 (8.5)	31 (9.7)		
Stage III	49 (23.0)	24 (22.6)	73 (22.9)		
Stage IV	35 (16.4)	23 (21.7)	58 (18.2)		
NA	1 (0.5)	1 (0.9)	2 (0.6)		
Histologic grade					
Grade I	6 (2.8)	3 (2.8)	9 (2.8)	4.684	0.321
Grade II	93 (43.7)	40 (30.7)	133 (41.7)		
Grade III	75 (35.2)	48 (45.3)	123 (38.6)		
Grade IV	35 (16.4)	15 (14.2)	50 (15.7)		
NA	4 (1.9)	0 (0.0)	4 (1.3)		
Lymph node metastasis					
Positive	38 (17.8)	23 (21.7)	61 (19.1)	0.681	0.409
Negative	175 (82.2)	83 (78.3)	258 (80.9)		

Here Chi-square and p-value were the values obtained by comparison between the training set and the testing set

### Differentially methylated positions screening

We first filtered out the probes that have too many zeros, then used the R package “ChAMP” [11] to quality control, remove the batch effects, BMIQ normalization [12] and Single Nucleotide Polymorphism (SNP) filtering (if it located in the CpG dinucleotide) [13]. After that, we used “champ.DMP” function to perform differentially methylated positions (DMP) analysis, we chosen the false discovery rate (FDR) < 0.05 and | Δ*β* | ≥ 0.2 as the threshold for screening DMPs.

### Establishment and validation of epigenetic signature

LASSO cox regression analysis was conducted for the DMPs obtained in the previous step, based on R packet “glmnet”. The degree of LASSO regression complexity adjustment was controlled by the parameter *λ*, and the larger *λ* was, the greater the penalty was, so as to obtain a model with fewer variables [14]. We construct the model according to the protocol of official website (https://cran.r-project.org/web/packages/glmnet/glmnet.pdf). Frist, the function “glmnet” returned a sequence of models for us to choose from. Second, we used the function “cv.glmnet” to perform Cross-validation, we followed the protocol, did 100 times cross-validations, and finally got an *λ* average. Based on this *λ* value, we constructed an epigenetic signature based on 31 CpGs from training set. The accuracy of the epigenetic signature was then verified in the testing set and the entire set by using the time-dependent ROC curve and Kaplan–Meier survival curve analysis. Time-dependent ROC curve analysis was performed by R package “survivalROC” [15] while the Kaplan–Meier survival curve analysis was performed by R package “survival”.

### Cox regression analysis of the epigenetic signature

To investigate whether the epigenetic signature we constructed could be independent of other factors affecting the prognosis of ccRCC, we conducted univariate and multivariate analyses by R package “survival”. First of all, the epigenetic signature score, age, gender, neoadjuvant treatment, lymph node count, histologic stage and pathologic grade were performed the univariate analysis. Then, we put the univariate analysis has significant difference factor to carry on the multivariate analysis. Finally, we used the forest map to visualize our results by R package “forestplot”.

### The construction of the nomogram

Based on the results of the previous step, we used the factors with significant differences (epigenetic signature score and histologic stage) in multivariate regression analysis to construct a nomogram by using the R package “rms”. In order to verify the accuracy of the nomogram, we used the calibration curve to evaluate the nomogram. In the calibration curve, if the observed value and the actual value are more coincident, it indicates that the prediction accuracy of the nomogram is higher.

### Functional annotation of the epigenetic signature

We calculated the top 500 genes with the highest correlation with epigenetic signature score, and then enriched the functions of these 500 genes, thus indirectly predicting the functional annotation of epigenetic marker score. The 500 genes were uploaded to the DAVID website (https://david.ncifcrf.gov/) for GO analysis and KEGG pathway analysis. The following, the STRING database (https://string-db.org/) was used to performed protein–protein interaction (PPI) network [16] analysis. We’ve picked the top 50 genes from the “cytoHubba” app at Cytoscape and used the Cytoscape to visualize the results. According to the scores of the epigenetic signature, patients were divided into two groups with high and low risk, and then the corresponding RNA-seq data of these two groups were analyzed by single-samples gene-set enrichment analysis. The R package “GSVA” [17] were used to perform ssGSEA for the 31-CpGs-based epigenetic signature. The C2 (c2.cp.kegg.v6.1.symbols.gmt) set was downloaded from the Molecular Signatures Database (http://software.broadinstitute.org/gsea/msigdb/index.jsp). And it was chosen as the signature to perform ssGSEA. The R package “limma” was used to identify the differentially expressed gene sets. The threshold for screening differentially expressed gene sets were the p-value < 0.05.

## Results

### Screening of DMPs

The R package “ChAMP” was used to screen DMPs between ccRCC and normal ccRCC samples in ccRCC dataset of the TCGA database, a total of 3858 DMPs were identified (1641 up-regulated and 576 down-regulated), under the threshold of FDR < 0.05 and | Δ*β* | ≥ 0.2. All DMPs were listed in Additional file 1: Table S1.

### Establishment and validation of the epigenetic signature

**Fig. 2:**
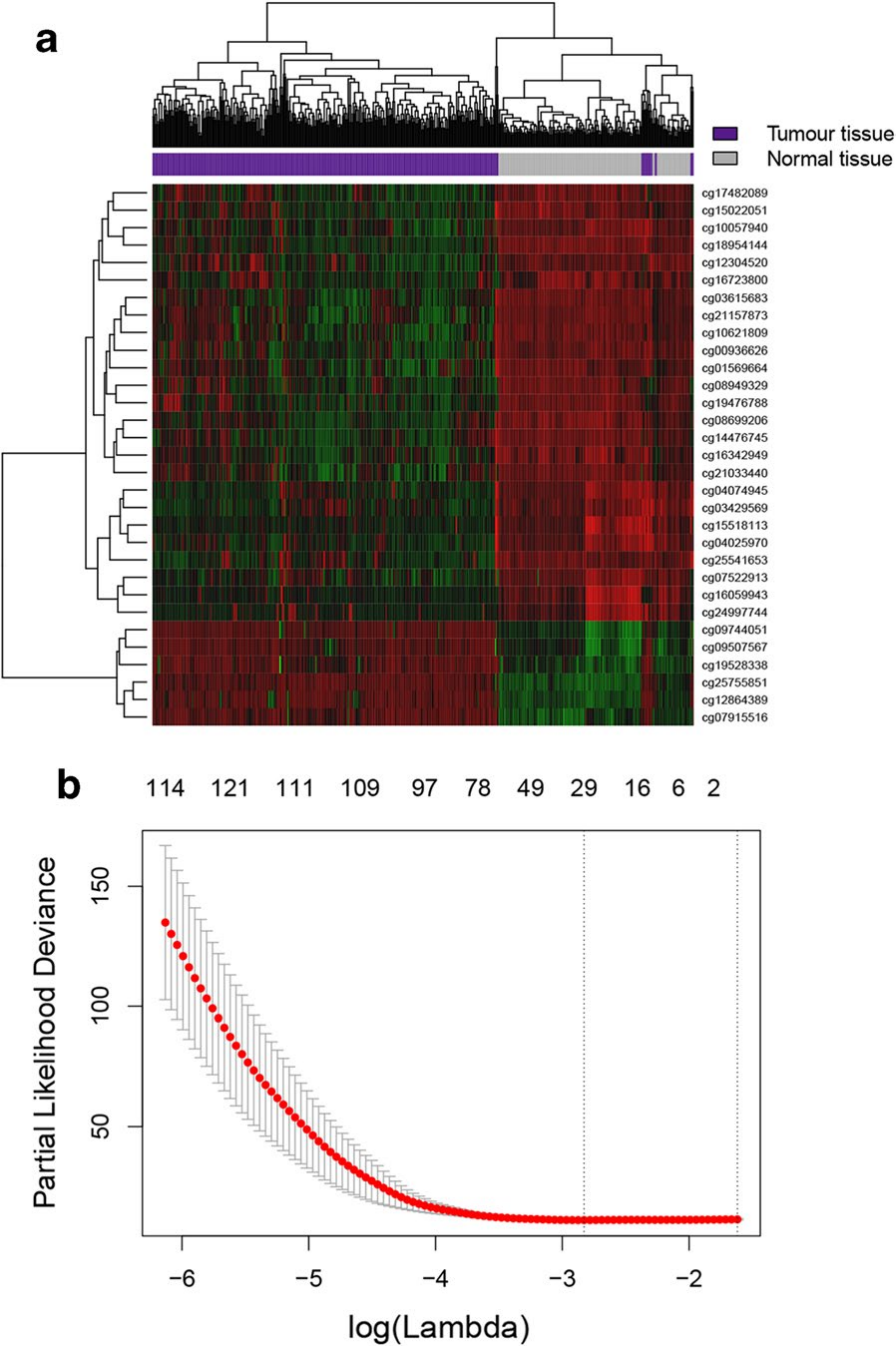
Establishment of epigenetic signature based on 31-CpG-sites. **a** Heatmap of the 31-CpG-sites that were used to construct the epigenetic signature. **b** Ten-time cross-validation for parameter chosen. Here, λ = 0.054 was chosen by ten-time cross-validation

We used the R package of “glmnet” to construct LASSO cox regression model. After 100 cross - validation, we got an optimal *λ* value (*λ* = 0.054), and finally we constructed an epigenetic signature based on 31 CpGs. The formula of epigenetic signature score were obtained by calculating the LASSO regression coefficient: epigenetic signature score = $\sum\nolimits_{k = 1}^{n} {\beta_{ki} X_{ki} }$, here, *n* is the number of the CpG site; *β* is the LASSO regression coefficient of the CpG site; *X* is the methylation value of CpG *k* and patient *i*; *k* is the CpG site. Details of the 31 CpGs were listed in Table 2, the heatmap of the 31 CpGs were shown in Fig. 2a, and the results of ten-time cross-validation were shown in Fig. 2b.

**Table 2:** 31-CpG-sites details in the epigenetic signature

CpG ID	Gene symbol	CHR	Strand	Type	Feature	Delta β	Lasso coef
cg00936626	PIGZ	3	F	II	5′UTR	− 0.20	0.96
cg01569664	IRS2	13	R	I	Body	− 0.20	0.2
cg03429569		2	R	II	IGR	− 0.22	− 0.31
cg03615683		12	R	II	IGR	− 0.21	0.62
cg04025970	MFHAS1	8	R	II	Body	− 0.23	0.12
cg04074945	PHF21A	11	F	II	Body	− 0.26	− 0.06
cg07522913	HOXA3	7	F	I	5′UTR	− 0.27	− 0.25
cg07915516	AXIN1	16	R	II	Body	0.22	0.67
cg08699206		4	R	I	IGR	− 0.34	0.93
cg08949329	COL4A2	13	R	II	Body	− 0.22	0.6
cg09507567		10	R	II	IGR	0.24	0.06
cg09744051	A4GALT	22	F	II	5′UTR	0.25	0.59
cg10057940		10	F	II	IGR	− 0.24	0.57
cg10621809		12	R	II	IGR	− 0.24	0.47
cg12304520	PCDHGA4	5	R	II	Body	− 0.22	0.7
cg12864389	PLEC1	8	F	II	Body	0.37	− 1.15
cg14476745	LHX6	9	R	I	Body	− 0.31	0.1
cg15022051		6	R	I	IGR	− 0.25	0.48
cg15518113	CD247	1	R	II	3′UTR	− 0.22	1.09
cg16059943	PRKCZ	1	F	II	Body	− 0.23	− 1.38
cg16342949	MACROD1	11	F	I	Body	− 0.24	0.38
cg16723800	TAF7L	X	F	I	Body	− 0.31	0.04
cg17482089	PCDHA6	5	R	I	Body	− 0.24	0.73
cg18954144		2	R	I	IGR	− 0.30	0.43
cg19476788	NFATC1	18	R	I	Body	− 0.26	0.08
cg19528338	BMP2	20	R	II	5′UTR	0.21	0.6
cg21033440	SIPA1	11	F	I	Body	− 0.26	0.65
cg21157873	CGN	1	R	II	5′UTR	− 0.26	0.03
cg24997744	BAHCC1	17	R	I	3′UTR	− 0.22	− 1.27
cg25541653	MPPED2	11	R	II	Body	− 0.25	− 1.29
cg25755851		1	F	II	IGR	0.41	− 0.13

**Fig. 3:**
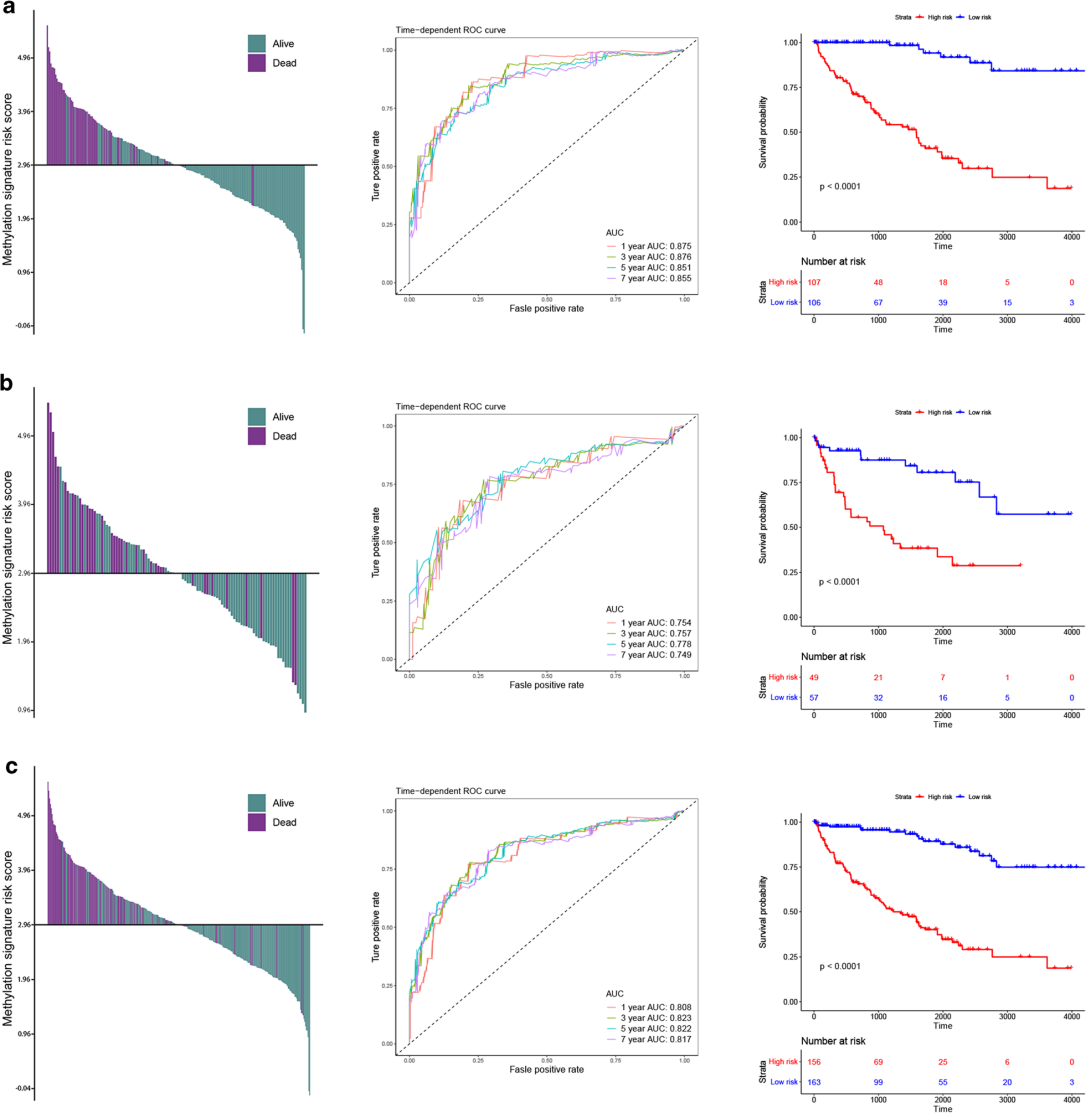
Risk scores by the epigenetic signature, the time-dependent ROC curves and Kaplan–Meier survival curves in the training set, testing set and entire set. **a** Training set. **b** Testing set. **c** Entire set. The AUCs at 1, 3, 5, and 7 years were used to assess the prognostic accuracy, and the log-rank test was used to calculated the p-values

We divided the patients in the training set into high and low risk groups based on the median epigenetic signature scores (here, the median epigenetic signature score was 2.96, Fig. 3a, left panel). Then, time-dependent ROC curve analysis revealed the epigenetic signature we constructed were able to predict the overall survival rate of patients with ccRCC with great accuracy. The AUC of time-dependent ROC for 1-year overall survival was 0.875, 3-year 0.876, 5-year 0.851 and 7-year 0.855 (Fig. 3b middle panel). Finally, Kaplan–Meier survival curve analysis showed that the high-risk group had a significantly worse prognosis than the low-risk group (HR = 13.0, 95% CI 8.0–21.2, *P* < 0.0001, Fig. 3a right panel). In order to avoid over-fitting effect, testing set (HR = 4.1, CI 2.2–7.7, *P* < 0.0001, Fig. 3b) and entire set (HR = 7.2, CI 4.9–10.6, *P* < 0.0001, Fig. 3c) were used for verification our results.

### Univariate and multivariate regression analysis of the epigenetic signature

**Fig. 4:**
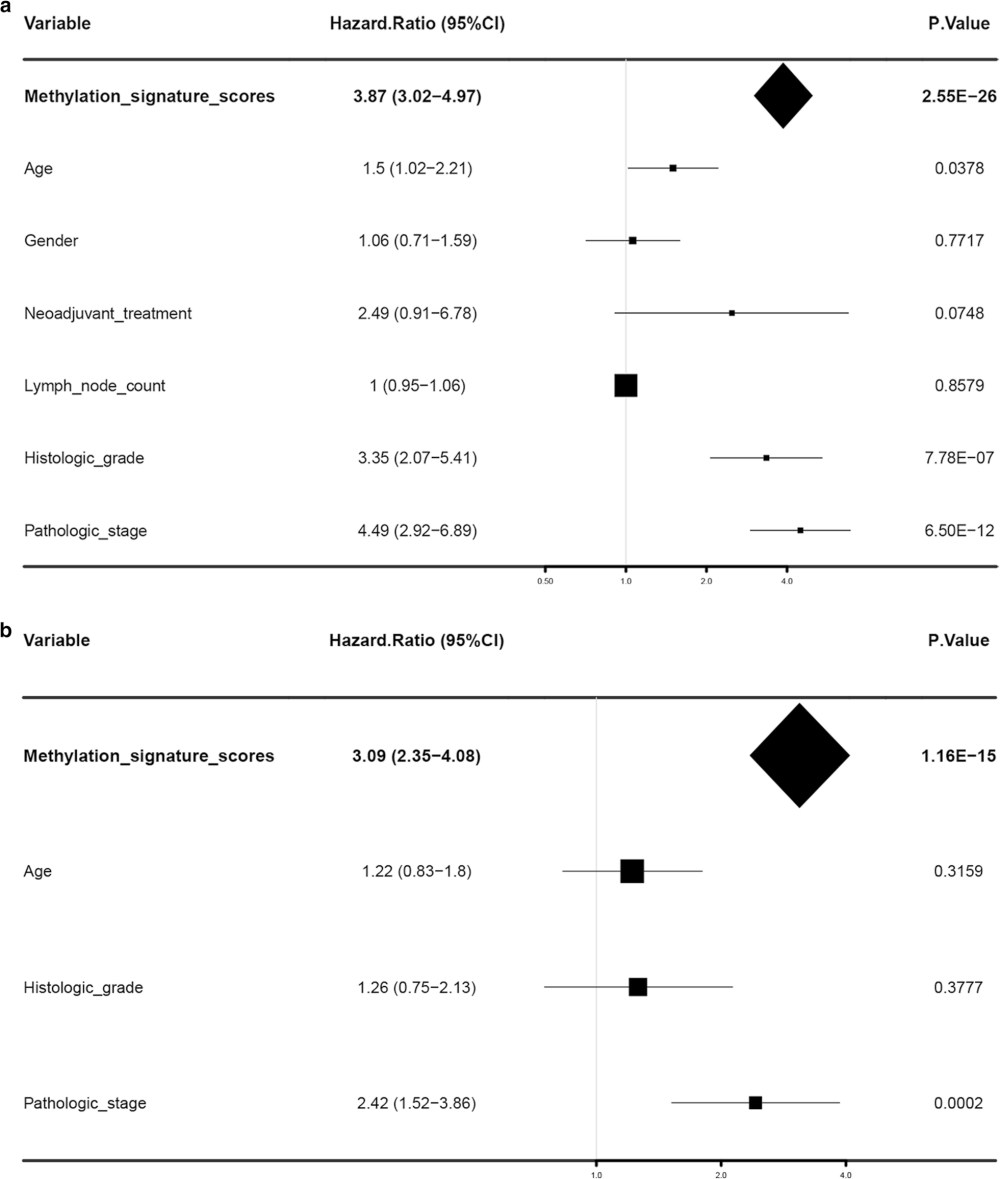
Forest plot of univariable and multivariable analysis. Univariable analysis (**a**) and multivariable analysis (**b**) of the methylation signature scores, age, gender, histologic grade, and so on

We first performed a univariate analysis of the epigenetic signature scores, age, gender, neoadjuvant treatment, lymph node examination, histologic grade and pathologic stage in the entire set, and the results revealed that only the epigenetic signature score (HR = 3.87, 95% CI 3.02–4.97, *P* < 0.0001), age (HR = 1.5, 95% CI 1.02–2.21, *P* = 0.0378), histologic grade (HR = 3.35, 95% CI 2.07–5.41, *P* < 0.0001) and pathologic stage (HR = 4.49, 95% CI 2.92–6.89, *P* < 0.0001) have significant prognostic value (Fig. 4a). Next, we conducted multivariate analysis of the factors with significant differences above, and found that the epigenetic signature score (HR = 3.09, 95% CI 2.35–4.08, *P* < 0.0001) and pathologic stage (HR = 2.42, 95% CI 1.52–3.86, *P* = 0.0002) could accurately predict the overall survival rate of ccRCC independently of other factors (Fig. 4b, Table 3).

**Table 3:** Univariate analysis and multivariate analysis of the epigenetic signature

Variable	Univariate analysis			Multivariate analysis		
	HR	95% CI	*P*	HR	95% CI	*P*
MS scores	3.87	(3.02–4.97)	< 0.001	3.09	(2.35–4.08)	< 0.001
Age	1.5	(1.02–2.21)	0.038	1.22	(0.83–1.8)	0.316
Gender	1.06	(0.71–1.59)	0.772			
Neoadjuvant treatment	2.49	(0.91–6.78)	0.075			
Lymph node count	1	(0.95–1.06)	0.858			
Histologic grade	3.35	(2.07–5.41)	< 0.001	1.26	(0.75–2.13)	0.378
Pathologic stage	4.49	(2.92–6.89)	< 0.001	2.42	(1.52–3.86)	< 0.001

### Nomogram creating and calibrating

**Fig. 5:**
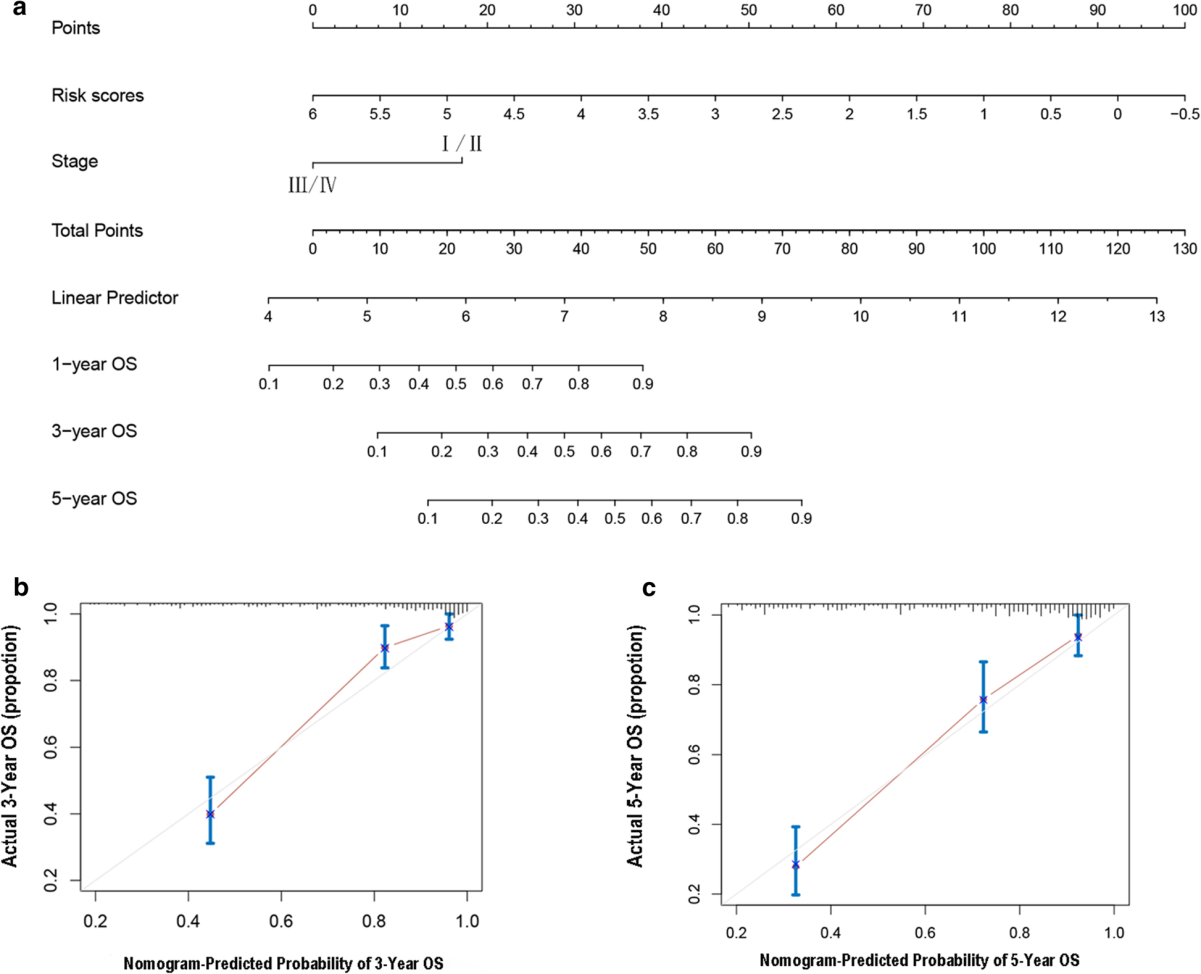
The nomogram to predict the overall survival of ccRCC. **a** The nomograms for predicting proportion of ccRCC patients with 1-, 3- or 5-year overall survival. Plots depict the calibration of the nomogram between predicted and observed 3- (**b**) or 5- (**c**) year outcomes

In order to more conveniently predict the overall survival rate of ccRCC patients for clinicians, we constructed a nomogram based on the R package “rms”. This nomogram could accurately predict 3- or 5-year overall survival in patients with ccRCC (Fig. 5a). Moreover, we used the calibration curve to calibrate the nomogram and found that the prediction value of nomogram was significantly correlated with the actual value (Fig. 5b, c).

### Subgroup analysis of the epigenetic signature

**Fig. 6:**
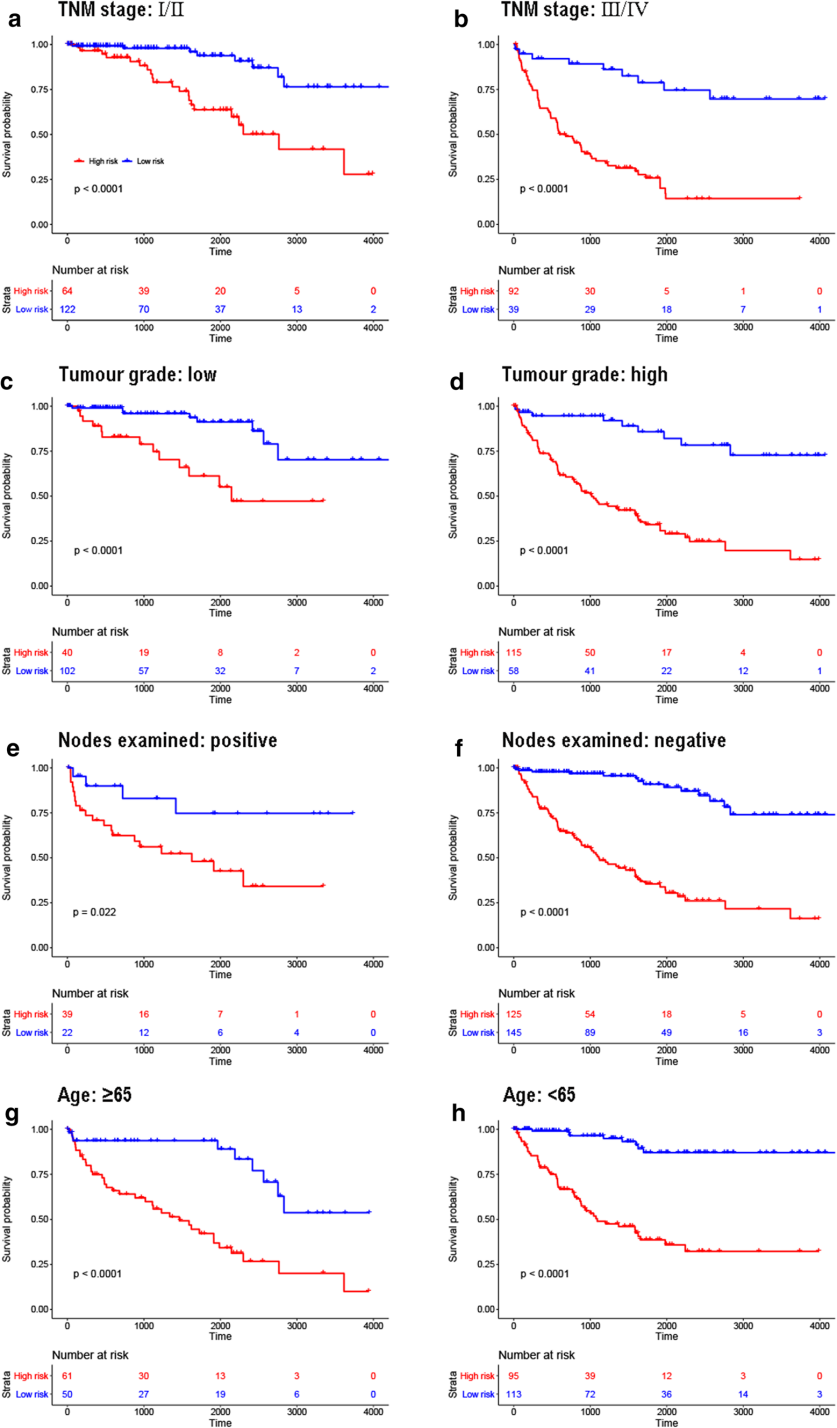
Kaplan–Meier survival analysis for entire set according to the 31-CpG-sites based epigenetic signature stratified by clinicopathological risk factors. **a**, **b** Pathologic stage. **c**, **d** Histologic grade. **e**, **f** Results of lymph nodes examined. **g**, **h** Age

To explore the predictive value of epigenetic signature in different subgroups, we performed subgroup analyzed patients from the entire set according to different characteristics: pathologic stage (III/IV vs I/II, Fig. 6a, b), histologic grade (III/IV vs I/II, Fig. 6c, d), lymph node examination (Positive vs negative, Fig. 6e, f) and age (>=65 vs < 65, Fig. 6g, h). The detailed information of subgroup analysis for the epigenetic signature were listed in Table 4.

**Table 4:** Kaplan–Meier survival analysis for the epigenetic signature in these three sets

Variable	Training set (n = 213)		Testing set (n = 106)		Entire set (n = 319)	
	HR (95% CI)	*P* value	HR (95% CI)	*P* value	HR (95% CI)	*P* value
All	13.0 (8.0–21.2)	< 0.0001	4.1 (2.2–7.7)	< 0.0001	7.2 (4.9–10.6)	< 0.0001
Age (years)						
≥ 65	7.6 (3.7–15.8)	< 0.0001	2.9 (1.2–7.4)	0.0178	4.5 (2.5–8.0)	< 0.0001
< 65	16.8 (8.6–32.8)	< 0.0001	5.4 (2.3–12.7)	0.0002	9.6 (5.7–16.3)	< 0.0001
Gender						
Female	15.7 (6.8–36.4)	< 0.0001	4.5 (1.2–16.6)	0.0071	9.3 (4.6–18.8)	< 0.0001
Male	12.1 (6.5–22.5)	< 0.0001	4.0 (1.9–8.2)	0.0005	6.4 (4.0–11.2)	< 0.0001
Pathological stage						
Stage I/II	10.4 (4.3–25.3)	< 0.0001	–	0.4414	5.2 (2.4–11.4)	< 0.0001
Stage III/IV	10.7 (5.9–19.3)	< 0.0001	2.5 (1.2–5.2)	0.0258	5.3 (3.3–8.3)	< 0.0001
Histologic grade						
Grade I/II	10.6 (3.0–38.1)	< 0.0001	–	0.5311	5.1 (1.9–13.8)	< 0.0001
Grade III/IV	10.1 (5.8–17.8)	< 0.0001	3.9 (2.0–7.9)	0.0007	6.1 (3.9–6.4)	< 0.0001
Lymph node metastasis						
Positive	–	0.0048	–	0.5651	3.3 (1.4–7.4)	0.0222
Negative	12.2 (7.1–21.0)	< 0.0001	5.5 (2.5–12.3)	< 0.0001	8.6 (5.5–13.5)	< 0.0001

### Functional annotation of the epigenetic signature

Pearson correlation coefficients of all genes and the epigenetic signature scores were calculated. The 500 genes with the highest correlation were selected for subsequent analysis, and the threshold value was set as p-value less than 0.05. Functional enrichment of the 500 genes showed that this epigenetic signature was mainly enriched in the cell adhesion, notch signaling pathway, axon guidance, thyroid hormone signaling pathway and so on (Additional file 2: Figure S1). The PPI network diagram we built was shown in Additional file 3: Figure S2.

**Fig. 7:**
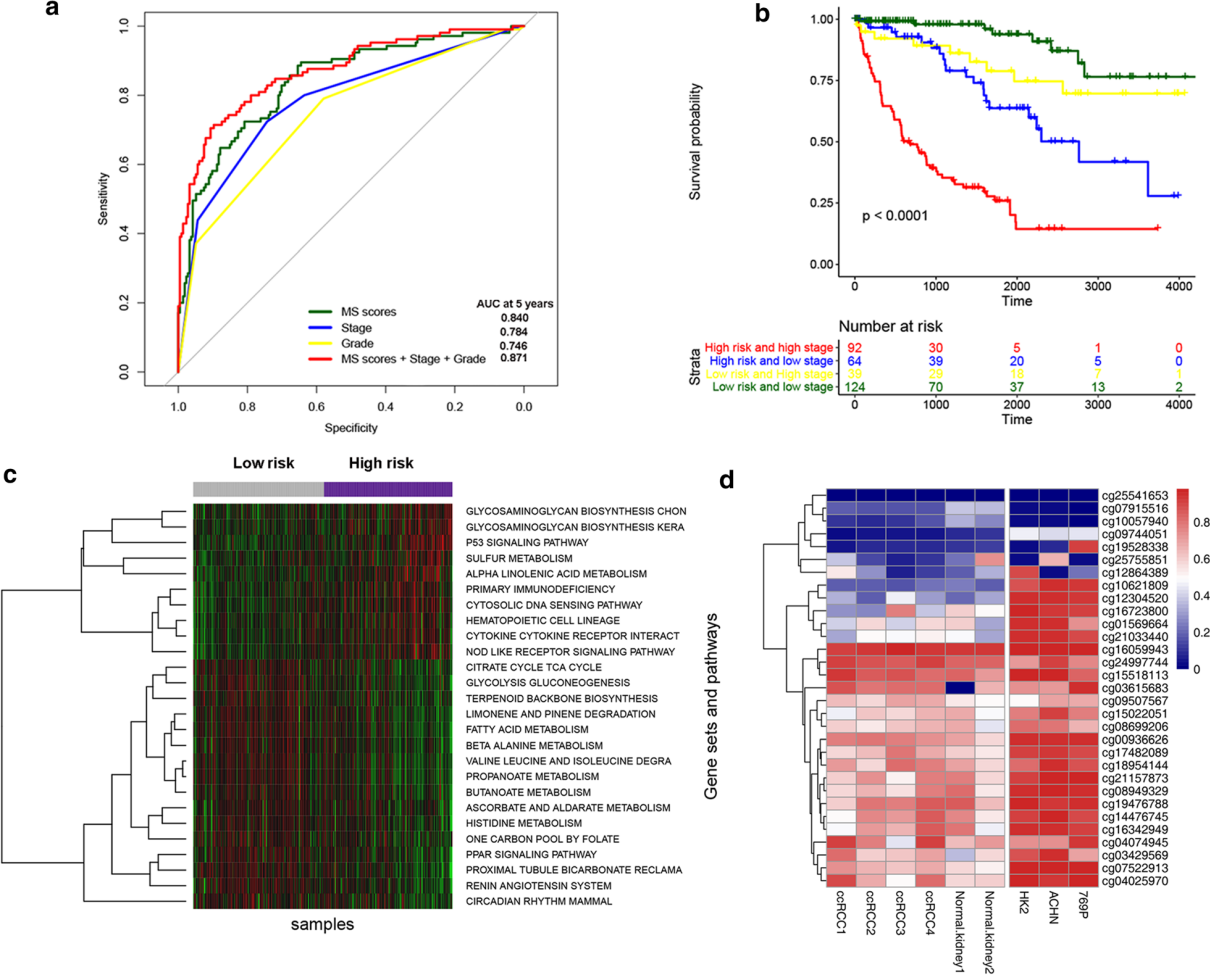
ROC analysis and Subgroup analysis of the 31-CpG-based epigenetic signature. **a** Comparisons of the prognostic accuracy by the epigenetic signature (high risk vs low risk), histologic grade (III/IV vs I/II), pathologic stage (III/IV vs I/II) or the epigenetic signature and clinicopathological prognostic factor combined. **b** Subgroup analysis with pathologic stage of entire set. **c** The heatmap of the results of ssGSEA. The columns (represents ccRCC patients) were ordered from low to high based on epigenetic signature scores. **d** The heatmap of the CpG islands methylation level in the different tissues, cell lines

After the enrichment scores of each sample were calculated, the differences of the high and low groups were analyzed according to the epigenetic signature scores. A total of 26 differentially expressed gene sets (10 up-regulated and 16 down-regulated) were screened under the threshold FDR < 0.05 and |LogFC| > 0.015. The result of ssGSEA revealed that patients in high risk group mainly enriched in p53, nod like receptor, cytosolic DNA sensing and other signaling pathways. The patients in low risk group were mainly enriched in the fatty acid metabolism pathway, PPAR signaling pathway, renin angiotensin system and so on (Fig. 7c, Additional file 1: Table S2).

### Verification of CpG island methylation levels in ccRCC tissues and renal carcinoma cell lines

We collected 4 samples diagnosed as ccRCC and 2 paracancer tissue from the Zhongnan Hospital of Wuhan University, plus human renal tubular epithelial cell line (HK2) and 2 kidney cancer cell lines (ACHN and 769-P), and sequenced them with targeted methylation (MethylTarget, Illumina Hiseq, Illumina, CA, USA). The results showed that CpG island involved in the construction of prognostic model had different expression patterns between carcinoma and paracancer (Fig. 7d).

## Discussion

Patients with ccRCC exhibit a highly invasive clinical process, poor prognosis, and high recurrence rate [1]. The TNM staging system is still widely used to predict the prognosis of ccRCC, however, it relies mainly on anatomical information and has no biological characteristics [9]. Therefore, we need a new target to accurately predict the prognosis of ccRCC.

Overfitting often occurs in selecting prognostic models using higher-dimensional data [18], here, we chosen the LASSO algorithm, which could eliminate this disadvantage [19], as the method to select CpG for constructing epigenetic signature. The algorithm of LASSO has been widely used to construct predictive survival models, for instance, colon cancer [20], gastric cancer [21], T-cell lymphoblastic lymphoma [22] and ccRCC [9, 10, 23].

In recent years, epigenetics has been studied more and more in ccRCC, DNA methylation is one of the most studied patterns in epigenetic regulation [24–26]. And there have also been a number of studies using methylation data to construct prognostic signatures [5, 6, 27]. Here, we constructed and validated a 31-CpG-based epigenetic signature that could accurately predicted overall survival of ccRCC. Using the epigenetic signature we constructed, we divided patients into high and low risk groups, and high-risk patients had significantly lower overall survival rates in each set. Although previous studies have constructed several molecular biomarkers to predict the survival of ccRCC, few of the signature they have constructed are as accurate as our epigenetic signature. The AUC of time-dependent ROC was all greater than 0.75 in each set. After the epigenetic signature combined with staging and grading, the AUC predicted 5-year overall survival was reached 0.871. At the same time, we validated our epigenetic signature in different subgroups (pathologic stage, histologic grade, lymph node examination, and age) and found that the epigenetic signature showed excellent prognostic value.

We use ssGSEA and other methods to infer the functional annotation of epigenetic signature, and we found that the high risk group patients were mainly enriched in p53 and nod like receptor signaling pathway, which have been reported in previous studies to be associated with the pathogenesis of ccRCC [28–30]. The group of low risk were enriched in fatty acid metabolism and which has also been reported to be associated with ccRCC [31–33]. We speculate that these pathways may be responsible for the very different overall survival rates between the two groups based on the epigenetic signature. So what we’re going to do is to look at how do these pathways affect the prognosis of ccRCC.

We performed methylation sequencing on ccRCC tissue samples and kidney cancer cell lines, and the results showed that CpG islands involved in the construction of the prognosis model had significantly different expression patterns. However, our study has some limitations, we lack more data to verify it, but we have built a new biological sample database called Biological Repositories Zhongnan Hospital of Wuhan University (http://biobank.znhospital.cn), which will provide a lot of data to verify our epigenetic markers in the future.

In conclusion, we provide an effective epigenetic signature that accurately predicts overall survival of ccRCC independent of other risk factors (age, gender, neoadjuvant treatment, lymph node examination, histologic grade and pathologic stage). At the same time, we also constructed a nomogram for the convenience of clinicians to predict the prognosis of ccRCC.

## Conclusion

We developed a 31-CpG-based signature using bioinformatics methods (LASSO cox regression analysis). We found that this epigenetic signature could accurately predict the overall survival rate of ccRCC, contributing to the prediction of the prognosis of ccRCC. And we constructed a nomogram based on this epigenetic signature, which could accurately and conveniently predict the prognosis of ccRCC patients for clinicians.

## Supplementary information

**Additional file 1.** Supplementary tables 1–2, Supplementary Information: Related file 1. Ethics Committee Approval (number: 2020102).

**Additional file 2.** Supplementary figure S1: Functional annotation of the epigenetic signature.

**Additional file 3.** Supplementary figure S2: The PPI network diagram.

## Data Availability

The datasets analyzed during the current study are available in the Cancer Genome Atlas (TCGA) database (http://cancergenome.nih.gov/).

## Abbreviations

RCC: Renal cell carcinoma
ccRCC: Clear cell renal cell carcinoma
LASSO: Least absolute shrinkage and selection operator
TCGA: The cancer genome atlas
SNP: Single nucleotide polymorphism
DMP: Differentially methylated positions
FDR: False discovery rate
PPI: Protein–protein interaction
