# Supplementary material for: Epigenetic signature predicts overall survival clear cell renal cell carcinoma
Source: Cancer Cell Int. 2020 Nov 23;20:564. doi: 10.1186/s12935-020-01640-x (PMC7686748; doi:10.1186/s12935-020-01640-x)
Supplement: Supplementary file 1 — Additional file 1. Supplementary tables 1–2, Supplementary Information: Related file 1. Ethics Committee Approval (number: 2020102). [file 12935_2020_1640_MOESM1_ESM.docx]

**Epigenetic signature predicts overall survival clear cell renal cell carcinoma**

**Supplementary tables**

**Supplementary Table S1.** DMP analysis results.

| CpG ID | LogFC | t | P.Value | Adj.P.Val | Cancer_AVG | Normal_AVG | Deltaβ |
| --- | --- | --- | --- | --- | --- | --- | --- |
| cg20740711 | 0.29 | 64.9 | <0.001 | <0.001 | 0.06 | 0.35 | 0.29 |
| cg26063719 | 0.62 | 64.7 | <0.001 | <0.001 | 0.14 | 0.76 | 0.62 |
| cg12691620 | 0.25 | 61.4 | <0.001 | <0.001 | 0.05 | 0.30 | 0.25 |
| cg04456219 | 0.67 | 60.4 | <0.001 | <0.001 | 0.14 | 0.81 | 0.67 |
| cg16468729 | 0.40 | 60.1 | <0.001 | <0.001 | 0.09 | 0.49 | 0.40 |
| ch.10.119748781R | 0.37 | 58.5 | <0.001 | <0.001 | 0.07 | 0.44 | 0.37 |
| ch.16.97779F | 0.23 | 56.6 | <0.001 | <0.001 | 0.04 | 0.28 | 0.23 |
| cg20003368 | 0.34 | 55.3 | <0.001 | <0.001 | 0.05 | 0.39 | 0.34 |
| cg18973101 | 0.28 | 53.9 | <0.001 | <0.001 | 0.08 | 0.37 | 0.28 |
| cg01702055 | 0.64 | 53.3 | <0.001 | <0.001 | 0.19 | 0.83 | 0.64 |
| cg14204586 | 0.47 | 53.2 | <0.001 | <0.001 | 0.11 | 0.58 | 0.47 |
| cg03290131 | 0.49 | 52.1 | <0.001 | <0.001 | 0.08 | 0.57 | 0.49 |
| cg08141142 | -0.34 | -51.1 | <0.001 | <0.001 | 0.97 | 0.63 | -0.34 |
| cg06913958 | 0.25 | 50.5 | <0.001 | <0.001 | 0.07 | 0.32 | 0.25 |
| cg06021088 | 0.40 | 50.2 | <0.001 | <0.001 | 0.09 | 0.50 | 0.40 |
| cg15034300 | 0.30 | 50.2 | <0.001 | <0.001 | 0.08 | 0.39 | 0.30 |
| cg07677157 | 0.51 | 50.0 | <0.001 | <0.001 | 0.14 | 0.65 | 0.51 |
| cg27115863 | 0.37 | 49.7 | <0.001 | <0.001 | 0.09 | 0.46 | 0.37 |
| ch.1.171672612F | 0.22 | 49.0 | <0.001 | <0.001 | 0.09 | 0.31 | 0.22 |
| ch.16.82520294R | 0.21 | 49.0 | <0.001 | <0.001 | 0.07 | 0.28 | 0.21 |
| ch.20.50133246R | 0.28 | 48.8 | <0.001 | <0.001 | 0.11 | 0.38 | 0.28 |
| cg13294602 | -0.29 | -48.6 | <0.001 | <0.001 | 0.98 | 0.69 | -0.29 |
| cg23344780 | 0.29 | 48.6 | <0.001 | <0.001 | 0.08 | 0.37 | 0.29 |
| cg20968743 | 0.29 | 48.6 | <0.001 | <0.001 | 0.06 | 0.35 | 0.29 |
| cg19371349 | 0.33 | 48.5 | <0.001 | <0.001 | 0.09 | 0.43 | 0.33 |
| cg14601621 | -0.33 | -48.5 | <0.001 | <0.001 | 0.97 | 0.64 | -0.33 |
| cg13892570 | 0.30 | 48.4 | <0.001 | <0.001 | 0.06 | 0.36 | 0.30 |
| cg09029902 | 0.42 | 47.4 | <0.001 | <0.001 | 0.10 | 0.52 | 0.42 |
| ch.15.814613R | 0.20 | 47.4 | <0.001 | <0.001 | 0.07 | 0.27 | 0.20 |
| cg11588197 | 0.37 | 46.9 | <0.001 | <0.001 | 0.09 | 0.46 | 0.37 |
| cg23206160 | 0.33 | 46.8 | <0.001 | <0.001 | 0.13 | 0.45 | 0.33 |
| cg08995609 | 0.41 | 46.8 | <0.001 | <0.001 | 0.07 | 0.48 | 0.41 |
| ch.6.115952F | 0.25 | 46.8 | <0.001 | <0.001 | 0.08 | 0.33 | 0.25 |
| cg05963604 | 0.41 | 46.4 | <0.001 | <0.001 | 0.15 | 0.56 | 0.41 |
| ch.9.2223115R | 0.24 | 46.0 | <0.001 | <0.001 | 0.08 | 0.32 | 0.24 |
| cg13921921 | 0.45 | 46.0 | <0.001 | <0.001 | 0.12 | 0.57 | 0.45 |
| cg14189782 | 0.26 | 46.0 | <0.001 | <0.001 | 0.07 | 0.33 | 0.26 |
| cg13266096 | 0.31 | 45.8 | <0.001 | <0.001 | 0.07 | 0.38 | 0.31 |
| ch.15.934240F | 0.24 | 45.7 | <0.001 | <0.001 | 0.06 | 0.29 | 0.24 |
| cg03498081 | 0.54 | 45.7 | <0.001 | <0.001 | 0.13 | 0.67 | 0.54 |
| cg03063658 | 0.46 | 45.4 | <0.001 | <0.001 | 0.14 | 0.60 | 0.46 |
| cg01492656 | 0.34 | 45.3 | <0.001 | <0.001 | 0.07 | 0.41 | 0.34 |
| cg03851835 | 0.44 | 45.2 | <0.001 | <0.001 | 0.15 | 0.60 | 0.44 |
| cg25755851 | 0.41 | 45.0 | <0.001 | <0.001 | 0.16 | 0.57 | 0.41 |
| cg14856585 | 0.21 | 44.9 | <0.001 | <0.001 | 0.06 | 0.27 | 0.21 |
| cg16407699 | 0.41 | 44.8 | <0.001 | <0.001 | 0.14 | 0.55 | 0.41 |
| cg21708130 | 0.51 | 44.8 | <0.001 | <0.001 | 0.18 | 0.69 | 0.51 |
| cg03568017 | 0.20 | 44.7 | <0.001 | <0.001 | 0.07 | 0.27 | 0.20 |
| cg19163395 | 0.52 | 44.7 | <0.001 | <0.001 | 0.20 | 0.72 | 0.52 |
| cg05101437 | 0.39 | 44.6 | <0.001 | <0.001 | 0.05 | 0.44 | 0.39 |
| cg01815263 | 0.35 | 44.3 | <0.001 | <0.001 | 0.12 | 0.47 | 0.35 |
| cg20873416 | 0.41 | 44.3 | <0.001 | <0.001 | 0.09 | 0.50 | 0.41 |
| cg23680451 | 0.36 | 44.2 | <0.001 | <0.001 | 0.10 | 0.46 | 0.36 |
| cg10880902 | 0.41 | 43.9 | <0.001 | <0.001 | 0.19 | 0.59 | 0.41 |
| cg27407935 | 0.24 | 43.7 | <0.001 | <0.001 | 0.17 | 0.41 | 0.24 |
| cg21076680 | 0.42 | 43.4 | <0.001 | <0.001 | 0.19 | 0.61 | 0.42 |
| cg15832822 | 0.49 | 43.4 | <0.001 | <0.001 | 0.15 | 0.64 | 0.49 |
| cg02064267 | 0.27 | 43.3 | <0.001 | <0.001 | 0.16 | 0.43 | 0.27 |
| cg27300045 | 0.37 | 43.2 | <0.001 | <0.001 | 0.19 | 0.56 | 0.37 |
| cg26170244 | 0.33 | 42.9 | <0.001 | <0.001 | 0.06 | 0.39 | 0.33 |
| cg14665413 | 0.41 | 42.9 | <0.001 | <0.001 | 0.12 | 0.54 | 0.41 |
| cg23023970 | 0.36 | 42.7 | <0.001 | <0.001 | 0.18 | 0.54 | 0.36 |
| cg05068848 | 0.43 | 42.4 | <0.001 | <0.001 | 0.19 | 0.62 | 0.43 |
| cg20549620 | 0.33 | 42.4 | <0.001 | <0.001 | 0.14 | 0.47 | 0.33 |
| cg22793129 | 0.53 | 42.3 | <0.001 | <0.001 | 0.15 | 0.68 | 0.53 |
| cg06043315 | 0.21 | 42.2 | <0.001 | <0.001 | 0.07 | 0.28 | 0.21 |
| cg01904393 | 0.29 | 42.2 | <0.001 | <0.001 | 0.14 | 0.43 | 0.29 |
| ch.X.258064R | 0.21 | 42.2 | <0.001 | <0.001 | 0.08 | 0.29 | 0.21 |
| cg26954174 | 0.38 | 42.1 | <0.001 | <0.001 | 0.15 | 0.53 | 0.38 |
| cg10460946 | 0.38 | 41.8 | <0.001 | <0.001 | 0.19 | 0.57 | 0.38 |
| cg02389317 | 0.56 | 41.8 | <0.001 | <0.001 | 0.17 | 0.73 | 0.56 |
| cg17367884 | -0.27 | -41.8 | <0.001 | <0.001 | 0.93 | 0.66 | -0.27 |
| ch.3.1226245F | 0.24 | 41.7 | <0.001 | <0.001 | 0.05 | 0.29 | 0.24 |
| cg07094298 | 0.38 | 41.7 | <0.001 | <0.001 | 0.08 | 0.47 | 0.38 |
| cg26140475 | 0.34 | 41.7 | <0.001 | <0.001 | 0.10 | 0.44 | 0.34 |
| cg11171825 | 0.27 | 41.5 | <0.001 | <0.001 | 0.11 | 0.38 | 0.27 |
| cg12403889 | 0.32 | 41.5 | <0.001 | <0.001 | 0.11 | 0.43 | 0.32 |
| cg04797957 | 0.41 | 41.5 | <0.001 | <0.001 | 0.18 | 0.59 | 0.41 |
| cg24671734 | 0.48 | 41.4 | <0.001 | <0.001 | 0.19 | 0.67 | 0.48 |
| cg10536898 | -0.42 | -41.4 | <0.001 | <0.001 | 0.65 | 0.23 | -0.42 |
| cg12435551 | 0.41 | 41.3 | <0.001 | <0.001 | 0.06 | 0.47 | 0.41 |
| cg16107628 | 0.49 | 41.3 | <0.001 | <0.001 | 0.18 | 0.66 | 0.49 |
| cg07126235 | 0.36 | 41.3 | <0.001 | <0.001 | 0.06 | 0.42 | 0.36 |
| cg11993160 | 0.20 | 41.3 | <0.001 | <0.001 | 0.09 | 0.29 | 0.20 |
| cg10308629 | 0.28 | 41.2 | <0.001 | <0.001 | 0.09 | 0.37 | 0.28 |
| cg13289884 | 0.33 | 41.2 | <0.001 | <0.001 | 0.08 | 0.42 | 0.33 |
| cg21993290 | 0.26 | 41.2 | <0.001 | <0.001 | 0.20 | 0.46 | 0.26 |
| cg02515217 | 0.37 | 40.9 | <0.001 | <0.001 | 0.19 | 0.57 | 0.37 |
| cg07093324 | 0.44 | 40.9 | <0.001 | <0.001 | 0.18 | 0.63 | 0.44 |
| cg18700744 | 0.34 | 40.8 | <0.001 | <0.001 | 0.10 | 0.43 | 0.34 |
| cg11838152 | 0.38 | 40.7 | <0.001 | <0.001 | 0.17 | 0.55 | 0.38 |
| cg04999352 | 0.30 | 40.6 | <0.001 | <0.001 | 0.12 | 0.42 | 0.30 |
| cg02578087 | 0.50 | 40.6 | <0.001 | <0.001 | 0.15 | 0.65 | 0.50 |
| cg16411857 | 0.31 | 40.4 | <0.001 | <0.001 | 0.15 | 0.46 | 0.31 |
| cg00668559 | 0.21 | 40.4 | <0.001 | <0.001 | 0.15 | 0.36 | 0.21 |
| cg07283896 | 0.30 | 40.4 | <0.001 | <0.001 | 0.12 | 0.42 | 0.30 |
| cg04981492 | 0.22 | 40.3 | <0.001 | <0.001 | 0.05 | 0.28 | 0.22 |
| cg22579075 | 0.46 | 40.3 | <0.001 | <0.001 | 0.14 | 0.60 | 0.46 |
| cg07474842 | 0.44 | 40.3 | <0.001 | <0.001 | 0.13 | 0.57 | 0.44 |
| cg02072495 | 0.28 | 40.2 | <0.001 | <0.001 | 0.13 | 0.42 | 0.28 |
| cg03813377 | 0.26 | 40.2 | <0.001 | <0.001 | 0.17 | 0.43 | 0.26 |
| cg25135018 | 0.43 | 40.2 | <0.001 | <0.001 | 0.15 | 0.58 | 0.43 |
| cg22698489 | 0.24 | 40.2 | <0.001 | <0.001 | 0.04 | 0.28 | 0.24 |
| cg19789466 | 0.24 | 40.1 | <0.001 | <0.001 | 0.06 | 0.30 | 0.24 |
| cg21469505 | 0.27 | 40.0 | <0.001 | <0.001 | 0.14 | 0.41 | 0.27 |
| cg22373770 | 0.36 | 40.0 | <0.001 | <0.001 | 0.12 | 0.48 | 0.36 |
| cg01378159 | 0.31 | 40.0 | <0.001 | <0.001 | 0.08 | 0.39 | 0.31 |
| cg05673882 | 0.35 | 40.0 | <0.001 | <0.001 | 0.10 | 0.45 | 0.35 |
| cg01511901 | 0.39 | 40.0 | <0.001 | <0.001 | 0.17 | 0.57 | 0.39 |
| cg26853536 | 0.30 | 40.0 | <0.001 | <0.001 | 0.13 | 0.43 | 0.30 |
| cg12934258 | -0.40 | -39.9 | <0.001 | <0.001 | 0.88 | 0.49 | -0.40 |
| cg04276626 | 0.37 | 39.9 | <0.001 | <0.001 | 0.19 | 0.56 | 0.37 |
| cg02326386 | 0.65 | 39.9 | <0.001 | <0.001 | 0.19 | 0.85 | 0.65 |
| cg17983632 | -0.46 | -39.7 | <0.001 | <0.001 | 0.94 | 0.48 | -0.46 |
| cg13765004 | 0.23 | 39.6 | <0.001 | <0.001 | 0.07 | 0.31 | 0.23 |
| cg01298102 | 0.28 | 39.6 | <0.001 | <0.001 | 0.19 | 0.47 | 0.28 |
| cg13787850 | 0.43 | 39.6 | <0.001 | <0.001 | 0.15 | 0.58 | 0.43 |
| cg07235218 | 0.30 | 39.6 | <0.001 | <0.001 | 0.17 | 0.47 | 0.30 |
| cg18739537 | 0.33 | 39.6 | <0.001 | <0.001 | 0.13 | 0.45 | 0.33 |
| ch.16.2068605F | 0.33 | 39.5 | <0.001 | <0.001 | 0.15 | 0.48 | 0.33 |
| cg14488466 | 0.42 | 39.4 | <0.001 | <0.001 | 0.14 | 0.56 | 0.42 |
| cg00029282 | -0.34 | -39.3 | <0.001 | <0.001 | 0.93 | 0.59 | -0.34 |
| cg07267600 | 0.33 | 39.2 | <0.001 | <0.001 | 0.10 | 0.43 | 0.33 |
| cg21737444 | 0.39 | 39.2 | <0.001 | <0.001 | 0.16 | 0.55 | 0.39 |
| cg25874782 | 0.48 | 39.2 | <0.001 | <0.001 | 0.19 | 0.67 | 0.48 |
| cg01254505 | 0.28 | 39.1 | <0.001 | <0.001 | 0.05 | 0.33 | 0.28 |
| cg13568258 | 0.28 | 39.1 | <0.001 | <0.001 | 0.09 | 0.37 | 0.28 |
| cg06330323 | -0.43 | -39.1 | <0.001 | <0.001 | 0.82 | 0.39 | -0.43 |
| cg01889574 | 0.34 | 38.8 | <0.001 | <0.001 | 0.05 | 0.39 | 0.34 |
| cg11786870 | 0.34 | 38.8 | <0.001 | <0.001 | 0.11 | 0.46 | 0.34 |
| cg26559804 | 0.26 | 38.8 | <0.001 | <0.001 | 0.08 | 0.34 | 0.26 |
| cg16814143 | -0.22 | -38.8 | <0.001 | <0.001 | 0.94 | 0.72 | -0.22 |
| cg04650676 | 0.30 | 38.7 | <0.001 | <0.001 | 0.03 | 0.33 | 0.30 |
| cg02259081 | 0.34 | 38.6 | <0.001 | <0.001 | 0.19 | 0.53 | 0.34 |
| cg19319487 | 0.42 | 38.5 | <0.001 | <0.001 | 0.17 | 0.59 | 0.42 |
| cg05343811 | 0.49 | 38.5 | <0.001 | <0.001 | 0.10 | 0.59 | 0.49 |
| ch.10.2563868F | 0.22 | 38.5 | <0.001 | <0.001 | 0.13 | 0.36 | 0.22 |
| cg04511534 | -0.38 | -38.5 | <0.001 | <0.001 | 0.78 | 0.39 | -0.38 |
| cg11326574 | 0.25 | 38.2 | <0.001 | <0.001 | 0.18 | 0.43 | 0.25 |
| cg12510708 | 0.45 | 38.1 | <0.001 | <0.001 | 0.20 | 0.65 | 0.45 |
| cg07333191 | -0.43 | -38.0 | <0.001 | <0.001 | 0.62 | 0.19 | -0.43 |
| cg24638828 | 0.33 | 37.9 | <0.001 | <0.001 | 0.12 | 0.46 | 0.33 |
| cg17184704 | 0.48 | 37.9 | <0.001 | <0.001 | 0.14 | 0.62 | 0.48 |
| cg02117924 | -0.25 | -37.8 | <0.001 | <0.001 | 0.94 | 0.69 | -0.25 |
| cg04322486 | 0.32 | 37.7 | <0.001 | <0.001 | 0.17 | 0.48 | 0.32 |
| cg14137625 | -0.25 | -37.7 | <0.001 | <0.001 | 0.85 | 0.60 | -0.25 |
| cg06680511 | 0.28 | 37.7 | <0.001 | <0.001 | 0.12 | 0.39 | 0.28 |
| cg24876187 | 0.33 | 37.6 | <0.001 | <0.001 | 0.14 | 0.47 | 0.33 |
| cg02505676 | -0.25 | -37.6 | <0.001 | <0.001 | 0.89 | 0.65 | -0.25 |
| cg01181105 | -0.28 | -37.5 | <0.001 | <0.001 | 0.86 | 0.58 | -0.28 |
| cg01826354 | 0.30 | 37.4 | <0.001 | <0.001 | 0.13 | 0.43 | 0.30 |
| cg14391855 | 0.37 | 37.3 | <0.001 | <0.001 | 0.12 | 0.49 | 0.37 |
| cg19648552 | -0.39 | -37.2 | <0.001 | <0.001 | 0.89 | 0.50 | -0.39 |
| cg08698943 | 0.38 | 37.0 | <0.001 | <0.001 | 0.12 | 0.50 | 0.38 |
| cg11935248 | 0.28 | 36.8 | <0.001 | <0.001 | 0.08 | 0.36 | 0.28 |
| cg13324103 | 0.25 | 36.8 | <0.001 | <0.001 | 0.04 | 0.29 | 0.25 |
| cg07830160 | 0.40 | 36.8 | <0.001 | <0.001 | 0.08 | 0.49 | 0.40 |
| cg23683800 | 0.36 | 36.8 | <0.001 | <0.001 | 0.07 | 0.43 | 0.36 |
| cg17534029 | 0.38 | 36.7 | <0.001 | <0.001 | 0.09 | 0.47 | 0.38 |
| cg17107017 | -0.20 | -36.6 | <0.001 | <0.001 | 0.91 | 0.71 | -0.20 |
| cg09906233 | -0.21 | -36.6 | <0.001 | <0.001 | 0.88 | 0.67 | -0.21 |
| cg02782634 | 0.33 | 36.6 | <0.001 | <0.001 | 0.12 | 0.45 | 0.33 |
| cg12453675 | 0.21 | 36.6 | <0.001 | <0.001 | 0.10 | 0.32 | 0.21 |
| cg18760534 | -0.49 | -36.5 | <0.001 | <0.001 | 0.89 | 0.40 | -0.49 |
| cg10125703 | 0.24 | 36.5 | <0.001 | <0.001 | 0.10 | 0.34 | 0.24 |
| cg13468898 | -0.26 | -36.3 | <0.001 | <0.001 | 0.97 | 0.70 | -0.26 |
| cg19196335 | -0.30 | -36.3 | <0.001 | <0.001 | 0.88 | 0.59 | -0.30 |
| cg23950157 | 0.27 | 36.2 | <0.001 | <0.001 | 0.10 | 0.37 | 0.27 |
| cg21243631 | 0.24 | 36.0 | <0.001 | <0.001 | 0.15 | 0.39 | 0.24 |
| cg10502118 | 0.40 | 36.0 | <0.001 | <0.001 | 0.10 | 0.50 | 0.40 |
| cg24389585 | -0.28 | -35.9 | <0.001 | <0.001 | 0.86 | 0.57 | -0.28 |
| cg23787321 | 0.20 | 35.9 | <0.001 | <0.001 | 0.10 | 0.30 | 0.20 |
| cg00522276 | 0.27 | 35.9 | <0.001 | <0.001 | 0.11 | 0.38 | 0.27 |
| cg27638217 | 0.23 | 35.9 | <0.001 | <0.001 | 0.09 | 0.32 | 0.23 |
| cg19008097 | 0.34 | 35.9 | <0.001 | <0.001 | 0.17 | 0.51 | 0.34 |
| cg09716613 | 0.40 | 35.9 | <0.001 | <0.001 | 0.20 | 0.60 | 0.40 |
| cg07915516 | 0.22 | 35.8 | <0.001 | <0.001 | 0.17 | 0.40 | 0.22 |
| cg07336872 | 0.33 | 35.8 | <0.001 | <0.001 | 0.16 | 0.49 | 0.33 |
| cg15454698 | 0.32 | 35.8 | <0.001 | <0.001 | 0.15 | 0.47 | 0.32 |
| cg03466587 | 0.39 | 35.8 | <0.001 | <0.001 | 0.15 | 0.54 | 0.39 |
| cg01393234 | 0.30 | 35.8 | <0.001 | <0.001 | 0.18 | 0.48 | 0.30 |
| cg22790839 | 0.33 | 35.8 | <0.001 | <0.001 | 0.18 | 0.51 | 0.33 |
| cg02331902 | 0.21 | 35.6 | <0.001 | <0.001 | 0.07 | 0.28 | 0.21 |
| cg17096807 | 0.42 | 35.6 | <0.001 | <0.001 | 0.17 | 0.59 | 0.42 |
| cg13408344 | 0.32 | 35.6 | <0.001 | <0.001 | 0.10 | 0.42 | 0.32 |
| cg16704703 | 0.38 | 35.5 | <0.001 | <0.001 | 0.17 | 0.55 | 0.38 |
| cg11152384 | 0.42 | 35.5 | <0.001 | <0.001 | 0.12 | 0.54 | 0.42 |
| cg19284277 | 0.29 | 35.5 | <0.001 | <0.001 | 0.14 | 0.43 | 0.29 |
| cg10502324 | 0.26 | 35.4 | <0.001 | <0.001 | 0.06 | 0.32 | 0.26 |
| cg21499869 | 0.29 | 35.1 | <0.001 | <0.001 | 0.08 | 0.38 | 0.29 |
| cg04196298 | 0.33 | 35.1 | <0.001 | <0.001 | 0.11 | 0.43 | 0.33 |
| cg02753187 | 0.41 | 35.1 | <0.001 | <0.001 | 0.12 | 0.53 | 0.41 |
| cg10772185 | 0.41 | 35.1 | <0.001 | <0.001 | 0.12 | 0.52 | 0.41 |
| cg10777887 | 0.50 | 35.1 | <0.001 | <0.001 | 0.20 | 0.70 | 0.50 |
| cg05417332 | 0.32 | 35.1 | <0.001 | <0.001 | 0.17 | 0.49 | 0.32 |
| cg15994182 | -0.32 | -35.0 | <0.001 | <0.001 | 0.83 | 0.51 | -0.32 |
| cg22806907 | 0.35 | 34.9 | <0.001 | <0.001 | 0.04 | 0.39 | 0.35 |
| cg18677603 | 0.26 | 34.9 | <0.001 | <0.001 | 0.19 | 0.46 | 0.26 |
| cg18670236 | 0.52 | 34.9 | <0.001 | <0.001 | 0.19 | 0.71 | 0.52 |
| cg25479708 | 0.32 | 34.9 | <0.001 | <0.001 | 0.10 | 0.42 | 0.32 |
| cg25416067 | 0.32 | 34.9 | <0.001 | <0.001 | 0.04 | 0.36 | 0.32 |
| cg02849507 | 0.30 | 34.9 | <0.001 | <0.001 | 0.16 | 0.45 | 0.30 |
| cg00283857 | -0.43 | -34.8 | <0.001 | <0.001 | 0.92 | 0.49 | -0.43 |
| cg27019093 | -0.23 | -34.8 | <0.001 | <0.001 | 0.89 | 0.66 | -0.23 |
| cg03207666 | -0.32 | -34.8 | <0.001 | <0.001 | 0.86 | 0.55 | -0.32 |
| cg09650907 | 0.21 | 34.8 | <0.001 | <0.001 | 0.07 | 0.28 | 0.21 |
| cg19136632 | 0.25 | 34.8 | <0.001 | <0.001 | 0.11 | 0.37 | 0.25 |
| cg22598885 | -0.34 | -34.7 | <0.001 | <0.001 | 0.86 | 0.52 | -0.34 |
| cg16791781 | -0.34 | -34.7 | <0.001 | <0.001 | 0.82 | 0.48 | -0.34 |
| cg00713400 | -0.45 | -34.6 | <0.001 | <0.001 | 0.87 | 0.42 | -0.45 |
| cg00646731 | -0.31 | -34.5 | <0.001 | <0.001 | 0.87 | 0.56 | -0.31 |
| cg07904452 | 0.33 | 34.5 | <0.001 | <0.001 | 0.14 | 0.47 | 0.33 |
| cg01409343 | 0.23 | 34.4 | <0.001 | <0.001 | 0.13 | 0.36 | 0.23 |
| cg07171609 | 0.42 | 34.4 | <0.001 | <0.001 | 0.19 | 0.62 | 0.42 |
| cg01778994 | 0.38 | 34.3 | <0.001 | <0.001 | 0.12 | 0.50 | 0.38 |
| cg12265878 | 0.33 | 34.3 | <0.001 | <0.001 | 0.10 | 0.43 | 0.33 |
| cg25951430 | 0.34 | 34.3 | <0.001 | <0.001 | 0.10 | 0.43 | 0.34 |
| cg03816062 | -0.32 | -34.3 | <0.001 | <0.001 | 0.96 | 0.63 | -0.32 |
| cg10615591 | 0.36 | 34.2 | <0.001 | <0.001 | 0.13 | 0.49 | 0.36 |
| cg03819134 | 0.45 | 34.1 | <0.001 | <0.001 | 0.11 | 0.57 | 0.45 |
| cg23684410 | 0.22 | 34.1 | <0.001 | <0.001 | 0.17 | 0.38 | 0.22 |
| cg14882265 | -0.31 | -34.1 | <0.001 | <0.001 | 0.72 | 0.42 | -0.31 |
| cg10191240 | 0.46 | 34.1 | <0.001 | <0.001 | 0.19 | 0.65 | 0.46 |
| cg08918658 | 0.33 | 34.0 | <0.001 | <0.001 | 0.13 | 0.46 | 0.33 |
| cg08452327 | 0.31 | 34.0 | <0.001 | <0.001 | 0.09 | 0.39 | 0.31 |
| cg25343618 | 0.47 | 34.0 | <0.001 | <0.001 | 0.16 | 0.63 | 0.47 |
| cg05575213 | -0.24 | -33.9 | <0.001 | <0.001 | 0.97 | 0.74 | -0.24 |
| cg25095032 | -0.48 | -33.9 | <0.001 | <0.001 | 0.90 | 0.41 | -0.48 |
| cg14058329 | -0.32 | -33.9 | <0.001 | <0.001 | 0.84 | 0.52 | -0.32 |
| cg22413056 | 0.32 | 33.9 | <0.001 | <0.001 | 0.09 | 0.40 | 0.32 |
| cg08330247 | -0.27 | -33.7 | <0.001 | <0.001 | 0.95 | 0.68 | -0.27 |
| cg00722188 | -0.21 | -33.7 | <0.001 | <0.001 | 0.90 | 0.69 | -0.21 |
| cg27129922 | 0.25 | 33.7 | <0.001 | <0.001 | 0.12 | 0.38 | 0.25 |
| cg27658254 | 0.28 | 33.7 | <0.001 | <0.001 | 0.09 | 0.36 | 0.28 |
| cg24146125 | 0.28 | 33.6 | <0.001 | <0.001 | 0.16 | 0.44 | 0.28 |
| cg14129735 | 0.31 | 33.6 | <0.001 | <0.001 | 0.13 | 0.44 | 0.31 |
| cg05471169 | 0.31 | 33.6 | <0.001 | <0.001 | 0.07 | 0.38 | 0.31 |
| cg06747543 | 0.21 | 33.6 | <0.001 | <0.001 | 0.04 | 0.25 | 0.21 |
| cg09410512 | 0.36 | 33.6 | <0.001 | <0.001 | 0.10 | 0.46 | 0.36 |
| cg19131731 | 0.34 | 33.5 | <0.001 | <0.001 | 0.12 | 0.47 | 0.34 |
| cg06335867 | -0.44 | -33.5 | <0.001 | <0.001 | 0.62 | 0.18 | -0.44 |
| cg11536940 | 0.21 | 33.5 | <0.001 | <0.001 | 0.08 | 0.29 | 0.21 |
| cg08928696 | 0.38 | 33.4 | <0.001 | <0.001 | 0.09 | 0.47 | 0.38 |
| cg13454226 | 0.24 | 33.4 | <0.001 | <0.001 | 0.05 | 0.29 | 0.24 |
| cg15488978 | -0.40 | -33.4 | <0.001 | <0.001 | 0.94 | 0.55 | -0.40 |
| cg17272620 | 0.43 | 33.3 | <0.001 | <0.001 | 0.19 | 0.61 | 0.43 |
| cg22359581 | -0.22 | -33.3 | <0.001 | <0.001 | 0.91 | 0.69 | -0.22 |
| cg23821329 | 0.44 | 33.2 | <0.001 | <0.001 | 0.16 | 0.59 | 0.44 |
| cg20606255 | 0.21 | 33.1 | <0.001 | <0.001 | 0.08 | 0.29 | 0.21 |
| cg00587922 | 0.34 | 33.1 | <0.001 | <0.001 | 0.13 | 0.47 | 0.34 |
| cg18455390 | -0.32 | -33.1 | <0.001 | <0.001 | 0.86 | 0.55 | -0.32 |
| cg05697539 | -0.27 | -33.1 | <0.001 | <0.001 | 0.97 | 0.69 | -0.27 |
| cg02101833 | 0.33 | 33.1 | <0.001 | <0.001 | 0.18 | 0.51 | 0.33 |
| cg23877608 | 0.36 | 33.1 | <0.001 | <0.001 | 0.16 | 0.52 | 0.36 |
| cg13074866 | 0.22 | 33.0 | <0.001 | <0.001 | 0.11 | 0.33 | 0.22 |
| cg09380415 | -0.20 | -33.0 | <0.001 | <0.001 | 0.97 | 0.77 | -0.20 |
| cg13036352 | 0.38 | 33.0 | <0.001 | <0.001 | 0.12 | 0.50 | 0.38 |
| cg04337342 | -0.23 | -33.0 | <0.001 | <0.001 | 0.92 | 0.69 | -0.23 |
| cg06777902 | 0.32 | 33.0 | <0.001 | <0.001 | 0.17 | 0.48 | 0.32 |
| cg26758857 | 0.23 | 32.9 | <0.001 | <0.001 | 0.06 | 0.29 | 0.23 |
| cg26650359 | 0.24 | 32.9 | <0.001 | <0.001 | 0.11 | 0.34 | 0.24 |
| cg03171300 | 0.25 | 32.9 | <0.001 | <0.001 | 0.19 | 0.45 | 0.25 |
| cg26389380 | 0.28 | 32.9 | <0.001 | <0.001 | 0.13 | 0.41 | 0.28 |
| cg05956452 | -0.43 | -32.9 | <0.001 | <0.001 | 0.93 | 0.50 | -0.43 |
| cg02225720 | 0.41 | 32.9 | <0.001 | <0.001 | 0.17 | 0.58 | 0.41 |
| cg20262021 | -0.28 | -32.9 | <0.001 | <0.001 | 0.89 | 0.62 | -0.28 |
| cg17110767 | -0.21 | -32.8 | <0.001 | <0.001 | 0.91 | 0.70 | -0.21 |
| cg00218409 | 0.25 | 32.8 | <0.001 | <0.001 | 0.13 | 0.38 | 0.25 |
| cg22641201 | 0.21 | 32.8 | <0.001 | <0.001 | 0.08 | 0.29 | 0.21 |
| cg04465078 | 0.41 | 32.7 | <0.001 | <0.001 | 0.20 | 0.61 | 0.41 |
| cg06933824 | -0.30 | -32.6 | <0.001 | <0.001 | 0.79 | 0.49 | -0.30 |
| cg25518868 | 0.24 | 32.6 | <0.001 | <0.001 | 0.07 | 0.32 | 0.24 |
| cg12864389 | 0.37 | 32.6 | <0.001 | <0.001 | 0.15 | 0.52 | 0.37 |
| cg03339910 | 0.31 | 32.6 | <0.001 | <0.001 | 0.20 | 0.51 | 0.31 |
| cg11027217 | -0.22 | -32.6 | <0.001 | <0.001 | 0.91 | 0.69 | -0.22 |
| cg19770748 | -0.28 | -32.5 | <0.001 | <0.001 | 0.94 | 0.66 | -0.28 |
| cg27132471 | 0.45 | 32.5 | <0.001 | <0.001 | 0.13 | 0.59 | 0.45 |
| cg02849693 | -0.42 | -32.5 | <0.001 | <0.001 | 0.63 | 0.22 | -0.42 |
| cg17508941 | -0.35 | -32.5 | <0.001 | <0.001 | 0.64 | 0.29 | -0.35 |
| cg21966860 | -0.25 | -32.5 | <0.001 | <0.001 | 0.96 | 0.71 | -0.25 |
| cg01323381 | -0.37 | -32.5 | <0.001 | <0.001 | 0.81 | 0.44 | -0.37 |
| cg14570632 | -0.22 | -32.5 | <0.001 | <0.001 | 0.92 | 0.70 | -0.22 |
| cg16550264 | 0.40 | 32.5 | <0.001 | <0.001 | 0.18 | 0.58 | 0.40 |
| cg14043253 | 0.32 | 32.5 | <0.001 | <0.001 | 0.10 | 0.42 | 0.32 |
| cg21435684 | 0.37 | 32.4 | <0.001 | <0.001 | 0.07 | 0.44 | 0.37 |
| cg11084334 | -0.26 | -32.4 | <0.001 | <0.001 | 0.76 | 0.49 | -0.26 |
| cg21410293 | -0.26 | -32.4 | <0.001 | <0.001 | 0.92 | 0.65 | -0.26 |
| cg17501395 | 0.33 | 32.4 | <0.001 | <0.001 | 0.13 | 0.46 | 0.33 |
| cg19748485 | -0.21 | -32.3 | <0.001 | <0.001 | 0.62 | 0.41 | -0.21 |
| cg08692006 | 0.22 | 32.3 | <0.001 | <0.001 | 0.13 | 0.35 | 0.22 |
| cg24935121 | 0.24 | 32.3 | <0.001 | <0.001 | 0.14 | 0.38 | 0.24 |
| cg04786142 | 0.47 | 32.3 | <0.001 | <0.001 | 0.11 | 0.59 | 0.47 |
| cg15243034 | -0.35 | -32.3 | <0.001 | <0.001 | 0.77 | 0.42 | -0.35 |
| cg11736020 | 0.21 | 32.2 | <0.001 | <0.001 | 0.07 | 0.29 | 0.21 |
| cg05247640 | 0.21 | 32.2 | <0.001 | <0.001 | 0.08 | 0.29 | 0.21 |
| cg07911961 | 0.34 | 32.2 | <0.001 | <0.001 | 0.20 | 0.54 | 0.34 |
| cg19687152 | -0.39 | -32.2 | <0.001 | <0.001 | 0.93 | 0.54 | -0.39 |
| cg25986727 | -0.23 | -32.2 | <0.001 | <0.001 | 0.86 | 0.63 | -0.23 |
| cg05876246 | 0.39 | 32.1 | <0.001 | <0.001 | 0.10 | 0.49 | 0.39 |
| cg27295118 | 0.26 | 32.1 | <0.001 | <0.001 | 0.16 | 0.41 | 0.26 |
| cg04659689 | 0.35 | 32.0 | <0.001 | <0.001 | 0.09 | 0.44 | 0.35 |
| cg25883405 | 0.27 | 32.0 | <0.001 | <0.001 | 0.13 | 0.40 | 0.27 |
| cg01288184 | 0.41 | 31.9 | <0.001 | <0.001 | 0.08 | 0.49 | 0.41 |
| cg09880291 | -0.30 | -31.9 | <0.001 | <0.001 | 0.88 | 0.58 | -0.30 |
| cg00448761 | 0.21 | 31.9 | <0.001 | <0.001 | 0.09 | 0.30 | 0.21 |
| cg01318557 | 0.33 | 31.8 | <0.001 | <0.001 | 0.13 | 0.45 | 0.33 |
| cg15013617 | 0.36 | 31.8 | <0.001 | <0.001 | 0.17 | 0.53 | 0.36 |
| cg25296938 | 0.37 | 31.7 | <0.001 | <0.001 | 0.13 | 0.50 | 0.37 |
| cg00969405 | -0.33 | -31.7 | <0.001 | <0.001 | 0.83 | 0.49 | -0.33 |
| cg00950718 | 0.29 | 31.6 | <0.001 | <0.001 | 0.10 | 0.39 | 0.29 |
| cg24367957 | 0.34 | 31.6 | <0.001 | <0.001 | 0.15 | 0.50 | 0.34 |
| cg00842351 | 0.24 | 31.6 | <0.001 | <0.001 | 0.06 | 0.30 | 0.24 |
| cg01065210 | -0.24 | -31.5 | <0.001 | <0.001 | 0.88 | 0.64 | -0.24 |
| cg18082788 | 0.36 | 31.5 | <0.001 | <0.001 | 0.07 | 0.43 | 0.36 |
| cg22933582 | -0.23 | -31.4 | <0.001 | <0.001 | 0.85 | 0.62 | -0.23 |
| cg19239199 | 0.45 | 31.4 | <0.001 | <0.001 | 0.13 | 0.58 | 0.45 |
| cg21956614 | 0.22 | 31.4 | <0.001 | <0.001 | 0.06 | 0.28 | 0.22 |
| cg14037250 | -0.22 | -31.4 | <0.001 | <0.001 | 0.92 | 0.70 | -0.22 |
| cg15555527 | -0.54 | -31.4 | <0.001 | <0.001 | 0.79 | 0.25 | -0.54 |
| cg02779592 | 0.37 | 31.4 | <0.001 | <0.001 | 0.12 | 0.49 | 0.37 |
| cg16148593 | 0.24 | 31.4 | <0.001 | <0.001 | 0.09 | 0.33 | 0.24 |
| cg21118367 | 0.27 | 31.3 | <0.001 | <0.001 | 0.05 | 0.32 | 0.27 |
| cg21918595 | 0.21 | 31.3 | <0.001 | <0.001 | 0.15 | 0.37 | 0.21 |
| cg24348240 | 0.39 | 31.3 | <0.001 | <0.001 | 0.18 | 0.56 | 0.39 |
| cg12575511 | -0.20 | -31.3 | <0.001 | <0.001 | 0.92 | 0.72 | -0.20 |
| cg07733247 | -0.33 | -31.3 | <0.001 | <0.001 | 0.85 | 0.53 | -0.33 |
| cg24704287 | 0.29 | 31.2 | <0.001 | <0.001 | 0.11 | 0.40 | 0.29 |
| cg04090697 | -0.34 | -31.2 | <0.001 | <0.001 | 0.84 | 0.50 | -0.34 |
| cg02924487 | -0.26 | -31.2 | <0.001 | <0.001 | 0.78 | 0.51 | -0.26 |
| cg03000596 | 0.34 | 31.2 | <0.001 | <0.001 | 0.18 | 0.52 | 0.34 |
| cg19937878 | -0.34 | -31.2 | <0.001 | <0.001 | 0.86 | 0.53 | -0.34 |
| cg03110787 | -0.21 | -31.2 | <0.001 | <0.001 | 0.84 | 0.63 | -0.21 |
| cg06161697 | -0.24 | -31.1 | <0.001 | <0.001 | 0.90 | 0.67 | -0.24 |
| cg06670039 | -0.42 | -31.0 | <0.001 | <0.001 | 0.95 | 0.53 | -0.42 |
| cg11382133 | 0.33 | 31.0 | <0.001 | <0.001 | 0.12 | 0.45 | 0.33 |
| cg18640536 | -0.26 | -31.0 | <0.001 | <0.001 | 0.88 | 0.62 | -0.26 |
| cg00259834 | 0.43 | 31.0 | <0.001 | <0.001 | 0.15 | 0.58 | 0.43 |
| cg02139853 | -0.23 | -31.0 | <0.001 | <0.001 | 0.96 | 0.72 | -0.23 |
| cg15101245 | 0.38 | 30.9 | <0.001 | <0.001 | 0.15 | 0.52 | 0.38 |
| cg05209483 | 0.36 | 30.9 | <0.001 | <0.001 | 0.09 | 0.45 | 0.36 |
| cg12307314 | 0.22 | 30.9 | <0.001 | <0.001 | 0.19 | 0.41 | 0.22 |
| cg05398036 | 0.39 | 30.9 | <0.001 | <0.001 | 0.16 | 0.55 | 0.39 |
| cg09244707 | -0.21 | -30.9 | <0.001 | <0.001 | 0.75 | 0.54 | -0.21 |
| cg21641458 | -0.25 | -30.9 | <0.001 | <0.001 | 0.74 | 0.49 | -0.25 |
| cg22985172 | -0.23 | -30.9 | <0.001 | <0.001 | 0.87 | 0.63 | -0.23 |
| cg26403843 | 0.27 | 30.8 | <0.001 | <0.001 | 0.15 | 0.42 | 0.27 |
| cg20949223 | -0.27 | -30.8 | <0.001 | <0.001 | 0.95 | 0.68 | -0.27 |
| cg00004996 | -0.21 | -30.8 | <0.001 | <0.001 | 0.85 | 0.64 | -0.21 |
| cg11664818 | -0.25 | -30.8 | <0.001 | <0.001 | 0.76 | 0.51 | -0.25 |
| cg21249595 | -0.24 | -30.8 | <0.001 | <0.001 | 0.80 | 0.55 | -0.24 |
| cg02590287 | 0.26 | 30.7 | <0.001 | <0.001 | 0.15 | 0.41 | 0.26 |
| cg07165066 | 0.44 | 30.7 | <0.001 | <0.001 | 0.18 | 0.62 | 0.44 |
| cg06875255 | -0.28 | -30.7 | <0.001 | <0.001 | 0.79 | 0.51 | -0.28 |
| cg16649560 | 0.30 | 30.7 | <0.001 | <0.001 | 0.16 | 0.46 | 0.30 |
| cg13978542 | -0.22 | -30.7 | <0.001 | <0.001 | 0.83 | 0.61 | -0.22 |
| cg27647370 | -0.22 | -30.7 | <0.001 | <0.001 | 0.90 | 0.68 | -0.22 |
| cg10061342 | 0.32 | 30.7 | <0.001 | <0.001 | 0.15 | 0.47 | 0.32 |
| cg21571658 | 0.43 | 30.7 | <0.001 | <0.001 | 0.13 | 0.55 | 0.43 |
| cg09535960 | 0.20 | 30.6 | <0.001 | <0.001 | 0.08 | 0.29 | 0.20 |
| cg02458516 | 0.35 | 30.6 | <0.001 | <0.001 | 0.18 | 0.53 | 0.35 |
| cg25225655 | -0.26 | -30.6 | <0.001 | <0.001 | 0.86 | 0.60 | -0.26 |
| cg12570716 | 0.24 | 30.5 | <0.001 | <0.001 | 0.18 | 0.42 | 0.24 |
| cg20978460 | -0.39 | -30.5 | <0.001 | <0.001 | 0.93 | 0.54 | -0.39 |
| cg17432857 | -0.36 | -30.5 | <0.001 | <0.001 | 0.79 | 0.43 | -0.36 |
| cg07156249 | 0.49 | 30.5 | <0.001 | <0.001 | 0.19 | 0.68 | 0.49 |
| cg17565051 | -0.36 | -30.5 | <0.001 | <0.001 | 0.92 | 0.57 | -0.36 |
| cg04663932 | 0.22 | 30.5 | <0.001 | <0.001 | 0.09 | 0.31 | 0.22 |
| cg15995714 | -0.25 | -30.5 | <0.001 | <0.001 | 0.79 | 0.54 | -0.25 |
| cg27016272 | -0.26 | -30.5 | <0.001 | <0.001 | 0.88 | 0.62 | -0.26 |
| cg00876266 | -0.26 | -30.4 | <0.001 | <0.001 | 0.97 | 0.71 | -0.26 |
| cg14370448 | 0.33 | 30.4 | <0.001 | <0.001 | 0.17 | 0.49 | 0.33 |
| cg21064451 | 0.23 | 30.4 | <0.001 | <0.001 | 0.19 | 0.42 | 0.23 |
| cg03619586 | 0.20 | 30.4 | <0.001 | <0.001 | 0.06 | 0.27 | 0.20 |
| cg10657965 | -0.27 | -30.4 | <0.001 | <0.001 | 0.96 | 0.69 | -0.27 |
| cg23521140 | 0.22 | 30.3 | <0.001 | <0.001 | 0.04 | 0.26 | 0.22 |
| cg07463059 | 0.39 | 30.3 | <0.001 | <0.001 | 0.10 | 0.49 | 0.39 |
| cg10268345 | -0.30 | -30.3 | <0.001 | <0.001 | 0.95 | 0.65 | -0.30 |
| cg00771593 | -0.28 | -30.3 | <0.001 | <0.001 | 0.84 | 0.56 | -0.28 |
| cg19412669 | 0.32 | 30.3 | <0.001 | <0.001 | 0.13 | 0.45 | 0.32 |
| cg17694130 | 0.20 | 30.2 | <0.001 | <0.001 | 0.04 | 0.24 | 0.20 |
| cg24471894 | 0.22 | 30.2 | <0.001 | <0.001 | 0.11 | 0.33 | 0.22 |
| cg12807330 | -0.30 | -30.1 | <0.001 | <0.001 | 0.92 | 0.61 | -0.30 |
| cg04012053 | -0.24 | -30.1 | <0.001 | <0.001 | 0.94 | 0.70 | -0.24 |
| cg00718444 | -0.22 | -30.1 | <0.001 | <0.001 | 0.90 | 0.68 | -0.22 |
| cg21978694 | -0.38 | -30.1 | <0.001 | <0.001 | 0.92 | 0.54 | -0.38 |
| cg13985198 | 0.25 | 30.1 | <0.001 | <0.001 | 0.06 | 0.31 | 0.25 |
| cg00243187 | -0.31 | -30.1 | <0.001 | <0.001 | 0.96 | 0.65 | -0.31 |
| cg04270048 | 0.23 | 30.0 | <0.001 | <0.001 | 0.11 | 0.34 | 0.23 |
| cg09642408 | -0.30 | -30.0 | <0.001 | <0.001 | 0.89 | 0.59 | -0.30 |
| cg11285912 | -0.31 | -29.9 | <0.001 | <0.001 | 0.86 | 0.55 | -0.31 |
| cg07661480 | -0.29 | -29.9 | <0.001 | <0.001 | 0.76 | 0.47 | -0.29 |
| cg26091247 | 0.28 | 29.9 | <0.001 | <0.001 | 0.13 | 0.41 | 0.28 |
| cg26351132 | 0.20 | 29.9 | <0.001 | <0.001 | 0.13 | 0.34 | 0.20 |
| cg14312114 | -0.30 | -29.9 | <0.001 | <0.001 | 0.87 | 0.57 | -0.30 |
| cg05774699 | -0.28 | -29.9 | <0.001 | <0.001 | 0.75 | 0.47 | -0.28 |
| cg02153855 | 0.23 | 29.8 | <0.001 | <0.001 | 0.18 | 0.41 | 0.23 |
| cg01651570 | -0.28 | -29.8 | <0.001 | <0.001 | 0.93 | 0.65 | -0.28 |
| cg20617957 | 0.23 | 29.8 | <0.001 | <0.001 | 0.10 | 0.33 | 0.23 |
| cg10517290 | 0.24 | 29.8 | <0.001 | <0.001 | 0.11 | 0.35 | 0.24 |
| cg18851100 | -0.34 | -29.8 | <0.001 | <0.001 | 0.76 | 0.42 | -0.34 |
| cg07537821 | 0.31 | 29.8 | <0.001 | <0.001 | 0.18 | 0.49 | 0.31 |
| cg22226527 | 0.27 | 29.8 | <0.001 | <0.001 | 0.12 | 0.39 | 0.27 |
| cg22899502 | -0.27 | -29.8 | <0.001 | <0.001 | 0.91 | 0.64 | -0.27 |
| cg07166409 | 0.25 | 29.8 | <0.001 | <0.001 | 0.09 | 0.34 | 0.25 |
| cg03540794 | -0.31 | -29.8 | <0.001 | <0.001 | 0.87 | 0.56 | -0.31 |
| cg04406115 | -0.27 | -29.8 | <0.001 | <0.001 | 0.87 | 0.60 | -0.27 |
| cg20945738 | 0.29 | 29.8 | <0.001 | <0.001 | 0.10 | 0.40 | 0.29 |
| cg26074025 | 0.21 | 29.8 | <0.001 | <0.001 | 0.13 | 0.34 | 0.21 |
| cg19209689 | -0.38 | -29.7 | <0.001 | <0.001 | 0.65 | 0.27 | -0.38 |
| cg17888985 | 0.36 | 29.7 | <0.001 | <0.001 | 0.19 | 0.55 | 0.36 |
| cg01583716 | 0.24 | 29.7 | <0.001 | <0.001 | 0.13 | 0.38 | 0.24 |
| cg08783616 | 0.24 | 29.7 | <0.001 | <0.001 | 0.18 | 0.42 | 0.24 |
| cg08409642 | 0.33 | 29.7 | <0.001 | <0.001 | 0.12 | 0.45 | 0.33 |
| cg03624316 | 0.27 | 29.7 | <0.001 | <0.001 | 0.17 | 0.45 | 0.27 |
| cg01758106 | -0.22 | -29.7 | <0.001 | <0.001 | 0.82 | 0.60 | -0.22 |
| cg00177787 | -0.40 | -29.7 | <0.001 | <0.001 | 0.93 | 0.53 | -0.40 |
| cg12858593 | 0.24 | 29.6 | <0.001 | <0.001 | 0.19 | 0.43 | 0.24 |
| cg15444217 | -0.27 | -29.6 | <0.001 | <0.001 | 0.87 | 0.59 | -0.27 |
| cg12249345 | -0.36 | -29.5 | <0.001 | <0.001 | 0.92 | 0.56 | -0.36 |
| cg13767779 | -0.23 | -29.4 | <0.001 | <0.001 | 0.85 | 0.62 | -0.23 |
| cg13175329 | 0.28 | 29.4 | <0.001 | <0.001 | 0.11 | 0.39 | 0.28 |
| cg26023912 | -0.25 | -29.4 | <0.001 | <0.001 | 0.69 | 0.44 | -0.25 |
| cg17476701 | 0.32 | 29.4 | <0.001 | <0.001 | 0.11 | 0.44 | 0.32 |
| cg24702147 | -0.37 | -29.3 | <0.001 | <0.001 | 0.93 | 0.56 | -0.37 |
| cg12141030 | 0.22 | 29.3 | <0.001 | <0.001 | 0.14 | 0.36 | 0.22 |
| cg08554554 | 0.32 | 29.3 | <0.001 | <0.001 | 0.18 | 0.51 | 0.32 |
| cg02107844 | 0.30 | 29.3 | <0.001 | <0.001 | 0.18 | 0.48 | 0.30 |
| cg04164048 | -0.27 | -29.3 | <0.001 | <0.001 | 0.87 | 0.60 | -0.27 |
| cg14415629 | 0.30 | 29.3 | <0.001 | <0.001 | 0.14 | 0.44 | 0.30 |
| cg02632314 | 0.20 | 29.2 | <0.001 | <0.001 | 0.14 | 0.34 | 0.20 |
| cg14930633 | 0.22 | 29.2 | <0.001 | <0.001 | 0.19 | 0.40 | 0.22 |
| cg19236431 | 0.22 | 29.1 | <0.001 | <0.001 | 0.16 | 0.38 | 0.22 |
| cg02734600 | -0.21 | -29.1 | <0.001 | <0.001 | 0.73 | 0.52 | -0.21 |
| cg06530983 | 0.20 | 29.1 | <0.001 | <0.001 | 0.07 | 0.27 | 0.20 |
| cg06968912 | 0.25 | 29.1 | <0.001 | <0.001 | 0.14 | 0.39 | 0.25 |
| cg14013695 | -0.39 | -29.1 | <0.001 | <0.001 | 0.81 | 0.42 | -0.39 |
| cg02951526 | -0.32 | -29.1 | <0.001 | <0.001 | 0.78 | 0.46 | -0.32 |
| cg01425293 | -0.23 | -29.1 | <0.001 | <0.001 | 0.87 | 0.64 | -0.23 |
| cg09632273 | -0.40 | -29.1 | <0.001 | <0.001 | 0.87 | 0.47 | -0.40 |
| cg12182580 | -0.28 | -29.1 | <0.001 | <0.001 | 0.82 | 0.54 | -0.28 |
| ch.2.11889418R | 0.24 | 29.1 | <0.001 | <0.001 | 0.17 | 0.41 | 0.24 |
| cg18003518 | -0.31 | -29.1 | <0.001 | <0.001 | 0.94 | 0.63 | -0.31 |
| cg13536080 | 0.31 | 29.1 | <0.001 | <0.001 | 0.07 | 0.39 | 0.31 |
| cg19084059 | -0.21 | -29.1 | <0.001 | <0.001 | 0.93 | 0.72 | -0.21 |
| cg16893634 | -0.21 | -29.0 | <0.001 | <0.001 | 0.89 | 0.68 | -0.21 |
| cg00808170 | -0.20 | -29.0 | <0.001 | <0.001 | 0.65 | 0.44 | -0.20 |
| cg26189303 | 0.41 | 28.9 | <0.001 | <0.001 | 0.16 | 0.57 | 0.41 |
| cg23475955 | -0.36 | -28.9 | <0.001 | <0.001 | 0.85 | 0.49 | -0.36 |
| cg06211255 | 0.22 | 28.9 | <0.001 | <0.001 | 0.19 | 0.41 | 0.22 |
| cg00868875 | -0.37 | -28.9 | <0.001 | <0.001 | 0.91 | 0.54 | -0.37 |
| cg17198308 | 0.25 | 28.9 | <0.001 | <0.001 | 0.03 | 0.29 | 0.25 |
| cg15220605 | -0.22 | -28.9 | <0.001 | <0.001 | 0.94 | 0.72 | -0.22 |
| cg02821998 | -0.25 | -28.9 | <0.001 | <0.001 | 0.64 | 0.39 | -0.25 |
| cg26549174 | 0.25 | 28.8 | <0.001 | <0.001 | 0.09 | 0.34 | 0.25 |
| cg13597544 | -0.24 | -28.8 | <0.001 | <0.001 | 0.83 | 0.59 | -0.24 |
| cg23678594 | 0.38 | 28.8 | <0.001 | <0.001 | 0.19 | 0.57 | 0.38 |
| cg06654691 | 0.42 | 28.8 | <0.001 | <0.001 | 0.15 | 0.57 | 0.42 |
| cg06148264 | 0.21 | 28.8 | <0.001 | <0.001 | 0.12 | 0.33 | 0.21 |
| cg06867623 | -0.27 | -28.7 | <0.001 | <0.001 | 0.92 | 0.65 | -0.27 |
| cg16224951 | 0.32 | 28.7 | <0.001 | <0.001 | 0.19 | 0.52 | 0.32 |
| cg15371617 | 0.31 | 28.7 | <0.001 | <0.001 | 0.12 | 0.43 | 0.31 |
| cg10852165 | 0.25 | 28.7 | <0.001 | <0.001 | 0.17 | 0.42 | 0.25 |
| cg26395694 | 0.29 | 28.7 | <0.001 | <0.001 | 0.13 | 0.42 | 0.29 |
| cg09744051 | 0.25 | 28.7 | <0.001 | <0.001 | 0.11 | 0.36 | 0.25 |
| cg21330896 | 0.36 | 28.6 | <0.001 | <0.001 | 0.08 | 0.44 | 0.36 |
| cg15366555 | -0.27 | -28.6 | <0.001 | <0.001 | 0.70 | 0.44 | -0.27 |
| cg14114546 | 0.26 | 28.6 | <0.001 | <0.001 | 0.14 | 0.40 | 0.26 |
| cg15978565 | 0.33 | 28.6 | <0.001 | <0.001 | 0.16 | 0.49 | 0.33 |
| cg01397065 | -0.32 | -28.5 | <0.001 | <0.001 | 0.80 | 0.49 | -0.32 |
| cg10064871 | 0.23 | 28.5 | <0.001 | <0.001 | 0.13 | 0.36 | 0.23 |
| cg07499182 | 0.27 | 28.5 | <0.001 | <0.001 | 0.09 | 0.36 | 0.27 |
| cg26063904 | 0.37 | 28.5 | <0.001 | <0.001 | 0.19 | 0.56 | 0.37 |
| cg00311883 | 0.30 | 28.5 | <0.001 | <0.001 | 0.09 | 0.39 | 0.30 |
| cg26603656 | 0.41 | 28.4 | <0.001 | <0.001 | 0.13 | 0.54 | 0.41 |
| cg13329912 | 0.23 | 28.4 | <0.001 | <0.001 | 0.11 | 0.34 | 0.23 |
| cg12615903 | -0.20 | -28.4 | <0.001 | <0.001 | 0.84 | 0.64 | -0.20 |
| cg03368099 | -0.29 | -28.4 | <0.001 | <0.001 | 0.61 | 0.33 | -0.29 |
| cg27566403 | -0.32 | -28.3 | <0.001 | <0.001 | 0.68 | 0.37 | -0.32 |
| cg05545635 | -0.28 | -28.3 | <0.001 | <0.001 | 0.92 | 0.65 | -0.28 |
| cg20005705 | -0.28 | -28.3 | <0.001 | <0.001 | 0.82 | 0.55 | -0.28 |
| cg08376141 | -0.28 | -28.3 | <0.001 | <0.001 | 0.63 | 0.36 | -0.28 |
| cg12161971 | -0.25 | -28.3 | <0.001 | <0.001 | 0.94 | 0.69 | -0.25 |
| cg17962854 | 0.21 | 28.2 | <0.001 | <0.001 | 0.11 | 0.32 | 0.21 |
| cg00370106 | 0.25 | 28.2 | <0.001 | <0.001 | 0.12 | 0.37 | 0.25 |
| cg06112654 | 0.20 | 28.2 | <0.001 | <0.001 | 0.12 | 0.32 | 0.20 |
| cg16552822 | 0.28 | 28.1 | <0.001 | <0.001 | 0.09 | 0.37 | 0.28 |
| cg07485775 | -0.20 | -28.1 | <0.001 | <0.001 | 0.84 | 0.64 | -0.20 |
| cg27429749 | 0.41 | 28.1 | <0.001 | <0.001 | 0.19 | 0.60 | 0.41 |
| cg16588163 | 0.34 | 28.1 | <0.001 | <0.001 | 0.20 | 0.54 | 0.34 |
| cg25506432 | -0.32 | -28.1 | <0.001 | <0.001 | 0.78 | 0.46 | -0.32 |
| cg14599440 | 0.23 | 28.1 | <0.001 | <0.001 | 0.17 | 0.40 | 0.23 |
| cg20202112 | -0.33 | -28.0 | <0.001 | <0.001 | 0.94 | 0.61 | -0.33 |
| cg20161965 | -0.34 | -28.0 | <0.001 | <0.001 | 0.87 | 0.53 | -0.34 |
| cg10888900 | 0.38 | 28.0 | <0.001 | <0.001 | 0.18 | 0.57 | 0.38 |
| cg09292244 | -0.40 | -28.0 | <0.001 | <0.001 | 0.79 | 0.39 | -0.40 |
| cg09269891 | -0.24 | -28.0 | <0.001 | <0.001 | 0.74 | 0.50 | -0.24 |
| cg13136655 | 0.33 | 28.0 | <0.001 | <0.001 | 0.15 | 0.48 | 0.33 |
| cg11741753 | -0.23 | -28.0 | <0.001 | <0.001 | 0.78 | 0.55 | -0.23 |
| cg14697657 | 0.20 | 28.0 | <0.001 | <0.001 | 0.16 | 0.36 | 0.20 |
| cg18939260 | -0.21 | -27.9 | <0.001 | <0.001 | 0.89 | 0.68 | -0.21 |
| cg21672572 | 0.35 | 27.8 | <0.001 | <0.001 | 0.19 | 0.54 | 0.35 |
| cg25258233 | 0.35 | 27.8 | <0.001 | <0.001 | 0.12 | 0.46 | 0.35 |
| cg08775375 | -0.35 | -27.8 | <0.001 | <0.001 | 0.87 | 0.52 | -0.35 |
| cg22731981 | -0.24 | -27.8 | <0.001 | <0.001 | 0.89 | 0.64 | -0.24 |
| cg07539798 | -0.24 | -27.8 | <0.001 | <0.001 | 0.68 | 0.43 | -0.24 |
| cg12935350 | -0.23 | -27.8 | <0.001 | <0.001 | 0.90 | 0.67 | -0.23 |
| cg00073090 | 0.23 | 27.8 | <0.001 | <0.001 | 0.17 | 0.39 | 0.23 |
| cg16238149 | 0.22 | 27.7 | <0.001 | <0.001 | 0.16 | 0.37 | 0.22 |
| cg23348155 | -0.28 | -27.7 | <0.001 | <0.001 | 0.88 | 0.59 | -0.28 |
| cg20544808 | -0.35 | -27.7 | <0.001 | <0.001 | 0.83 | 0.48 | -0.35 |
| cg05638347 | -0.24 | -27.7 | <0.001 | <0.001 | 0.92 | 0.68 | -0.24 |
| cg13763287 | -0.20 | -27.6 | <0.001 | <0.001 | 0.98 | 0.77 | -0.20 |
| cg11935143 | 0.23 | 27.6 | <0.001 | <0.001 | 0.19 | 0.43 | 0.23 |
| cg27151303 | -0.29 | -27.6 | <0.001 | <0.001 | 0.71 | 0.42 | -0.29 |
| cg02596427 | -0.21 | -27.6 | <0.001 | <0.001 | 0.76 | 0.55 | -0.21 |
| cg14312063 | -0.32 | -27.6 | <0.001 | <0.001 | 0.66 | 0.34 | -0.32 |
| cg04819655 | -0.28 | -27.6 | <0.001 | <0.001 | 0.88 | 0.60 | -0.28 |
| cg05674437 | -0.37 | -27.6 | <0.001 | <0.001 | 0.88 | 0.51 | -0.37 |
| cg14783993 | 0.21 | 27.6 | <0.001 | <0.001 | 0.14 | 0.35 | 0.21 |
| cg25919098 | -0.25 | -27.6 | <0.001 | <0.001 | 0.86 | 0.61 | -0.25 |
| cg23037321 | 0.22 | 27.6 | <0.001 | <0.001 | 0.07 | 0.30 | 0.22 |
| cg02116864 | -0.24 | -27.5 | <0.001 | <0.001 | 0.74 | 0.50 | -0.24 |
| cg22589169 | 0.20 | 27.5 | <0.001 | <0.001 | 0.12 | 0.32 | 0.20 |
| cg23099587 | 0.30 | 27.5 | <0.001 | <0.001 | 0.08 | 0.38 | 0.30 |
| cg11539055 | -0.40 | -27.5 | <0.001 | <0.001 | 0.90 | 0.49 | -0.40 |
| cg26978172 | -0.36 | -27.5 | <0.001 | <0.001 | 0.91 | 0.55 | -0.36 |
| cg10632765 | -0.22 | -27.5 | <0.001 | <0.001 | 0.95 | 0.73 | -0.22 |
| cg07153966 | -0.30 | -27.5 | <0.001 | <0.001 | 0.79 | 0.49 | -0.30 |
| cg06014057 | -0.32 | -27.5 | <0.001 | <0.001 | 0.84 | 0.52 | -0.32 |
| cg09113070 | 0.24 | 27.5 | <0.001 | <0.001 | 0.18 | 0.42 | 0.24 |
| cg22611900 | -0.28 | -27.5 | <0.001 | <0.001 | 0.84 | 0.55 | -0.28 |
| cg01373189 | 0.28 | 27.5 | <0.001 | <0.001 | 0.16 | 0.44 | 0.28 |
| cg02475283 | -0.22 | -27.5 | <0.001 | <0.001 | 0.85 | 0.64 | -0.22 |
| cg21067652 | 0.20 | 27.4 | <0.001 | <0.001 | 0.12 | 0.32 | 0.20 |
| cg22514764 | -0.23 | -27.4 | <0.001 | <0.001 | 0.94 | 0.71 | -0.23 |
| cg22648929 | -0.20 | -27.4 | <0.001 | <0.001 | 0.96 | 0.76 | -0.20 |
| cg26166817 | -0.34 | -27.4 | <0.001 | <0.001 | 0.93 | 0.59 | -0.34 |
| cg21324456 | 0.26 | 27.4 | <0.001 | <0.001 | 0.14 | 0.40 | 0.26 |
| cg25075776 | -0.24 | -27.4 | <0.001 | <0.001 | 0.80 | 0.57 | -0.24 |
| cg06593910 | -0.26 | -27.4 | <0.001 | <0.001 | 0.94 | 0.69 | -0.26 |
| cg22969397 | -0.25 | -27.3 | <0.001 | <0.001 | 0.89 | 0.64 | -0.25 |
| cg04846710 | 0.31 | 27.3 | <0.001 | <0.001 | 0.12 | 0.43 | 0.31 |
| cg20597486 | 0.47 | 27.3 | <0.001 | <0.001 | 0.17 | 0.64 | 0.47 |
| cg20155035 | 0.24 | 27.3 | <0.001 | <0.001 | 0.18 | 0.42 | 0.24 |
| cg16048383 | -0.29 | -27.3 | <0.001 | <0.001 | 0.80 | 0.51 | -0.29 |
| cg01497747 | -0.24 | -27.3 | <0.001 | <0.001 | 0.76 | 0.53 | -0.24 |
| cg17868538 | -0.24 | -27.2 | <0.001 | <0.001 | 0.86 | 0.62 | -0.24 |
| cg17406248 | -0.25 | -27.2 | <0.001 | <0.001 | 0.85 | 0.61 | -0.25 |
| cg01407254 | -0.33 | -27.2 | <0.001 | <0.001 | 0.94 | 0.60 | -0.33 |
| cg21010202 | -0.26 | -27.2 | <0.001 | <0.001 | 0.92 | 0.66 | -0.26 |
| cg26731008 | -0.22 | -27.2 | <0.001 | <0.001 | 0.72 | 0.49 | -0.22 |
| cg13591848 | -0.27 | -27.2 | <0.001 | <0.001 | 0.88 | 0.61 | -0.27 |
| cg00824156 | -0.22 | -27.2 | <0.001 | <0.001 | 0.85 | 0.63 | -0.22 |
| cg05349039 | -0.26 | -27.2 | <0.001 | <0.001 | 0.75 | 0.49 | -0.26 |
| cg06746774 | -0.21 | -27.1 | <0.001 | <0.001 | 0.96 | 0.75 | -0.21 |
| cg23222604 | 0.21 | 27.1 | <0.001 | <0.001 | 0.16 | 0.37 | 0.21 |
| cg09678939 | 0.39 | 27.1 | <0.001 | <0.001 | 0.18 | 0.57 | 0.39 |
| cg27449114 | 0.23 | 27.1 | <0.001 | <0.001 | 0.12 | 0.35 | 0.23 |
| cg23689080 | -0.22 | -27.1 | <0.001 | <0.001 | 0.72 | 0.51 | -0.22 |
| cg16997642 | -0.30 | -27.1 | <0.001 | <0.001 | 0.88 | 0.58 | -0.30 |
| cg23077820 | -0.21 | -27.1 | <0.001 | <0.001 | 0.79 | 0.58 | -0.21 |
| cg14131536 | -0.28 | -27.0 | <0.001 | <0.001 | 0.88 | 0.60 | -0.28 |
| cg05271255 | 0.22 | 27.0 | <0.001 | <0.001 | 0.15 | 0.37 | 0.22 |
| cg16112766 | -0.25 | -27.0 | <0.001 | <0.001 | 0.86 | 0.61 | -0.25 |
| cg06614044 | -0.34 | -27.0 | <0.001 | <0.001 | 0.72 | 0.38 | -0.34 |
| cg14016554 | -0.28 | -27.0 | <0.001 | <0.001 | 0.75 | 0.47 | -0.28 |
| cg12845051 | -0.31 | -27.0 | <0.001 | <0.001 | 0.77 | 0.46 | -0.31 |
| cg00294109 | 0.22 | 27.0 | <0.001 | <0.001 | 0.07 | 0.29 | 0.22 |
| cg08554257 | 0.23 | 27.0 | <0.001 | <0.001 | 0.06 | 0.29 | 0.23 |
| cg01979888 | 0.26 | 26.9 | <0.001 | <0.001 | 0.08 | 0.34 | 0.26 |
| cg00542992 | 0.23 | 26.9 | <0.001 | <0.001 | 0.19 | 0.42 | 0.23 |
| cg02092141 | -0.34 | -26.9 | <0.001 | <0.001 | 0.85 | 0.51 | -0.34 |
| cg15826479 | 0.20 | 26.9 | <0.001 | <0.001 | 0.18 | 0.38 | 0.20 |
| cg03503942 | 0.27 | 26.9 | <0.001 | <0.001 | 0.11 | 0.38 | 0.27 |
| cg27064337 | 0.29 | 26.9 | <0.001 | <0.001 | 0.18 | 0.46 | 0.29 |
| cg19254118 | -0.44 | -26.9 | <0.001 | <0.001 | 0.86 | 0.42 | -0.44 |
| cg22891413 | -0.27 | -26.9 | <0.001 | <0.001 | 0.90 | 0.63 | -0.27 |
| cg19794481 | -0.20 | -26.9 | <0.001 | <0.001 | 0.93 | 0.73 | -0.20 |
| cg23314826 | -0.30 | -26.9 | <0.001 | <0.001 | 0.89 | 0.59 | -0.30 |
| cg06976598 | 0.39 | 26.8 | <0.001 | <0.001 | 0.20 | 0.58 | 0.39 |
| cg10841756 | -0.33 | -26.8 | <0.001 | <0.001 | 0.92 | 0.59 | -0.33 |
| cg02537838 | -0.34 | -26.8 | <0.001 | <0.001 | 0.82 | 0.47 | -0.34 |
| cg09354263 | -0.21 | -26.8 | <0.001 | <0.001 | 0.79 | 0.58 | -0.21 |
| cg07797693 | -0.27 | -26.8 | <0.001 | <0.001 | 0.89 | 0.62 | -0.27 |
| cg22827210 | 0.26 | 26.8 | <0.001 | <0.001 | 0.14 | 0.41 | 0.26 |
| cg11732134 | -0.29 | -26.8 | <0.001 | <0.001 | 0.93 | 0.64 | -0.29 |
| cg26662324 | 0.21 | 26.8 | <0.001 | <0.001 | 0.11 | 0.32 | 0.21 |
| cg12593515 | -0.30 | -26.8 | <0.001 | <0.001 | 0.73 | 0.43 | -0.30 |
| cg23480985 | 0.21 | 26.8 | <0.001 | <0.001 | 0.14 | 0.35 | 0.21 |
| cg07151443 | 0.23 | 26.7 | <0.001 | <0.001 | 0.11 | 0.34 | 0.23 |
| cg10316474 | 0.22 | 26.7 | <0.001 | <0.001 | 0.13 | 0.34 | 0.22 |
| cg17485681 | -0.28 | -26.7 | <0.001 | <0.001 | 0.71 | 0.43 | -0.28 |
| cg21821308 | -0.50 | -26.7 | <0.001 | <0.001 | 0.72 | 0.23 | -0.50 |
| cg17277615 | -0.25 | -26.7 | <0.001 | <0.001 | 0.81 | 0.57 | -0.25 |
| cg04792034 | -0.20 | -26.6 | <0.001 | <0.001 | 0.89 | 0.69 | -0.20 |
| cg14658493 | -0.29 | -26.6 | <0.001 | <0.001 | 0.88 | 0.58 | -0.29 |
| cg11558551 | 0.22 | 26.6 | <0.001 | <0.001 | 0.06 | 0.28 | 0.22 |
| cg02439266 | -0.24 | -26.6 | <0.001 | <0.001 | 0.95 | 0.70 | -0.24 |
| cg26402169 | -0.30 | -26.6 | <0.001 | <0.001 | 0.92 | 0.62 | -0.30 |
| cg09143801 | -0.38 | -26.6 | <0.001 | <0.001 | 0.82 | 0.44 | -0.38 |
| cg05676622 | -0.28 | -26.6 | <0.001 | <0.001 | 0.92 | 0.64 | -0.28 |
| cg26811385 | 0.26 | 26.5 | <0.001 | <0.001 | 0.12 | 0.38 | 0.26 |
| cg02184281 | -0.24 | -26.5 | <0.001 | <0.001 | 0.82 | 0.59 | -0.24 |
| cg04583813 | -0.34 | -26.4 | <0.001 | <0.001 | 0.91 | 0.57 | -0.34 |
| cg07340025 | 0.35 | 26.4 | <0.001 | <0.001 | 0.18 | 0.52 | 0.35 |
| cg19777396 | -0.27 | -26.4 | <0.001 | <0.001 | 0.80 | 0.52 | -0.27 |
| cg09245698 | -0.21 | -26.4 | <0.001 | <0.001 | 0.79 | 0.58 | -0.21 |
| cg14052728 | -0.26 | -26.4 | <0.001 | <0.001 | 0.95 | 0.69 | -0.26 |
| cg04838832 | 0.22 | 26.4 | <0.001 | <0.001 | 0.05 | 0.27 | 0.22 |
| cg16059943 | -0.23 | -26.4 | <0.001 | <0.001 | 0.86 | 0.63 | -0.23 |
| cg10050410 | -0.35 | -26.4 | <0.001 | <0.001 | 0.90 | 0.56 | -0.35 |
| cg04860563 | -0.29 | -26.4 | <0.001 | <0.001 | 0.88 | 0.58 | -0.29 |
| cg18792131 | -0.43 | -26.3 | <0.001 | <0.001 | 0.92 | 0.50 | -0.43 |
| cg06885175 | 0.25 | 26.3 | <0.001 | <0.001 | 0.17 | 0.42 | 0.25 |
| cg24580782 | -0.23 | -26.3 | <0.001 | <0.001 | 0.91 | 0.68 | -0.23 |
| cg17401780 | -0.23 | -26.3 | <0.001 | <0.001 | 0.83 | 0.60 | -0.23 |
| cg01919011 | -0.33 | -26.3 | <0.001 | <0.001 | 0.90 | 0.58 | -0.33 |
| cg18178715 | 0.37 | 26.3 | <0.001 | <0.001 | 0.18 | 0.55 | 0.37 |
| cg04917446 | -0.35 | -26.3 | <0.001 | <0.001 | 0.67 | 0.32 | -0.35 |
| cg03280622 | 0.28 | 26.3 | <0.001 | <0.001 | 0.19 | 0.46 | 0.28 |
| cg16291917 | -0.31 | -26.3 | <0.001 | <0.001 | 0.80 | 0.50 | -0.31 |
| cg01561869 | -0.28 | -26.3 | <0.001 | <0.001 | 0.70 | 0.42 | -0.28 |
| cg14995475 | -0.31 | -26.3 | <0.001 | <0.001 | 0.74 | 0.43 | -0.31 |
| cg19008099 | -0.25 | -26.2 | <0.001 | <0.001 | 0.79 | 0.54 | -0.25 |
| cg22660299 | -0.33 | -26.2 | <0.001 | <0.001 | 0.90 | 0.57 | -0.33 |
| cg04901835 | 0.27 | 26.2 | <0.001 | <0.001 | 0.19 | 0.46 | 0.27 |
| cg18738906 | -0.28 | -26.2 | <0.001 | <0.001 | 0.91 | 0.63 | -0.28 |
| cg03744763 | -0.23 | -26.2 | <0.001 | <0.001 | 0.64 | 0.41 | -0.23 |
| cg04074004 | -0.31 | -26.2 | <0.001 | <0.001 | 0.90 | 0.59 | -0.31 |
| cg06161600 | 0.23 | 26.2 | <0.001 | <0.001 | 0.16 | 0.40 | 0.23 |
| cg23698269 | 0.34 | 26.2 | <0.001 | <0.001 | 0.17 | 0.51 | 0.34 |
| cg27375286 | -0.20 | -26.2 | <0.001 | <0.001 | 0.74 | 0.54 | -0.20 |
| cg01562537 | -0.24 | -26.1 | <0.001 | <0.001 | 0.85 | 0.61 | -0.24 |
| cg26568226 | -0.22 | -26.1 | <0.001 | <0.001 | 0.92 | 0.70 | -0.22 |
| cg24677093 | -0.27 | -26.1 | <0.001 | <0.001 | 0.95 | 0.67 | -0.27 |
| cg15840891 | 0.35 | 26.1 | <0.001 | <0.001 | 0.16 | 0.51 | 0.35 |
| cg26651950 | 0.29 | 26.1 | <0.001 | <0.001 | 0.18 | 0.47 | 0.29 |
| cg20251943 | -0.30 | -26.1 | <0.001 | <0.001 | 0.79 | 0.49 | -0.30 |
| cg21429725 | -0.31 | -26.1 | <0.001 | <0.001 | 0.94 | 0.63 | -0.31 |
| cg21241151 | -0.26 | -26.1 | <0.001 | <0.001 | 0.79 | 0.53 | -0.26 |
| cg03852144 | 0.34 | 26.0 | <0.001 | <0.001 | 0.12 | 0.46 | 0.34 |
| cg27064482 | -0.41 | -26.0 | <0.001 | <0.001 | 0.65 | 0.24 | -0.41 |
| cg21830368 | -0.21 | -26.0 | <0.001 | <0.001 | 0.82 | 0.62 | -0.21 |
| cg12193277 | -0.20 | -26.0 | <0.001 | <0.001 | 0.79 | 0.59 | -0.20 |
| cg04388901 | -0.33 | -26.0 | <0.001 | <0.001 | 0.66 | 0.33 | -0.33 |
| cg12737588 | -0.24 | -25.9 | <0.001 | <0.001 | 0.95 | 0.71 | -0.24 |
| cg16865965 | 0.34 | 25.9 | <0.001 | <0.001 | 0.09 | 0.43 | 0.34 |
| cg18188010 | 0.23 | 25.9 | <0.001 | <0.001 | 0.18 | 0.41 | 0.23 |
| cg00989538 | -0.33 | -25.9 | <0.001 | <0.001 | 0.86 | 0.53 | -0.33 |
| cg19115610 | 0.26 | 25.9 | <0.001 | <0.001 | 0.13 | 0.39 | 0.26 |
| cg24873695 | -0.29 | -25.9 | <0.001 | <0.001 | 0.90 | 0.62 | -0.29 |
| cg11203397 | 0.32 | 25.9 | <0.001 | <0.001 | 0.11 | 0.42 | 0.32 |
| cg19580810 | -0.30 | -25.9 | <0.001 | <0.001 | 0.82 | 0.53 | -0.30 |
| cg04444771 | 0.23 | 25.9 | <0.001 | <0.001 | 0.14 | 0.37 | 0.23 |
| cg05336051 | 0.26 | 25.9 | <0.001 | <0.001 | 0.09 | 0.34 | 0.26 |
| cg24227481 | 0.30 | 25.9 | <0.001 | <0.001 | 0.16 | 0.46 | 0.30 |
| cg24422029 | -0.29 | -25.9 | <0.001 | <0.001 | 0.86 | 0.57 | -0.29 |
| cg09396704 | -0.23 | -25.9 | <0.001 | <0.001 | 0.60 | 0.37 | -0.23 |
| cg16414472 | -0.29 | -25.8 | <0.001 | <0.001 | 0.93 | 0.64 | -0.29 |
| cg02017450 | 0.21 | 25.8 | <0.001 | <0.001 | 0.11 | 0.32 | 0.21 |
| cg11414821 | 0.29 | 25.8 | <0.001 | <0.001 | 0.12 | 0.41 | 0.29 |
| cg06767142 | -0.27 | -25.8 | <0.001 | <0.001 | 0.92 | 0.64 | -0.27 |
| cg05127924 | -0.31 | -25.8 | <0.001 | <0.001 | 0.72 | 0.41 | -0.31 |
| cg25647415 | -0.21 | -25.8 | <0.001 | <0.001 | 0.80 | 0.59 | -0.21 |
| cg00612828 | -0.29 | -25.8 | <0.001 | <0.001 | 0.92 | 0.63 | -0.29 |
| cg21785832 | -0.39 | -25.8 | <0.001 | <0.001 | 0.66 | 0.27 | -0.39 |
| cg09321019 | -0.24 | -25.8 | <0.001 | <0.001 | 0.91 | 0.67 | -0.24 |
| cg14293129 | -0.26 | -25.7 | <0.001 | <0.001 | 0.87 | 0.61 | -0.26 |
| cg16763885 | 0.22 | 25.7 | <0.001 | <0.001 | 0.11 | 0.33 | 0.22 |
| cg04398547 | -0.30 | -25.7 | <0.001 | <0.001 | 0.84 | 0.55 | -0.30 |
| cg24440302 | 0.27 | 25.7 | <0.001 | <0.001 | 0.09 | 0.36 | 0.27 |
| cg04865531 | -0.27 | -25.7 | <0.001 | <0.001 | 0.77 | 0.50 | -0.27 |
| cg06942649 | 0.27 | 25.7 | <0.001 | <0.001 | 0.11 | 0.38 | 0.27 |
| cg05801374 | -0.32 | -25.7 | <0.001 | <0.001 | 0.85 | 0.53 | -0.32 |
| cg07529658 | -0.43 | -25.6 | <0.001 | <0.001 | 0.86 | 0.43 | -0.43 |
| cg26698460 | -0.32 | -25.6 | <0.001 | <0.001 | 0.72 | 0.39 | -0.32 |
| cg07303143 | 0.27 | 25.6 | <0.001 | <0.001 | 0.17 | 0.43 | 0.27 |
| cg05493841 | 0.22 | 25.6 | <0.001 | <0.001 | 0.06 | 0.28 | 0.22 |
| cg16273943 | -0.20 | -25.6 | <0.001 | <0.001 | 0.83 | 0.62 | -0.20 |
| cg24635860 | 0.27 | 25.5 | <0.001 | <0.001 | 0.19 | 0.47 | 0.27 |
| cg18460575 | -0.28 | -25.5 | <0.001 | <0.001 | 0.81 | 0.53 | -0.28 |
| cg14398860 | -0.43 | -25.5 | <0.001 | <0.001 | 0.92 | 0.49 | -0.43 |
| cg16657152 | 0.28 | 25.5 | <0.001 | <0.001 | 0.19 | 0.47 | 0.28 |
| cg24720717 | 0.26 | 25.5 | <0.001 | <0.001 | 0.04 | 0.30 | 0.26 |
| cg11100804 | -0.26 | -25.5 | <0.001 | <0.001 | 0.73 | 0.46 | -0.26 |
| cg06873916 | -0.31 | -25.5 | <0.001 | <0.001 | 0.71 | 0.40 | -0.31 |
| cg21308365 | 0.26 | 25.5 | <0.001 | <0.001 | 0.19 | 0.45 | 0.26 |
| cg11052535 | 0.20 | 25.5 | <0.001 | <0.001 | 0.18 | 0.38 | 0.20 |
| cg27217474 | -0.30 | -25.5 | <0.001 | <0.001 | 0.72 | 0.42 | -0.30 |
| cg16026813 | -0.33 | -25.5 | <0.001 | <0.001 | 0.88 | 0.55 | -0.33 |
| cg04513861 | -0.23 | -25.4 | <0.001 | <0.001 | 0.79 | 0.56 | -0.23 |
| cg26055950 | -0.26 | -25.4 | <0.001 | <0.001 | 0.64 | 0.39 | -0.26 |
| cg27052183 | -0.29 | -25.4 | <0.001 | <0.001 | 0.74 | 0.45 | -0.29 |
| cg13225830 | -0.28 | -25.4 | <0.001 | <0.001 | 0.92 | 0.64 | -0.28 |
| cg14397231 | 0.24 | 25.4 | <0.001 | <0.001 | 0.13 | 0.37 | 0.24 |
| cg02247178 | -0.29 | -25.3 | <0.001 | <0.001 | 0.72 | 0.43 | -0.29 |
| cg16282892 | 0.32 | 25.3 | <0.001 | <0.001 | 0.15 | 0.47 | 0.32 |
| cg14584926 | -0.23 | -25.3 | <0.001 | <0.001 | 0.83 | 0.60 | -0.23 |
| cg25390165 | -0.22 | -25.3 | <0.001 | <0.001 | 0.69 | 0.47 | -0.22 |
| cg11440915 | -0.22 | -25.3 | <0.001 | <0.001 | 0.82 | 0.60 | -0.22 |
| cg25241038 | -0.25 | -25.2 | <0.001 | <0.001 | 0.89 | 0.65 | -0.25 |
| cg08070327 | -0.29 | -25.2 | <0.001 | <0.001 | 0.86 | 0.57 | -0.29 |
| cg06757585 | -0.35 | -25.2 | <0.001 | <0.001 | 0.61 | 0.26 | -0.35 |
| cg26363039 | 0.27 | 25.2 | <0.001 | <0.001 | 0.15 | 0.42 | 0.27 |
| cg12149609 | 0.24 | 25.2 | <0.001 | <0.001 | 0.19 | 0.43 | 0.24 |
| cg02840697 | 0.21 | 25.2 | <0.001 | <0.001 | 0.10 | 0.31 | 0.21 |
| cg04033022 | -0.22 | -25.2 | <0.001 | <0.001 | 0.84 | 0.62 | -0.22 |
| cg08726522 | 0.24 | 25.1 | <0.001 | <0.001 | 0.15 | 0.39 | 0.24 |
| cg07134230 | 0.26 | 25.1 | <0.001 | <0.001 | 0.07 | 0.33 | 0.26 |
| cg00834924 | 0.20 | 25.1 | <0.001 | <0.001 | 0.18 | 0.38 | 0.20 |
| cg12686441 | 0.37 | 25.1 | <0.001 | <0.001 | 0.16 | 0.52 | 0.37 |
| cg13425135 | -0.21 | -25.1 | <0.001 | <0.001 | 0.96 | 0.74 | -0.21 |
| cg17110920 | -0.24 | -25.1 | <0.001 | <0.001 | 0.74 | 0.50 | -0.24 |
| cg19788754 | -0.27 | -25.1 | <0.001 | <0.001 | 0.87 | 0.60 | -0.27 |
| cg25748441 | 0.24 | 25.1 | <0.001 | <0.001 | 0.08 | 0.31 | 0.24 |
| cg09790270 | 0.25 | 25.1 | <0.001 | <0.001 | 0.18 | 0.43 | 0.25 |
| cg15888472 | -0.27 | -25.1 | <0.001 | <0.001 | 0.71 | 0.44 | -0.27 |
| cg17803589 | -0.23 | -25.1 | <0.001 | <0.001 | 0.79 | 0.56 | -0.23 |
| cg17873048 | -0.31 | -25.0 | <0.001 | <0.001 | 0.86 | 0.55 | -0.31 |
| cg25098793 | -0.29 | -25.0 | <0.001 | <0.001 | 0.83 | 0.53 | -0.29 |
| cg09179249 | -0.33 | -25.0 | <0.001 | <0.001 | 0.85 | 0.52 | -0.33 |
| cg18005337 | -0.21 | -25.0 | <0.001 | <0.001 | 0.90 | 0.69 | -0.21 |
| cg05628549 | 0.23 | 25.0 | <0.001 | <0.001 | 0.06 | 0.29 | 0.23 |
| cg21962450 | 0.23 | 25.0 | <0.001 | <0.001 | 0.09 | 0.32 | 0.23 |
| cg26200585 | -0.23 | -25.0 | <0.001 | <0.001 | 0.81 | 0.58 | -0.23 |
| cg17779676 | -0.27 | -24.9 | <0.001 | <0.001 | 0.79 | 0.51 | -0.27 |
| cg21488279 | -0.23 | -24.9 | <0.001 | <0.001 | 0.60 | 0.37 | -0.23 |
| cg13428086 | -0.25 | -24.9 | <0.001 | <0.001 | 0.84 | 0.59 | -0.25 |
| cg09591524 | -0.23 | -24.9 | <0.001 | <0.001 | 0.95 | 0.71 | -0.23 |
| cg12656312 | 0.24 | 24.9 | <0.001 | <0.001 | 0.17 | 0.41 | 0.24 |
| cg22229016 | -0.24 | -24.9 | <0.001 | <0.001 | 0.87 | 0.64 | -0.24 |
| cg23480341 | 0.25 | 24.8 | <0.001 | <0.001 | 0.18 | 0.43 | 0.25 |
| cg21660130 | -0.26 | -24.8 | <0.001 | <0.001 | 0.86 | 0.60 | -0.26 |
| cg00399027 | -0.21 | -24.8 | <0.001 | <0.001 | 0.86 | 0.65 | -0.21 |
| cg18085683 | -0.34 | -24.8 | <0.001 | <0.001 | 0.87 | 0.53 | -0.34 |
| cg27646729 | -0.30 | -24.8 | <0.001 | <0.001 | 0.79 | 0.50 | -0.30 |
| cg04477010 | -0.21 | -24.8 | <0.001 | <0.001 | 0.95 | 0.74 | -0.21 |
| cg22424903 | -0.24 | -24.8 | <0.001 | <0.001 | 0.79 | 0.55 | -0.24 |
| cg00863099 | 0.25 | 24.8 | <0.001 | <0.001 | 0.08 | 0.33 | 0.25 |
| cg17117243 | -0.32 | -24.8 | <0.001 | <0.001 | 0.74 | 0.41 | -0.32 |
| cg07890238 | -0.35 | -24.7 | <0.001 | <0.001 | 0.82 | 0.47 | -0.35 |
| cg00327697 | 0.24 | 24.7 | <0.001 | <0.001 | 0.12 | 0.36 | 0.24 |
| cg10043155 | -0.28 | -24.7 | <0.001 | <0.001 | 0.92 | 0.63 | -0.28 |
| cg00622799 | 0.23 | 24.7 | <0.001 | <0.001 | 0.18 | 0.41 | 0.23 |
| cg04396312 | -0.22 | -24.6 | <0.001 | <0.001 | 0.89 | 0.67 | -0.22 |
| cg21002651 | 0.23 | 24.6 | <0.001 | <0.001 | 0.05 | 0.28 | 0.23 |
| cg22030032 | 0.22 | 24.6 | <0.001 | <0.001 | 0.12 | 0.34 | 0.22 |
| cg24948792 | -0.20 | -24.6 | <0.001 | <0.001 | 0.86 | 0.66 | -0.20 |
| cg18538668 | 0.23 | 24.6 | <0.001 | <0.001 | 0.19 | 0.42 | 0.23 |
| cg10259265 | 0.27 | 24.6 | <0.001 | <0.001 | 0.19 | 0.45 | 0.27 |
| cg08396985 | -0.33 | -24.6 | <0.001 | <0.001 | 0.85 | 0.52 | -0.33 |
| cg25338563 | 0.31 | 24.6 | <0.001 | <0.001 | 0.14 | 0.45 | 0.31 |
| cg10579705 | 0.21 | 24.6 | <0.001 | <0.001 | 0.14 | 0.34 | 0.21 |
| cg05670885 | -0.21 | -24.6 | <0.001 | <0.001 | 0.84 | 0.63 | -0.21 |
| cg00026222 | 0.22 | 24.6 | <0.001 | <0.001 | 0.05 | 0.26 | 0.22 |
| cg21757951 | 0.25 | 24.5 | <0.001 | <0.001 | 0.11 | 0.37 | 0.25 |
| cg12534147 | 0.31 | 24.5 | <0.001 | <0.001 | 0.14 | 0.45 | 0.31 |
| cg19266671 | -0.26 | -24.5 | <0.001 | <0.001 | 0.81 | 0.56 | -0.26 |
| cg23167351 | 0.23 | 24.5 | <0.001 | <0.001 | 0.12 | 0.35 | 0.23 |
| cg09366730 | -0.20 | -24.5 | <0.001 | <0.001 | 0.87 | 0.67 | -0.20 |
| cg13568415 | -0.21 | -24.5 | <0.001 | <0.001 | 0.87 | 0.66 | -0.21 |
| cg19023977 | -0.28 | -24.5 | <0.001 | <0.001 | 0.80 | 0.52 | -0.28 |
| cg00870269 | -0.39 | -24.5 | <0.001 | <0.001 | 0.81 | 0.42 | -0.39 |
| cg11786776 | -0.28 | -24.5 | <0.001 | <0.001 | 0.82 | 0.55 | -0.28 |
| cg24823679 | -0.21 | -24.5 | <0.001 | <0.001 | 0.76 | 0.54 | -0.21 |
| cg26839512 | 0.36 | 24.4 | <0.001 | <0.001 | 0.20 | 0.55 | 0.36 |
| cg22222281 | -0.40 | -24.4 | <0.001 | <0.001 | 0.78 | 0.39 | -0.40 |
| cg22311403 | -0.22 | -24.4 | <0.001 | <0.001 | 0.79 | 0.56 | -0.22 |
| cg02458062 | -0.34 | -24.4 | <0.001 | <0.001 | 0.88 | 0.54 | -0.34 |
| cg18084609 | -0.26 | -24.4 | <0.001 | <0.001 | 0.79 | 0.53 | -0.26 |
| cg27285599 | -0.20 | -24.4 | <0.001 | <0.001 | 0.91 | 0.71 | -0.20 |
| cg02118390 | -0.25 | -24.4 | <0.001 | <0.001 | 0.93 | 0.68 | -0.25 |
| cg06606386 | 0.29 | 24.4 | <0.001 | <0.001 | 0.18 | 0.47 | 0.29 |
| cg24148817 | -0.34 | -24.4 | <0.001 | <0.001 | 0.81 | 0.46 | -0.34 |
| cg01819912 | -0.25 | -24.4 | <0.001 | <0.001 | 0.89 | 0.64 | -0.25 |
| cg08484337 | -0.23 | -24.3 | <0.001 | <0.001 | 0.87 | 0.65 | -0.23 |
| cg16616467 | -0.20 | -24.3 | <0.001 | <0.001 | 0.91 | 0.71 | -0.20 |
| cg00755063 | 0.26 | 24.3 | <0.001 | <0.001 | 0.17 | 0.42 | 0.26 |
| cg01066220 | -0.28 | -24.3 | <0.001 | <0.001 | 0.92 | 0.64 | -0.28 |
| cg07664000 | 0.22 | 24.3 | <0.001 | <0.001 | 0.17 | 0.40 | 0.22 |
| cg08470264 | -0.23 | -24.3 | <0.001 | <0.001 | 0.78 | 0.55 | -0.23 |
| cg00742738 | -0.25 | -24.3 | <0.001 | <0.001 | 0.89 | 0.63 | -0.25 |
| cg16304656 | 0.43 | 24.3 | <0.001 | <0.001 | 0.18 | 0.62 | 0.43 |
| cg13863668 | 0.27 | 24.3 | <0.001 | <0.001 | 0.16 | 0.43 | 0.27 |
| cg25489732 | -0.25 | -24.3 | <0.001 | <0.001 | 0.80 | 0.55 | -0.25 |
| cg07966265 | -0.21 | -24.3 | <0.001 | <0.001 | 0.89 | 0.67 | -0.21 |
| cg25745246 | -0.36 | -24.3 | <0.001 | <0.001 | 0.85 | 0.49 | -0.36 |
| cg07805542 | 0.29 | 24.2 | <0.001 | <0.001 | 0.17 | 0.46 | 0.29 |
| cg27000590 | -0.28 | -24.2 | <0.001 | <0.001 | 0.83 | 0.55 | -0.28 |
| cg10828599 | -0.21 | -24.2 | <0.001 | <0.001 | 0.92 | 0.71 | -0.21 |
| cg26214645 | -0.26 | -24.2 | <0.001 | <0.001 | 0.66 | 0.41 | -0.26 |
| cg00370123 | -0.29 | -24.2 | <0.001 | <0.001 | 0.75 | 0.46 | -0.29 |
| cg07249433 | -0.29 | -24.2 | <0.001 | <0.001 | 0.90 | 0.61 | -0.29 |
| cg07997333 | -0.33 | -24.1 | <0.001 | <0.001 | 0.76 | 0.43 | -0.33 |
| cg12922492 | 0.33 | 24.1 | <0.001 | <0.001 | 0.16 | 0.48 | 0.33 |
| cg19037350 | -0.20 | -24.1 | <0.001 | <0.001 | 0.60 | 0.40 | -0.20 |
| cg18954144 | -0.30 | -24.1 | <0.001 | <0.001 | 0.85 | 0.55 | -0.30 |
| cg20243900 | -0.27 | -24.1 | <0.001 | <0.001 | 0.87 | 0.59 | -0.27 |
| cg03223959 | -0.28 | -24.1 | <0.001 | <0.001 | 0.83 | 0.55 | -0.28 |
| cg16990945 | -0.34 | -24.1 | <0.001 | <0.001 | 0.86 | 0.53 | -0.34 |
| cg18054674 | -0.23 | -24.1 | <0.001 | <0.001 | 0.74 | 0.51 | -0.23 |
| cg08296191 | -0.22 | -24.1 | <0.001 | <0.001 | 0.86 | 0.63 | -0.22 |
| cg07671622 | -0.23 | -24.1 | <0.001 | <0.001 | 0.71 | 0.47 | -0.23 |
| cg11288144 | 0.40 | 24.1 | <0.001 | <0.001 | 0.19 | 0.59 | 0.40 |
| cg26111283 | -0.24 | -24.0 | <0.001 | <0.001 | 0.79 | 0.54 | -0.24 |
| cg00028336 | -0.24 | -24.0 | <0.001 | <0.001 | 0.83 | 0.60 | -0.24 |
| cg09636715 | -0.29 | -24.0 | <0.001 | <0.001 | 0.81 | 0.52 | -0.29 |
| cg03048372 | -0.36 | -24.0 | <0.001 | <0.001 | 0.84 | 0.49 | -0.36 |
| cg09548780 | 0.32 | 24.0 | <0.001 | <0.001 | 0.18 | 0.50 | 0.32 |
| cg16079541 | -0.22 | -24.0 | <0.001 | <0.001 | 0.79 | 0.57 | -0.22 |
| cg21005438 | -0.23 | -24.0 | <0.001 | <0.001 | 0.78 | 0.55 | -0.23 |
| cg25408314 | 0.32 | 24.0 | <0.001 | <0.001 | 0.16 | 0.48 | 0.32 |
| cg20810072 | -0.25 | -23.9 | <0.001 | <0.001 | 0.90 | 0.65 | -0.25 |
| cg18470456 | -0.29 | -23.9 | <0.001 | <0.001 | 0.90 | 0.60 | -0.29 |
| cg21594702 | 0.22 | 23.9 | <0.001 | <0.001 | 0.11 | 0.33 | 0.22 |
| cg01628067 | 0.24 | 23.9 | <0.001 | <0.001 | 0.20 | 0.44 | 0.24 |
| cg01903185 | -0.21 | -23.9 | <0.001 | <0.001 | 0.91 | 0.71 | -0.21 |
| cg13408597 | -0.25 | -23.9 | <0.001 | <0.001 | 0.91 | 0.66 | -0.25 |
| cg05407003 | -0.31 | -23.9 | <0.001 | <0.001 | 0.71 | 0.40 | -0.31 |
| cg22934449 | -0.20 | -23.9 | <0.001 | <0.001 | 0.94 | 0.74 | -0.20 |
| cg02762634 | -0.29 | -23.8 | <0.001 | <0.001 | 0.85 | 0.56 | -0.29 |
| cg01827195 | 0.24 | 23.8 | <0.001 | <0.001 | 0.17 | 0.42 | 0.24 |
| cg17783244 | -0.29 | -23.8 | <0.001 | <0.001 | 0.77 | 0.48 | -0.29 |
| cg23212751 | -0.32 | -23.8 | <0.001 | <0.001 | 0.74 | 0.42 | -0.32 |
| cg08605301 | -0.32 | -23.8 | <0.001 | <0.001 | 0.74 | 0.42 | -0.32 |
| cg22193276 | -0.23 | -23.8 | <0.001 | <0.001 | 0.81 | 0.58 | -0.23 |
| cg15049402 | -0.26 | -23.8 | <0.001 | <0.001 | 0.73 | 0.47 | -0.26 |
| cg14397893 | -0.24 | -23.8 | <0.001 | <0.001 | 0.81 | 0.57 | -0.24 |
| cg09313745 | -0.35 | -23.8 | <0.001 | <0.001 | 0.85 | 0.50 | -0.35 |
| cg01738022 | 0.40 | 23.8 | <0.001 | <0.001 | 0.19 | 0.59 | 0.40 |
| cg14042190 | -0.28 | -23.8 | <0.001 | <0.001 | 0.61 | 0.34 | -0.28 |
| cg02872914 | -0.26 | -23.8 | <0.001 | <0.001 | 0.86 | 0.60 | -0.26 |
| cg00607989 | -0.24 | -23.7 | <0.001 | <0.001 | 0.78 | 0.53 | -0.24 |
| cg16210447 | 0.26 | 23.7 | <0.001 | <0.001 | 0.11 | 0.37 | 0.26 |
| cg13318256 | -0.26 | -23.7 | <0.001 | <0.001 | 0.82 | 0.56 | -0.26 |
| cg07547765 | -0.33 | -23.7 | <0.001 | <0.001 | 0.83 | 0.50 | -0.33 |
| cg00423364 | -0.25 | -23.7 | <0.001 | <0.001 | 0.79 | 0.54 | -0.25 |
| cg10249734 | -0.29 | -23.7 | <0.001 | <0.001 | 0.69 | 0.40 | -0.29 |
| cg21163347 | -0.33 | -23.7 | <0.001 | <0.001 | 0.84 | 0.51 | -0.33 |
| cg05253480 | -0.29 | -23.7 | <0.001 | <0.001 | 0.77 | 0.48 | -0.29 |
| cg04844534 | -0.34 | -23.7 | <0.001 | <0.001 | 0.80 | 0.46 | -0.34 |
| cg12015737 | -0.32 | -23.7 | <0.001 | <0.001 | 0.86 | 0.54 | -0.32 |
| cg08858245 | -0.25 | -23.7 | <0.001 | <0.001 | 0.83 | 0.58 | -0.25 |
| cg19528338 | 0.21 | 23.6 | <0.001 | <0.001 | 0.20 | 0.41 | 0.21 |
| cg26065841 | -0.21 | -23.6 | <0.001 | <0.001 | 0.79 | 0.57 | -0.21 |
| cg10828284 | -0.32 | -23.6 | <0.001 | <0.001 | 0.61 | 0.29 | -0.32 |
| cg24122751 | -0.24 | -23.6 | <0.001 | <0.001 | 0.83 | 0.59 | -0.24 |
| cg22853713 | -0.31 | -23.6 | <0.001 | <0.001 | 0.89 | 0.57 | -0.31 |
| cg04487296 | -0.37 | -23.6 | <0.001 | <0.001 | 0.68 | 0.31 | -0.37 |
| cg20097219 | -0.26 | -23.6 | <0.001 | <0.001 | 0.71 | 0.45 | -0.26 |
| cg04918696 | -0.31 | -23.6 | <0.001 | <0.001 | 0.63 | 0.33 | -0.31 |
| cg20306574 | -0.21 | -23.6 | <0.001 | <0.001 | 0.90 | 0.69 | -0.21 |
| cg11516004 | -0.24 | -23.6 | <0.001 | <0.001 | 0.82 | 0.58 | -0.24 |
| cg07080372 | 0.21 | 23.5 | <0.001 | <0.001 | 0.15 | 0.36 | 0.21 |
| cg10548254 | -0.24 | -23.5 | <0.001 | <0.001 | 0.72 | 0.48 | -0.24 |
| cg19062108 | 0.24 | 23.5 | <0.001 | <0.001 | 0.16 | 0.40 | 0.24 |
| cg20732304 | -0.23 | -23.5 | <0.001 | <0.001 | 0.75 | 0.52 | -0.23 |
| cg20789483 | -0.30 | -23.5 | <0.001 | <0.001 | 0.77 | 0.47 | -0.30 |
| cg21722795 | -0.28 | -23.5 | <0.001 | <0.001 | 0.68 | 0.40 | -0.28 |
| cg01881549 | -0.25 | -23.5 | <0.001 | <0.001 | 0.78 | 0.53 | -0.25 |
| cg27500983 | -0.26 | -23.5 | <0.001 | <0.001 | 0.83 | 0.57 | -0.26 |
| cg02365900 | -0.22 | -23.5 | <0.001 | <0.001 | 0.86 | 0.64 | -0.22 |
| cg02298094 | 0.32 | 23.4 | <0.001 | <0.001 | 0.19 | 0.51 | 0.32 |
| cg27320005 | -0.22 | -23.4 | <0.001 | <0.001 | 0.83 | 0.61 | -0.22 |
| cg25317233 | -0.23 | -23.4 | <0.001 | <0.001 | 0.84 | 0.62 | -0.23 |
| cg09507567 | 0.24 | 23.4 | <0.001 | <0.001 | 0.18 | 0.42 | 0.24 |
| cg11116288 | -0.28 | -23.4 | <0.001 | <0.001 | 0.87 | 0.59 | -0.28 |
| cg04191989 | -0.28 | -23.4 | <0.001 | <0.001 | 0.89 | 0.61 | -0.28 |
| cg02501155 | -0.36 | -23.4 | <0.001 | <0.001 | 0.85 | 0.49 | -0.36 |
| cg16719560 | 0.23 | 23.4 | <0.001 | <0.001 | 0.09 | 0.33 | 0.23 |
| cg05487589 | 0.21 | 23.4 | <0.001 | <0.001 | 0.17 | 0.37 | 0.21 |
| cg26481727 | -0.31 | -23.4 | <0.001 | <0.001 | 0.68 | 0.37 | -0.31 |
| cg27252766 | -0.23 | -23.4 | <0.001 | <0.001 | 0.86 | 0.63 | -0.23 |
| cg01399485 | -0.22 | -23.4 | <0.001 | <0.001 | 0.78 | 0.56 | -0.22 |
| cg09748975 | -0.25 | -23.4 | <0.001 | <0.001 | 0.79 | 0.55 | -0.25 |
| cg06665333 | -0.27 | -23.4 | <0.001 | <0.001 | 0.86 | 0.59 | -0.27 |
| cg04330597 | -0.28 | -23.3 | <0.001 | <0.001 | 0.88 | 0.60 | -0.28 |
| cg03070236 | 0.42 | 23.3 | <0.001 | <0.001 | 0.20 | 0.62 | 0.42 |
| cg04522310 | 0.34 | 23.3 | <0.001 | <0.001 | 0.11 | 0.45 | 0.34 |
| cg04885068 | -0.21 | -23.3 | <0.001 | <0.001 | 0.82 | 0.61 | -0.21 |
| cg05573550 | -0.21 | -23.3 | <0.001 | <0.001 | 0.66 | 0.45 | -0.21 |
| cg03928961 | -0.34 | -23.3 | <0.001 | <0.001 | 0.83 | 0.49 | -0.34 |
| cg08630080 | -0.21 | -23.3 | <0.001 | <0.001 | 0.90 | 0.69 | -0.21 |
| cg05989861 | -0.27 | -23.3 | <0.001 | <0.001 | 0.85 | 0.58 | -0.27 |
| cg13823144 | -0.20 | -23.3 | <0.001 | <0.001 | 0.94 | 0.74 | -0.20 |
| cg12209075 | 0.22 | 23.3 | <0.001 | <0.001 | 0.15 | 0.38 | 0.22 |
| cg19940077 | -0.25 | -23.3 | <0.001 | <0.001 | 0.78 | 0.53 | -0.25 |
| cg21097354 | -0.36 | -23.2 | <0.001 | <0.001 | 0.73 | 0.38 | -0.36 |
| cg26767081 | 0.23 | 23.2 | <0.001 | <0.001 | 0.14 | 0.37 | 0.23 |
| cg16787431 | -0.31 | -23.2 | <0.001 | <0.001 | 0.90 | 0.59 | -0.31 |
| cg09464206 | 0.27 | 23.2 | <0.001 | <0.001 | 0.19 | 0.46 | 0.27 |
| cg16805189 | -0.27 | -23.2 | <0.001 | <0.001 | 0.82 | 0.55 | -0.27 |
| cg17565967 | -0.23 | -23.2 | <0.001 | <0.001 | 0.75 | 0.52 | -0.23 |
| cg11085454 | -0.22 | -23.2 | <0.001 | <0.001 | 0.62 | 0.40 | -0.22 |
| cg16732787 | -0.26 | -23.2 | <0.001 | <0.001 | 0.61 | 0.35 | -0.26 |
| cg20821442 | -0.29 | -23.2 | <0.001 | <0.001 | 0.87 | 0.58 | -0.29 |
| cg01025690 | -0.22 | -23.2 | <0.001 | <0.001 | 0.71 | 0.49 | -0.22 |
| cg01977762 | -0.39 | -23.2 | <0.001 | <0.001 | 0.68 | 0.29 | -0.39 |
| cg17103638 | -0.21 | -23.2 | <0.001 | <0.001 | 0.89 | 0.67 | -0.21 |
| cg19857714 | -0.23 | -23.1 | <0.001 | <0.001 | 0.65 | 0.42 | -0.23 |
| cg01171786 | -0.22 | -23.1 | <0.001 | <0.001 | 0.89 | 0.67 | -0.22 |
| cg18630178 | -0.22 | -23.1 | <0.001 | <0.001 | 0.68 | 0.46 | -0.22 |
| cg13683667 | -0.30 | -23.1 | <0.001 | <0.001 | 0.83 | 0.53 | -0.30 |
| cg08954601 | -0.29 | -23.1 | <0.001 | <0.001 | 0.84 | 0.55 | -0.29 |
| cg25564800 | 0.22 | 23.1 | <0.001 | <0.001 | 0.15 | 0.37 | 0.22 |
| cg18173263 | -0.25 | -23.1 | <0.001 | <0.001 | 0.73 | 0.48 | -0.25 |
| cg08549335 | 0.26 | 23.1 | <0.001 | <0.001 | 0.19 | 0.46 | 0.26 |
| cg08264122 | -0.24 | -23.1 | <0.001 | <0.001 | 0.89 | 0.65 | -0.24 |
| cg04204002 | -0.26 | -23.1 | <0.001 | <0.001 | 0.87 | 0.61 | -0.26 |
| cg05666820 | -0.26 | -23.0 | <0.001 | <0.001 | 0.77 | 0.51 | -0.26 |
| cg01083652 | -0.30 | -23.0 | <0.001 | <0.001 | 0.79 | 0.49 | -0.30 |
| cg07039560 | -0.25 | -23.0 | <0.001 | <0.001 | 0.65 | 0.40 | -0.25 |
| cg04221877 | -0.30 | -23.0 | <0.001 | <0.001 | 0.66 | 0.36 | -0.30 |
| cg26620655 | -0.33 | -23.0 | <0.001 | <0.001 | 0.85 | 0.52 | -0.33 |
| cg06297571 | -0.25 | -23.0 | <0.001 | <0.001 | 0.92 | 0.67 | -0.25 |
| cg06784539 | -0.28 | -22.9 | <0.001 | <0.001 | 0.62 | 0.34 | -0.28 |
| cg09329516 | -0.23 | -22.9 | <0.001 | <0.001 | 0.80 | 0.57 | -0.23 |
| cg12859429 | -0.24 | -22.9 | <0.001 | <0.001 | 0.75 | 0.52 | -0.24 |
| cg04122918 | -0.22 | -22.9 | <0.001 | <0.001 | 0.81 | 0.59 | -0.22 |
| cg23972735 | -0.32 | -22.9 | <0.001 | <0.001 | 0.71 | 0.39 | -0.32 |
| cg17295878 | -0.21 | -22.9 | <0.001 | <0.001 | 0.91 | 0.70 | -0.21 |
| cg07371589 | -0.37 | -22.8 | <0.001 | <0.001 | 0.72 | 0.35 | -0.37 |
| cg27454102 | 0.21 | 22.8 | <0.001 | <0.001 | 0.11 | 0.32 | 0.21 |
| cg11230435 | 0.20 | 22.8 | <0.001 | <0.001 | 0.18 | 0.38 | 0.20 |
| cg19505659 | -0.23 | -22.8 | <0.001 | <0.001 | 0.72 | 0.48 | -0.23 |
| cg23551494 | 0.30 | 22.8 | <0.001 | <0.001 | 0.16 | 0.46 | 0.30 |
| cg13007207 | -0.25 | -22.8 | <0.001 | <0.001 | 0.88 | 0.63 | -0.25 |
| cg02744046 | -0.31 | -22.8 | <0.001 | <0.001 | 0.73 | 0.42 | -0.31 |
| cg12399484 | -0.22 | -22.8 | <0.001 | <0.001 | 0.89 | 0.67 | -0.22 |
| cg00809969 | -0.26 | -22.8 | <0.001 | <0.001 | 0.79 | 0.53 | -0.26 |
| cg04972436 | -0.34 | -22.7 | <0.001 | <0.001 | 0.70 | 0.36 | -0.34 |
| cg24280832 | -0.35 | -22.7 | <0.001 | <0.001 | 0.90 | 0.55 | -0.35 |
| cg11183144 | -0.22 | -22.7 | <0.001 | <0.001 | 0.79 | 0.58 | -0.22 |
| cg26869615 | -0.33 | -22.7 | <0.001 | <0.001 | 0.85 | 0.52 | -0.33 |
| cg07828377 | -0.21 | -22.7 | <0.001 | <0.001 | 0.79 | 0.58 | -0.21 |
| cg09937500 | -0.31 | -22.7 | <0.001 | <0.001 | 0.77 | 0.45 | -0.31 |
| cg07429146 | -0.27 | -22.7 | <0.001 | <0.001 | 0.72 | 0.45 | -0.27 |
| cg21055554 | 0.23 | 22.7 | <0.001 | <0.001 | 0.13 | 0.35 | 0.23 |
| cg21538208 | -0.37 | -22.7 | <0.001 | <0.001 | 0.64 | 0.27 | -0.37 |
| cg06650260 | -0.28 | -22.7 | <0.001 | <0.001 | 0.82 | 0.54 | -0.28 |
| cg18305324 | -0.24 | -22.7 | <0.001 | <0.001 | 0.70 | 0.46 | -0.24 |
| cg07635227 | 0.37 | 22.7 | <0.001 | <0.001 | 0.13 | 0.50 | 0.37 |
| cg25977879 | -0.23 | -22.7 | <0.001 | <0.001 | 0.83 | 0.60 | -0.23 |
| cg11601197 | -0.22 | -22.7 | <0.001 | <0.001 | 0.84 | 0.62 | -0.22 |
| cg20834178 | 0.30 | 22.7 | <0.001 | <0.001 | 0.18 | 0.48 | 0.30 |
| cg08528170 | 0.20 | 22.7 | <0.001 | <0.001 | 0.09 | 0.30 | 0.20 |
| cg27391627 | -0.39 | -22.7 | <0.001 | <0.001 | 0.75 | 0.36 | -0.39 |
| cg08124986 | 0.20 | 22.7 | <0.001 | <0.001 | 0.18 | 0.39 | 0.20 |
| cg12538674 | -0.27 | -22.7 | <0.001 | <0.001 | 0.72 | 0.45 | -0.27 |
| cg16223546 | 0.24 | 22.6 | <0.001 | <0.001 | 0.14 | 0.38 | 0.24 |
| cg03762081 | 0.25 | 22.6 | <0.001 | <0.001 | 0.16 | 0.40 | 0.25 |
| cg20912978 | 0.22 | 22.6 | <0.001 | <0.001 | 0.19 | 0.41 | 0.22 |
| cg13290149 | -0.23 | -22.6 | <0.001 | <0.001 | 0.69 | 0.45 | -0.23 |
| cg08896030 | -0.24 | -22.6 | <0.001 | <0.001 | 0.82 | 0.58 | -0.24 |
| cg01574481 | -0.25 | -22.6 | <0.001 | <0.001 | 0.67 | 0.43 | -0.25 |
| cg24336398 | -0.22 | -22.6 | <0.001 | <0.001 | 0.69 | 0.47 | -0.22 |
| cg02669225 | -0.25 | -22.6 | <0.001 | <0.001 | 0.88 | 0.62 | -0.25 |
| cg02371119 | 0.23 | 22.6 | <0.001 | <0.001 | 0.11 | 0.34 | 0.23 |
| cg11222217 | -0.32 | -22.6 | <0.001 | <0.001 | 0.66 | 0.34 | -0.32 |
| cg11385338 | -0.33 | -22.6 | <0.001 | <0.001 | 0.71 | 0.38 | -0.33 |
| cg26697310 | -0.25 | -22.5 | <0.001 | <0.001 | 0.81 | 0.56 | -0.25 |
| cg04880737 | -0.33 | -22.5 | <0.001 | <0.001 | 0.69 | 0.36 | -0.33 |
| cg19542849 | -0.31 | -22.5 | <0.001 | <0.001 | 0.65 | 0.34 | -0.31 |
| cg20187173 | -0.31 | -22.5 | <0.001 | <0.001 | 0.80 | 0.48 | -0.31 |
| cg00927435 | -0.22 | -22.5 | <0.001 | <0.001 | 0.85 | 0.63 | -0.22 |
| cg26534677 | 0.21 | 22.5 | <0.001 | <0.001 | 0.18 | 0.39 | 0.21 |
| cg00253658 | -0.41 | -22.5 | <0.001 | <0.001 | 0.63 | 0.22 | -0.41 |
| cg16888590 | -0.29 | -22.5 | <0.001 | <0.001 | 0.86 | 0.57 | -0.29 |
| cg08124209 | -0.23 | -22.5 | <0.001 | <0.001 | 0.78 | 0.55 | -0.23 |
| cg22611850 | -0.22 | -22.5 | <0.001 | <0.001 | 0.87 | 0.65 | -0.22 |
| cg18351999 | 0.29 | 22.5 | <0.001 | <0.001 | 0.20 | 0.49 | 0.29 |
| cg26622699 | -0.27 | -22.5 | <0.001 | <0.001 | 0.80 | 0.53 | -0.27 |
| cg05280762 | -0.32 | -22.4 | <0.001 | <0.001 | 0.69 | 0.37 | -0.32 |
| cg02941741 | -0.28 | -22.4 | <0.001 | <0.001 | 0.76 | 0.47 | -0.28 |
| cg13725657 | -0.26 | -22.4 | <0.001 | <0.001 | 0.82 | 0.57 | -0.26 |
| cg00852033 | 0.21 | 22.4 | <0.001 | <0.001 | 0.15 | 0.36 | 0.21 |
| cg01107874 | -0.30 | -22.4 | <0.001 | <0.001 | 0.80 | 0.50 | -0.30 |
| cg10996039 | -0.28 | -22.4 | <0.001 | <0.001 | 0.76 | 0.48 | -0.28 |
| cg02436098 | 0.26 | 22.4 | <0.001 | <0.001 | 0.17 | 0.43 | 0.26 |
| cg25316172 | -0.29 | -22.4 | <0.001 | <0.001 | 0.77 | 0.48 | -0.29 |
| cg10453419 | 0.32 | 22.4 | <0.001 | <0.001 | 0.15 | 0.47 | 0.32 |
| cg24767968 | -0.33 | -22.4 | <0.001 | <0.001 | 0.79 | 0.46 | -0.33 |
| cg13694927 | -0.22 | -22.4 | <0.001 | <0.001 | 0.71 | 0.49 | -0.22 |
| cg03899412 | -0.24 | -22.4 | <0.001 | <0.001 | 0.86 | 0.62 | -0.24 |
| cg27319188 | 0.25 | 22.4 | <0.001 | <0.001 | 0.19 | 0.44 | 0.25 |
| cg26047066 | -0.38 | -22.4 | <0.001 | <0.001 | 0.70 | 0.32 | -0.38 |
| cg00711072 | -0.35 | -22.4 | <0.001 | <0.001 | 0.83 | 0.48 | -0.35 |
| cg00941576 | -0.24 | -22.4 | <0.001 | <0.001 | 0.75 | 0.52 | -0.24 |
| cg03129324 | 0.20 | 22.3 | <0.001 | <0.001 | 0.17 | 0.38 | 0.20 |
| cg03099728 | -0.20 | -22.3 | <0.001 | <0.001 | 0.73 | 0.52 | -0.20 |
| cg16146718 | -0.23 | -22.3 | <0.001 | <0.001 | 0.94 | 0.71 | -0.23 |
| cg26819718 | -0.27 | -22.3 | <0.001 | <0.001 | 0.92 | 0.65 | -0.27 |
| cg01977473 | -0.26 | -22.3 | <0.001 | <0.001 | 0.87 | 0.61 | -0.26 |
| cg11380483 | -0.31 | -22.3 | <0.001 | <0.001 | 0.76 | 0.44 | -0.31 |
| cg08699206 | -0.34 | -22.3 | <0.001 | <0.001 | 0.75 | 0.40 | -0.34 |
| cg14327759 | -0.27 | -22.3 | <0.001 | <0.001 | 0.89 | 0.61 | -0.27 |
| cg09933323 | -0.32 | -22.2 | <0.001 | <0.001 | 0.65 | 0.33 | -0.32 |
| cg16209517 | -0.28 | -22.2 | <0.001 | <0.001 | 0.79 | 0.52 | -0.28 |
| cg01541650 | -0.20 | -22.2 | <0.001 | <0.001 | 0.91 | 0.71 | -0.20 |
| cg24699296 | -0.29 | -22.2 | <0.001 | <0.001 | 0.75 | 0.45 | -0.29 |
| cg16547186 | 0.26 | 22.2 | <0.001 | <0.001 | 0.15 | 0.41 | 0.26 |
| cg00515954 | -0.35 | -22.2 | <0.001 | <0.001 | 0.75 | 0.40 | -0.35 |
| cg00411072 | -0.24 | -22.2 | <0.001 | <0.001 | 0.88 | 0.64 | -0.24 |
| cg15300753 | -0.22 | -22.2 | <0.001 | <0.001 | 0.80 | 0.59 | -0.22 |
| cg02774334 | -0.23 | -22.2 | <0.001 | <0.001 | 0.83 | 0.61 | -0.23 |
| cg01961752 | 0.26 | 22.2 | <0.001 | <0.001 | 0.14 | 0.40 | 0.26 |
| cg10362613 | -0.25 | -22.2 | <0.001 | <0.001 | 0.87 | 0.62 | -0.25 |
| cg16479247 | -0.26 | -22.1 | <0.001 | <0.001 | 0.88 | 0.62 | -0.26 |
| cg09975219 | -0.22 | -22.1 | <0.001 | <0.001 | 0.90 | 0.68 | -0.22 |
| cg00891541 | -0.34 | -22.1 | <0.001 | <0.001 | 0.64 | 0.31 | -0.34 |
| cg27624327 | -0.36 | -22.1 | <0.001 | <0.001 | 0.78 | 0.43 | -0.36 |
| cg26349773 | -0.26 | -22.1 | <0.001 | <0.001 | 0.80 | 0.54 | -0.26 |
| cg14354168 | -0.21 | -22.1 | <0.001 | <0.001 | 0.88 | 0.67 | -0.21 |
| cg02961385 | -0.25 | -22.1 | <0.001 | <0.001 | 0.94 | 0.69 | -0.25 |
| cg18127922 | -0.22 | -22.1 | <0.001 | <0.001 | 0.85 | 0.62 | -0.22 |
| cg07676709 | -0.25 | -22.1 | <0.001 | <0.001 | 0.64 | 0.39 | -0.25 |
| cg12410980 | -0.24 | -22.1 | <0.001 | <0.001 | 0.80 | 0.56 | -0.24 |
| cg14633892 | -0.29 | -22.1 | <0.001 | <0.001 | 0.77 | 0.48 | -0.29 |
| cg27468976 | -0.33 | -22.1 | <0.001 | <0.001 | 0.78 | 0.45 | -0.33 |
| cg02384546 | -0.23 | -22.1 | <0.001 | <0.001 | 0.85 | 0.62 | -0.23 |
| cg17831791 | -0.23 | -22.1 | <0.001 | <0.001 | 0.79 | 0.55 | -0.23 |
| cg17161048 | -0.26 | -22.1 | <0.001 | <0.001 | 0.75 | 0.49 | -0.26 |
| cg07496545 | -0.34 | -22.1 | <0.001 | <0.001 | 0.82 | 0.48 | -0.34 |
| cg01980810 | -0.20 | -22.0 | <0.001 | <0.001 | 0.86 | 0.66 | -0.20 |
| cg16271437 | 0.23 | 22.0 | <0.001 | <0.001 | 0.13 | 0.36 | 0.23 |
| cg02589501 | -0.25 | -22.0 | <0.001 | <0.001 | 0.76 | 0.51 | -0.25 |
| cg23821914 | -0.24 | -22.0 | <0.001 | <0.001 | 0.83 | 0.59 | -0.24 |
| cg18081338 | -0.25 | -22.0 | <0.001 | <0.001 | 0.78 | 0.53 | -0.25 |
| cg23942526 | -0.34 | -22.0 | <0.001 | <0.001 | 0.83 | 0.49 | -0.34 |
| cg01244934 | -0.26 | -22.0 | <0.001 | <0.001 | 0.75 | 0.49 | -0.26 |
| cg22453113 | 0.28 | 22.0 | <0.001 | <0.001 | 0.09 | 0.36 | 0.28 |
| cg02109605 | -0.29 | -22.0 | <0.001 | <0.001 | 0.74 | 0.46 | -0.29 |
| cg19848599 | -0.29 | -22.0 | <0.001 | <0.001 | 0.88 | 0.60 | -0.29 |
| cg06787991 | -0.24 | -22.0 | <0.001 | <0.001 | 0.91 | 0.67 | -0.24 |
| cg02698576 | -0.25 | -22.0 | <0.001 | <0.001 | 0.83 | 0.57 | -0.25 |
| cg06024930 | -0.24 | -22.0 | <0.001 | <0.001 | 0.78 | 0.54 | -0.24 |
| cg00852595 | -0.35 | -22.0 | <0.001 | <0.001 | 0.73 | 0.38 | -0.35 |
| cg10289744 | -0.33 | -21.9 | <0.001 | <0.001 | 0.78 | 0.45 | -0.33 |
| cg04800503 | -0.28 | -21.9 | <0.001 | <0.001 | 0.89 | 0.60 | -0.28 |
| cg26204477 | -0.20 | -21.9 | <0.001 | <0.001 | 0.82 | 0.62 | -0.20 |
| cg15320905 | -0.40 | -21.9 | <0.001 | <0.001 | 0.80 | 0.40 | -0.40 |
| cg27625456 | 0.32 | 21.9 | <0.001 | <0.001 | 0.16 | 0.48 | 0.32 |
| cg04544533 | -0.22 | -21.9 | <0.001 | <0.001 | 0.82 | 0.61 | -0.22 |
| cg23205676 | -0.21 | -21.9 | <0.001 | <0.001 | 0.85 | 0.64 | -0.21 |
| cg24380053 | -0.26 | -21.9 | <0.001 | <0.001 | 0.82 | 0.56 | -0.26 |
| cg24183575 | -0.24 | -21.9 | <0.001 | <0.001 | 0.80 | 0.56 | -0.24 |
| cg11046030 | -0.26 | -21.9 | <0.001 | <0.001 | 0.82 | 0.55 | -0.26 |
| cg00804078 | 0.20 | 21.9 | <0.001 | <0.001 | 0.15 | 0.35 | 0.20 |
| cg12744859 | -0.37 | -21.9 | <0.001 | <0.001 | 0.78 | 0.41 | -0.37 |
| cg01696784 | -0.35 | -21.9 | <0.001 | <0.001 | 0.88 | 0.53 | -0.35 |
| cg05796704 | -0.32 | -21.9 | <0.001 | <0.001 | 0.73 | 0.42 | -0.32 |
| cg26336701 | -0.26 | -21.8 | <0.001 | <0.001 | 0.91 | 0.65 | -0.26 |
| cg16768376 | -0.25 | -21.8 | <0.001 | <0.001 | 0.83 | 0.58 | -0.25 |
| cg02565132 | -0.21 | -21.8 | <0.001 | <0.001 | 0.88 | 0.67 | -0.21 |
| cg21653641 | -0.34 | -21.8 | <0.001 | <0.001 | 0.68 | 0.34 | -0.34 |
| cg11956467 | -0.20 | -21.8 | <0.001 | <0.001 | 0.85 | 0.65 | -0.20 |
| cg04117801 | -0.36 | -21.8 | <0.001 | <0.001 | 0.87 | 0.52 | -0.36 |
| cg04407490 | -0.23 | -21.8 | <0.001 | <0.001 | 0.69 | 0.46 | -0.23 |
| cg24995083 | -0.31 | -21.8 | <0.001 | <0.001 | 0.72 | 0.42 | -0.31 |
| cg06800840 | 0.21 | 21.8 | <0.001 | <0.001 | 0.12 | 0.34 | 0.21 |
| cg04111078 | -0.25 | -21.8 | <0.001 | <0.001 | 0.94 | 0.69 | -0.25 |
| cg11429044 | 0.27 | 21.8 | <0.001 | <0.001 | 0.16 | 0.43 | 0.27 |
| cg21915313 | -0.28 | -21.8 | <0.001 | <0.001 | 0.71 | 0.44 | -0.28 |
| cg18073906 | -0.23 | -21.7 | <0.001 | <0.001 | 0.66 | 0.44 | -0.23 |
| cg03880611 | -0.27 | -21.7 | <0.001 | <0.001 | 0.77 | 0.50 | -0.27 |
| cg12453504 | -0.28 | -21.7 | <0.001 | <0.001 | 0.81 | 0.52 | -0.28 |
| cg08794954 | 0.24 | 21.7 | <0.001 | <0.001 | 0.14 | 0.38 | 0.24 |
| cg06615676 | -0.22 | -21.7 | <0.001 | <0.001 | 0.76 | 0.54 | -0.22 |
| cg15813266 | -0.23 | -21.7 | <0.001 | <0.001 | 0.88 | 0.65 | -0.23 |
| cg13192938 | -0.22 | -21.7 | <0.001 | <0.001 | 0.80 | 0.58 | -0.22 |
| cg16923137 | -0.23 | -21.7 | <0.001 | <0.001 | 0.87 | 0.64 | -0.23 |
| cg05991442 | -0.29 | -21.7 | <0.001 | <0.001 | 0.95 | 0.66 | -0.29 |
| cg01586116 | 0.29 | 21.7 | <0.001 | <0.001 | 0.13 | 0.43 | 0.29 |
| cg27390009 | -0.21 | -21.7 | <0.001 | <0.001 | 0.72 | 0.51 | -0.21 |
| cg19731612 | -0.24 | -21.7 | <0.001 | <0.001 | 0.77 | 0.52 | -0.24 |
| cg21541083 | -0.20 | -21.7 | <0.001 | <0.001 | 0.80 | 0.60 | -0.20 |
| cg10911287 | -0.25 | -21.6 | <0.001 | <0.001 | 0.62 | 0.37 | -0.25 |
| cg05154546 | -0.26 | -21.6 | <0.001 | <0.001 | 0.85 | 0.59 | -0.26 |
| cg22720392 | -0.34 | -21.6 | <0.001 | <0.001 | 0.78 | 0.44 | -0.34 |
| cg16662451 | -0.29 | -21.6 | <0.001 | <0.001 | 0.78 | 0.48 | -0.29 |
| cg08709360 | -0.27 | -21.6 | <0.001 | <0.001 | 0.86 | 0.60 | -0.27 |
| cg18645642 | -0.32 | -21.6 | <0.001 | <0.001 | 0.78 | 0.45 | -0.32 |
| cg07717903 | -0.25 | -21.6 | <0.001 | <0.001 | 0.68 | 0.42 | -0.25 |
| cg04991639 | 0.21 | 21.6 | <0.001 | <0.001 | 0.17 | 0.38 | 0.21 |
| cg11360546 | -0.31 | -21.6 | <0.001 | <0.001 | 0.70 | 0.39 | -0.31 |
| cg04359418 | -0.30 | -21.6 | <0.001 | <0.001 | 0.63 | 0.33 | -0.30 |
| cg22796923 | -0.29 | -21.6 | <0.001 | <0.001 | 0.76 | 0.47 | -0.29 |
| cg13064897 | -0.28 | -21.6 | <0.001 | <0.001 | 0.85 | 0.57 | -0.28 |
| cg07048519 | -0.30 | -21.6 | <0.001 | <0.001 | 0.77 | 0.48 | -0.30 |
| cg00128386 | -0.25 | -21.5 | <0.001 | <0.001 | 0.82 | 0.57 | -0.25 |
| cg09832911 | -0.29 | -21.5 | <0.001 | <0.001 | 0.70 | 0.41 | -0.29 |
| cg13281248 | -0.21 | -21.5 | <0.001 | <0.001 | 0.78 | 0.57 | -0.21 |
| cg02104112 | -0.26 | -21.5 | <0.001 | <0.001 | 0.82 | 0.56 | -0.26 |
| cg01132579 | -0.25 | -21.5 | <0.001 | <0.001 | 0.85 | 0.60 | -0.25 |
| cg14155027 | -0.30 | -21.5 | <0.001 | <0.001 | 0.80 | 0.50 | -0.30 |
| cg20817131 | -0.28 | -21.5 | <0.001 | <0.001 | 0.71 | 0.44 | -0.28 |
| cg16513459 | -0.21 | -21.5 | <0.001 | <0.001 | 0.75 | 0.54 | -0.21 |
| cg05700616 | -0.20 | -21.5 | <0.001 | <0.001 | 0.86 | 0.66 | -0.20 |
| cg17158913 | -0.25 | -21.5 | <0.001 | <0.001 | 0.72 | 0.47 | -0.25 |
| cg02455346 | -0.20 | -21.5 | <0.001 | <0.001 | 0.79 | 0.58 | -0.20 |
| cg26224624 | -0.28 | -21.5 | <0.001 | <0.001 | 0.76 | 0.48 | -0.28 |
| cg14909614 | -0.22 | -21.5 | <0.001 | <0.001 | 0.69 | 0.47 | -0.22 |
| cg16518772 | 0.22 | 21.4 | <0.001 | <0.001 | 0.10 | 0.32 | 0.22 |
| cg20949700 | -0.30 | -21.4 | <0.001 | <0.001 | 0.72 | 0.42 | -0.30 |
| cg04299200 | -0.33 | -21.4 | <0.001 | <0.001 | 0.83 | 0.50 | -0.33 |
| cg23743911 | -0.28 | -21.4 | <0.001 | <0.001 | 0.62 | 0.34 | -0.28 |
| cg21157465 | -0.24 | -21.4 | <0.001 | <0.001 | 0.85 | 0.61 | -0.24 |
| cg00601836 | -0.33 | -21.4 | <0.001 | <0.001 | 0.75 | 0.42 | -0.33 |
| cg01888044 | -0.20 | -21.4 | <0.001 | <0.001 | 0.97 | 0.77 | -0.20 |
| cg17147589 | -0.25 | -21.4 | <0.001 | <0.001 | 0.77 | 0.52 | -0.25 |
| cg07813265 | -0.29 | -21.4 | <0.001 | <0.001 | 0.70 | 0.41 | -0.29 |
| cg17029237 | -0.24 | -21.4 | <0.001 | <0.001 | 0.84 | 0.60 | -0.24 |
| cg18064754 | -0.20 | -21.4 | <0.001 | <0.001 | 0.93 | 0.73 | -0.20 |
| cg05324273 | -0.21 | -21.4 | <0.001 | <0.001 | 0.71 | 0.51 | -0.21 |
| cg22805381 | 0.24 | 21.3 | <0.001 | <0.001 | 0.18 | 0.41 | 0.24 |
| cg04968532 | -0.25 | -21.3 | <0.001 | <0.001 | 0.77 | 0.52 | -0.25 |
| cg01860693 | -0.22 | -21.3 | <0.001 | <0.001 | 0.81 | 0.59 | -0.22 |
| cg05138040 | -0.21 | -21.3 | <0.001 | <0.001 | 0.76 | 0.54 | -0.21 |
| cg13003786 | -0.22 | -21.3 | <0.001 | <0.001 | 0.80 | 0.57 | -0.22 |
| cg16915863 | -0.22 | -21.3 | <0.001 | <0.001 | 0.91 | 0.69 | -0.22 |
| cg14800014 | -0.25 | -21.3 | <0.001 | <0.001 | 0.90 | 0.65 | -0.25 |
| cg05704955 | -0.22 | -21.3 | <0.001 | <0.001 | 0.68 | 0.46 | -0.22 |
| cg13138089 | -0.27 | -21.3 | <0.001 | <0.001 | 0.64 | 0.37 | -0.27 |
| cg27582563 | -0.20 | -21.3 | <0.001 | <0.001 | 0.84 | 0.64 | -0.20 |
| cg10033433 | -0.29 | -21.2 | <0.001 | <0.001 | 0.81 | 0.53 | -0.29 |
| cg08266202 | -0.25 | -21.2 | <0.001 | <0.001 | 0.69 | 0.44 | -0.25 |
| cg04221461 | -0.23 | -21.2 | <0.001 | <0.001 | 0.70 | 0.46 | -0.23 |
| cg09571376 | -0.29 | -21.2 | <0.001 | <0.001 | 0.86 | 0.57 | -0.29 |
| cg16705777 | -0.23 | -21.2 | <0.001 | <0.001 | 0.87 | 0.64 | -0.23 |
| cg05590233 | -0.26 | -21.2 | <0.001 | <0.001 | 0.88 | 0.62 | -0.26 |
| cg01493517 | 0.24 | 21.2 | <0.001 | <0.001 | 0.13 | 0.37 | 0.24 |
| cg13399773 | -0.28 | -21.2 | <0.001 | <0.001 | 0.85 | 0.58 | -0.28 |
| cg00492070 | -0.24 | -21.2 | <0.001 | <0.001 | 0.84 | 0.59 | -0.24 |
| cg01307684 | -0.26 | -21.2 | <0.001 | <0.001 | 0.91 | 0.65 | -0.26 |
| cg02866106 | -0.28 | -21.2 | <0.001 | <0.001 | 0.67 | 0.39 | -0.28 |
| cg00716257 | -0.34 | -21.2 | <0.001 | <0.001 | 0.63 | 0.29 | -0.34 |
| cg02954562 | 0.21 | 21.2 | <0.001 | <0.001 | 0.12 | 0.33 | 0.21 |
| cg18623355 | -0.28 | -21.1 | <0.001 | <0.001 | 0.84 | 0.56 | -0.28 |
| cg20277126 | -0.25 | -21.1 | <0.001 | <0.001 | 0.71 | 0.46 | -0.25 |
| cg21116087 | -0.28 | -21.1 | <0.001 | <0.001 | 0.77 | 0.49 | -0.28 |
| cg00997998 | -0.27 | -21.1 | <0.001 | <0.001 | 0.74 | 0.47 | -0.27 |
| cg04569615 | -0.31 | -21.1 | <0.001 | <0.001 | 0.63 | 0.32 | -0.31 |
| cg00939727 | -0.23 | -21.1 | <0.001 | <0.001 | 0.89 | 0.66 | -0.23 |
| cg18074184 | -0.22 | -21.1 | <0.001 | <0.001 | 0.69 | 0.47 | -0.22 |
| cg02101355 | -0.23 | -21.1 | <0.001 | <0.001 | 0.79 | 0.55 | -0.23 |
| cg14276379 | 0.22 | 21.1 | <0.001 | <0.001 | 0.17 | 0.39 | 0.22 |
| cg04800142 | -0.22 | -21.1 | <0.001 | <0.001 | 0.87 | 0.65 | -0.22 |
| cg16924776 | -0.25 | -21.1 | <0.001 | <0.001 | 0.71 | 0.46 | -0.25 |
| cg26817546 | -0.22 | -21.1 | <0.001 | <0.001 | 0.68 | 0.45 | -0.22 |
| cg04340203 | -0.22 | -21.1 | <0.001 | <0.001 | 0.78 | 0.56 | -0.22 |
| cg09959355 | -0.21 | -21.1 | <0.001 | <0.001 | 0.90 | 0.69 | -0.21 |
| cg02639181 | -0.32 | -21.1 | <0.001 | <0.001 | 0.76 | 0.44 | -0.32 |
| cg03555299 | -0.23 | -21.1 | <0.001 | <0.001 | 0.90 | 0.67 | -0.23 |
| cg15122993 | -0.23 | -21.1 | <0.001 | <0.001 | 0.86 | 0.63 | -0.23 |
| cg04798016 | -0.25 | -21.1 | <0.001 | <0.001 | 0.85 | 0.60 | -0.25 |
| cg25438517 | -0.24 | -21.1 | <0.001 | <0.001 | 0.79 | 0.55 | -0.24 |
| cg12303582 | -0.28 | -21.1 | <0.001 | <0.001 | 0.70 | 0.42 | -0.28 |
| cg02704949 | -0.26 | -21.1 | <0.001 | <0.001 | 0.74 | 0.48 | -0.26 |
| cg22237300 | -0.20 | -21.1 | <0.001 | <0.001 | 0.94 | 0.73 | -0.20 |
| cg15619820 | -0.33 | -21.1 | <0.001 | <0.001 | 0.80 | 0.47 | -0.33 |
| cg15357022 | -0.23 | -21.1 | <0.001 | <0.001 | 0.70 | 0.48 | -0.23 |
| cg21230427 | 0.25 | 21.1 | <0.001 | <0.001 | 0.19 | 0.44 | 0.25 |
| cg16371229 | 0.25 | 21.1 | <0.001 | <0.001 | 0.17 | 0.43 | 0.25 |
| cg15896939 | -0.26 | -21.1 | <0.001 | <0.001 | 0.77 | 0.50 | -0.26 |
| cg20993403 | -0.29 | -21.1 | <0.001 | <0.001 | 0.84 | 0.55 | -0.29 |
| cg23696248 | -0.21 | -21.1 | <0.001 | <0.001 | 0.90 | 0.69 | -0.21 |
| cg25102206 | -0.21 | -21.0 | <0.001 | <0.001 | 0.76 | 0.55 | -0.21 |
| cg15446845 | 0.21 | 21.0 | <0.001 | <0.001 | 0.15 | 0.37 | 0.21 |
| cg08256781 | -0.20 | -21.0 | <0.001 | <0.001 | 0.81 | 0.61 | -0.20 |
| cg03650119 | -0.33 | -21.0 | <0.001 | <0.001 | 0.68 | 0.35 | -0.33 |
| cg16708465 | 0.23 | 21.0 | <0.001 | <0.001 | 0.19 | 0.42 | 0.23 |
| cg05501357 | -0.24 | -21.0 | <0.001 | <0.001 | 0.78 | 0.54 | -0.24 |
| cg16852955 | -0.28 | -21.0 | <0.001 | <0.001 | 0.90 | 0.62 | -0.28 |
| cg05317096 | -0.25 | -21.0 | <0.001 | <0.001 | 0.80 | 0.54 | -0.25 |
| cg27656658 | -0.33 | -20.9 | <0.001 | <0.001 | 0.87 | 0.54 | -0.33 |
| cg18994033 | -0.21 | -20.9 | <0.001 | <0.001 | 0.66 | 0.45 | -0.21 |
| cg08482167 | -0.31 | -20.9 | <0.001 | <0.001 | 0.71 | 0.40 | -0.31 |
| cg24963001 | -0.29 | -20.9 | <0.001 | <0.001 | 0.73 | 0.43 | -0.29 |
| cg14560699 | -0.26 | -20.9 | <0.001 | <0.001 | 0.81 | 0.55 | -0.26 |
| cg18331061 | -0.22 | -20.9 | <0.001 | <0.001 | 0.88 | 0.67 | -0.22 |
| cg00376553 | -0.24 | -20.9 | <0.001 | <0.001 | 0.73 | 0.49 | -0.24 |
| cg06606381 | -0.20 | -20.9 | <0.001 | <0.001 | 0.80 | 0.60 | -0.20 |
| cg03729204 | -0.27 | -20.9 | <0.001 | <0.001 | 0.74 | 0.47 | -0.27 |
| cg19044229 | -0.21 | -20.9 | <0.001 | <0.001 | 0.75 | 0.54 | -0.21 |
| cg06783423 | 0.21 | 20.9 | <0.001 | <0.001 | 0.16 | 0.38 | 0.21 |
| cg09687907 | -0.31 | -20.9 | <0.001 | <0.001 | 0.65 | 0.33 | -0.31 |
| cg04962621 | 0.25 | 20.9 | <0.001 | <0.001 | 0.18 | 0.43 | 0.25 |
| cg25001102 | -0.32 | -20.9 | <0.001 | <0.001 | 0.71 | 0.39 | -0.32 |
| cg07785717 | -0.24 | -20.9 | <0.001 | <0.001 | 0.65 | 0.41 | -0.24 |
| cg24996161 | -0.27 | -20.9 | <0.001 | <0.001 | 0.76 | 0.49 | -0.27 |
| cg15848095 | -0.27 | -20.8 | <0.001 | <0.001 | 0.83 | 0.56 | -0.27 |
| cg09510085 | -0.29 | -20.8 | <0.001 | <0.001 | 0.66 | 0.37 | -0.29 |
| cg02691393 | -0.28 | -20.8 | <0.001 | <0.001 | 0.81 | 0.53 | -0.28 |
| cg13065504 | -0.24 | -20.8 | <0.001 | <0.001 | 0.77 | 0.53 | -0.24 |
| cg00273198 | -0.26 | -20.8 | <0.001 | <0.001 | 0.65 | 0.39 | -0.26 |
| cg00355909 | 0.25 | 20.8 | <0.001 | <0.001 | 0.09 | 0.35 | 0.25 |
| cg05964640 | 0.21 | 20.8 | <0.001 | <0.001 | 0.12 | 0.33 | 0.21 |
| cg02950892 | -0.25 | -20.8 | <0.001 | <0.001 | 0.77 | 0.51 | -0.25 |
| cg25911551 | -0.32 | -20.8 | <0.001 | <0.001 | 0.70 | 0.39 | -0.32 |
| cg25507767 | -0.27 | -20.8 | <0.001 | <0.001 | 0.64 | 0.37 | -0.27 |
| cg13527233 | -0.22 | -20.8 | <0.001 | <0.001 | 0.73 | 0.51 | -0.22 |
| cg18106923 | -0.30 | -20.8 | <0.001 | <0.001 | 0.62 | 0.32 | -0.30 |
| cg05719140 | -0.21 | -20.8 | <0.001 | <0.001 | 0.69 | 0.49 | -0.21 |
| cg05229035 | -0.25 | -20.7 | <0.001 | <0.001 | 0.81 | 0.56 | -0.25 |
| cg18586886 | 0.38 | 20.7 | <0.001 | <0.001 | 0.18 | 0.57 | 0.38 |
| cg10523140 | 0.20 | 20.7 | <0.001 | <0.001 | 0.19 | 0.40 | 0.20 |
| cg18435000 | -0.23 | -20.7 | <0.001 | <0.001 | 0.74 | 0.51 | -0.23 |
| cg05130816 | 0.21 | 20.7 | <0.001 | <0.001 | 0.19 | 0.40 | 0.21 |
| cg08893109 | -0.24 | -20.7 | <0.001 | <0.001 | 0.82 | 0.58 | -0.24 |
| cg25690589 | -0.22 | -20.7 | <0.001 | <0.001 | 0.69 | 0.47 | -0.22 |
| cg14431024 | -0.24 | -20.7 | <0.001 | <0.001 | 0.88 | 0.63 | -0.24 |
| cg13827582 | -0.26 | -20.7 | <0.001 | <0.001 | 0.83 | 0.57 | -0.26 |
| cg18771553 | -0.25 | -20.7 | <0.001 | <0.001 | 0.66 | 0.41 | -0.25 |
| cg19890739 | -0.21 | -20.7 | <0.001 | <0.001 | 0.62 | 0.41 | -0.21 |
| cg18465945 | -0.24 | -20.7 | <0.001 | <0.001 | 0.79 | 0.55 | -0.24 |
| cg13709639 | 0.25 | 20.7 | <0.001 | <0.001 | 0.16 | 0.42 | 0.25 |
| cg01961086 | -0.48 | -20.7 | <0.001 | <0.001 | 0.67 | 0.19 | -0.48 |
| cg24164254 | -0.21 | -20.7 | <0.001 | <0.001 | 0.70 | 0.49 | -0.21 |
| cg18833880 | -0.31 | -20.7 | <0.001 | <0.001 | 0.93 | 0.61 | -0.31 |
| cg02707176 | -0.33 | -20.7 | <0.001 | <0.001 | 0.70 | 0.37 | -0.33 |
| cg23986590 | -0.30 | -20.6 | <0.001 | <0.001 | 0.82 | 0.53 | -0.30 |
| cg18170989 | -0.34 | -20.6 | <0.001 | <0.001 | 0.72 | 0.38 | -0.34 |
| cg18147098 | -0.25 | -20.6 | <0.001 | <0.001 | 0.75 | 0.50 | -0.25 |
| cg23199335 | 0.24 | 20.6 | <0.001 | <0.001 | 0.12 | 0.36 | 0.24 |
| cg00012698 | -0.23 | -20.6 | <0.001 | <0.001 | 0.87 | 0.64 | -0.23 |
| cg24512973 | -0.24 | -20.6 | <0.001 | <0.001 | 0.89 | 0.64 | -0.24 |
| cg17822706 | 0.22 | 20.6 | <0.001 | <0.001 | 0.14 | 0.36 | 0.22 |
| cg11628880 | -0.26 | -20.6 | <0.001 | <0.001 | 0.63 | 0.38 | -0.26 |
| cg01286950 | -0.21 | -20.6 | <0.001 | <0.001 | 0.96 | 0.75 | -0.21 |
| cg14068176 | -0.24 | -20.6 | <0.001 | <0.001 | 0.90 | 0.66 | -0.24 |
| cg02788637 | 0.24 | 20.6 | <0.001 | <0.001 | 0.09 | 0.33 | 0.24 |
| cg26952618 | -0.21 | -20.5 | <0.001 | <0.001 | 0.65 | 0.43 | -0.21 |
| cg08519905 | -0.29 | -20.5 | <0.001 | <0.001 | 0.66 | 0.37 | -0.29 |
| cg07675682 | -0.22 | -20.5 | <0.001 | <0.001 | 0.74 | 0.52 | -0.22 |
| cg23586440 | -0.20 | -20.5 | <0.001 | <0.001 | 0.82 | 0.62 | -0.20 |
| cg15624376 | -0.23 | -20.5 | <0.001 | <0.001 | 0.85 | 0.62 | -0.23 |
| cg25230363 | 0.27 | 20.5 | <0.001 | <0.001 | 0.13 | 0.40 | 0.27 |
| cg06776201 | -0.25 | -20.5 | <0.001 | <0.001 | 0.84 | 0.60 | -0.25 |
| cg13280788 | -0.30 | -20.5 | <0.001 | <0.001 | 0.79 | 0.49 | -0.30 |
| cg00207921 | -0.30 | -20.5 | <0.001 | <0.001 | 0.76 | 0.46 | -0.30 |
| cg23267890 | -0.25 | -20.5 | <0.001 | <0.001 | 0.67 | 0.43 | -0.25 |
| cg07544796 | -0.30 | -20.4 | <0.001 | <0.001 | 0.71 | 0.41 | -0.30 |
| cg00426659 | -0.28 | -20.4 | <0.001 | <0.001 | 0.64 | 0.36 | -0.28 |
| cg01329005 | 0.32 | 20.4 | <0.001 | <0.001 | 0.18 | 0.50 | 0.32 |
| cg06644669 | 0.28 | 20.4 | <0.001 | <0.001 | 0.16 | 0.44 | 0.28 |
| cg11427534 | -0.26 | -20.4 | <0.001 | <0.001 | 0.81 | 0.55 | -0.26 |
| cg00962755 | -0.31 | -20.4 | <0.001 | <0.001 | 0.64 | 0.33 | -0.31 |
| cg08533865 | -0.26 | -20.4 | <0.001 | <0.001 | 0.90 | 0.64 | -0.26 |
| cg09038267 | -0.24 | -20.4 | <0.001 | <0.001 | 0.75 | 0.51 | -0.24 |
| cg16935039 | -0.23 | -20.4 | <0.001 | <0.001 | 0.79 | 0.55 | -0.23 |
| cg19387165 | -0.28 | -20.4 | <0.001 | <0.001 | 0.85 | 0.57 | -0.28 |
| cg12700039 | -0.24 | -20.4 | <0.001 | <0.001 | 0.74 | 0.50 | -0.24 |
| cg13293524 | -0.32 | -20.4 | <0.001 | <0.001 | 0.88 | 0.56 | -0.32 |
| cg12610087 | -0.28 | -20.4 | <0.001 | <0.001 | 0.65 | 0.38 | -0.28 |
| cg07350977 | -0.31 | -20.4 | <0.001 | <0.001 | 0.62 | 0.32 | -0.31 |
| cg14751552 | -0.21 | -20.4 | <0.001 | <0.001 | 0.82 | 0.61 | -0.21 |
| cg16066354 | 0.25 | 20.4 | <0.001 | <0.001 | 0.14 | 0.39 | 0.25 |
| cg01971181 | -0.27 | -20.4 | <0.001 | <0.001 | 0.71 | 0.44 | -0.27 |
| cg11565786 | -0.26 | -20.3 | <0.001 | <0.001 | 0.86 | 0.60 | -0.26 |
| cg14737994 | -0.24 | -20.3 | <0.001 | <0.001 | 0.67 | 0.44 | -0.24 |
| cg05911082 | -0.23 | -20.3 | <0.001 | <0.001 | 0.72 | 0.49 | -0.23 |
| cg25417842 | -0.27 | -20.3 | <0.001 | <0.001 | 0.79 | 0.52 | -0.27 |
| cg15018704 | -0.26 | -20.3 | <0.001 | <0.001 | 0.65 | 0.39 | -0.26 |
| cg04519622 | 0.21 | 20.3 | <0.001 | <0.001 | 0.20 | 0.41 | 0.21 |
| cg27026786 | -0.21 | -20.3 | <0.001 | <0.001 | 0.79 | 0.58 | -0.21 |
| cg07506081 | -0.21 | -20.2 | <0.001 | <0.001 | 0.73 | 0.52 | -0.21 |
| cg20176220 | -0.23 | -20.2 | <0.001 | <0.001 | 0.80 | 0.57 | -0.23 |
| cg00954841 | -0.25 | -20.2 | <0.001 | <0.001 | 0.79 | 0.54 | -0.25 |
| cg02803819 | -0.30 | -20.2 | <0.001 | <0.001 | 0.73 | 0.43 | -0.30 |
| cg00045902 | -0.21 | -20.2 | <0.001 | <0.001 | 0.61 | 0.40 | -0.21 |
| cg12626589 | -0.36 | -20.2 | <0.001 | <0.001 | 0.65 | 0.29 | -0.36 |
| cg23365801 | -0.24 | -20.2 | <0.001 | <0.001 | 0.66 | 0.42 | -0.24 |
| cg21045915 | -0.27 | -20.2 | <0.001 | <0.001 | 0.77 | 0.50 | -0.27 |
| cg13912224 | -0.26 | -20.2 | <0.001 | <0.001 | 0.74 | 0.48 | -0.26 |
| cg06786238 | -0.27 | -20.2 | <0.001 | <0.001 | 0.82 | 0.55 | -0.27 |
| cg10177032 | 0.29 | 20.2 | <0.001 | <0.001 | 0.11 | 0.40 | 0.29 |
| cg05803853 | -0.23 | -20.2 | <0.001 | <0.001 | 0.74 | 0.50 | -0.23 |
| cg21239179 | -0.22 | -20.2 | <0.001 | <0.001 | 0.74 | 0.52 | -0.22 |
| cg25578967 | -0.20 | -20.2 | <0.001 | <0.001 | 0.91 | 0.71 | -0.20 |
| cg20921659 | -0.22 | -20.2 | <0.001 | <0.001 | 0.69 | 0.47 | -0.22 |
| cg00168191 | -0.26 | -20.2 | <0.001 | <0.001 | 0.74 | 0.49 | -0.26 |
| cg18295923 | -0.22 | -20.2 | <0.001 | <0.001 | 0.82 | 0.59 | -0.22 |
| cg04537738 | -0.22 | -20.1 | <0.001 | <0.001 | 0.60 | 0.38 | -0.22 |
| cg24692310 | -0.25 | -20.1 | <0.001 | <0.001 | 0.78 | 0.53 | -0.25 |
| cg00031187 | -0.22 | -20.1 | <0.001 | <0.001 | 0.76 | 0.54 | -0.22 |
| cg13022129 | -0.22 | -20.1 | <0.001 | <0.001 | 0.77 | 0.55 | -0.22 |
| cg14022778 | -0.24 | -20.1 | <0.001 | <0.001 | 0.77 | 0.53 | -0.24 |
| cg25386676 | -0.30 | -20.1 | <0.001 | <0.001 | 0.63 | 0.33 | -0.30 |
| cg20740051 | -0.23 | -20.1 | <0.001 | <0.001 | 0.77 | 0.54 | -0.23 |
| cg09664492 | -0.22 | -20.1 | <0.001 | <0.001 | 0.74 | 0.53 | -0.22 |
| cg08120986 | -0.27 | -20.1 | <0.001 | <0.001 | 0.69 | 0.42 | -0.27 |
| cg18204273 | -0.22 | -20.1 | <0.001 | <0.001 | 0.82 | 0.60 | -0.22 |
| cg12427469 | -0.21 | -20.1 | <0.001 | <0.001 | 0.68 | 0.47 | -0.21 |
| cg16900589 | -0.23 | -20.0 | <0.001 | <0.001 | 0.94 | 0.72 | -0.23 |
| cg15833565 | -0.25 | -20.0 | <0.001 | <0.001 | 0.61 | 0.36 | -0.25 |
| cg26485825 | -0.27 | -20.0 | <0.001 | <0.001 | 0.68 | 0.41 | -0.27 |
| cg10533103 | -0.27 | -20.0 | <0.001 | <0.001 | 0.85 | 0.58 | -0.27 |
| cg05439665 | -0.25 | -20.0 | <0.001 | <0.001 | 0.65 | 0.40 | -0.25 |
| cg18081818 | -0.21 | -20.0 | <0.001 | <0.001 | 0.81 | 0.59 | -0.21 |
| cg11164649 | 0.22 | 20.0 | <0.001 | <0.001 | 0.19 | 0.41 | 0.22 |
| cg26480091 | -0.27 | -20.0 | <0.001 | <0.001 | 0.65 | 0.38 | -0.27 |
| cg03030419 | -0.23 | -20.0 | <0.001 | <0.001 | 0.76 | 0.53 | -0.23 |
| cg20432754 | -0.29 | -20.0 | <0.001 | <0.001 | 0.80 | 0.52 | -0.29 |
| cg09183138 | -0.22 | -20.0 | <0.001 | <0.001 | 0.89 | 0.67 | -0.22 |
| cg18648037 | -0.25 | -19.9 | <0.001 | <0.001 | 0.73 | 0.48 | -0.25 |
| cg04334243 | -0.30 | -19.9 | <0.001 | <0.001 | 0.66 | 0.37 | -0.30 |
| cg09664373 | -0.26 | -19.9 | <0.001 | <0.001 | 0.74 | 0.48 | -0.26 |
| cg14623715 | -0.34 | -19.9 | <0.001 | <0.001 | 0.63 | 0.29 | -0.34 |
| cg00925802 | -0.32 | -19.9 | <0.001 | <0.001 | 0.70 | 0.38 | -0.32 |
| cg12135344 | -0.21 | -19.9 | <0.001 | <0.001 | 0.73 | 0.51 | -0.21 |
| cg14063129 | -0.27 | -19.9 | <0.001 | <0.001 | 0.72 | 0.45 | -0.27 |
| cg19596468 | -0.22 | -19.9 | <0.001 | <0.001 | 0.69 | 0.47 | -0.22 |
| cg07535740 | -0.26 | -19.9 | <0.001 | <0.001 | 0.71 | 0.45 | -0.26 |
| cg10012530 | -0.21 | -19.9 | <0.001 | <0.001 | 0.70 | 0.49 | -0.21 |
| cg15275625 | 0.25 | 19.8 | <0.001 | <0.001 | 0.19 | 0.44 | 0.25 |
| cg18186763 | -0.22 | -19.8 | <0.001 | <0.001 | 0.75 | 0.53 | -0.22 |
| cg06946797 | 0.23 | 19.8 | <0.001 | <0.001 | 0.13 | 0.36 | 0.23 |
| cg24590395 | -0.30 | -19.8 | <0.001 | <0.001 | 0.60 | 0.30 | -0.30 |
| cg17475643 | -0.20 | -19.8 | <0.001 | <0.001 | 0.68 | 0.48 | -0.20 |
| cg20415486 | -0.24 | -19.8 | <0.001 | <0.001 | 0.89 | 0.65 | -0.24 |
| cg19047868 | -0.32 | -19.8 | <0.001 | <0.001 | 0.77 | 0.45 | -0.32 |
| cg07522913 | -0.27 | -19.8 | <0.001 | <0.001 | 0.63 | 0.36 | -0.27 |
| cg21754824 | -0.30 | -19.8 | <0.001 | <0.001 | 0.67 | 0.37 | -0.30 |
| cg00541718 | -0.21 | -19.8 | <0.001 | <0.001 | 0.71 | 0.50 | -0.21 |
| cg19846609 | -0.38 | -19.8 | <0.001 | <0.001 | 0.73 | 0.35 | -0.38 |
| cg12096487 | -0.34 | -19.8 | <0.001 | <0.001 | 0.77 | 0.43 | -0.34 |
| cg23623252 | -0.29 | -19.8 | <0.001 | <0.001 | 0.70 | 0.41 | -0.29 |
| cg15069235 | -0.29 | -19.8 | <0.001 | <0.001 | 0.69 | 0.40 | -0.29 |
| cg19521279 | -0.22 | -19.8 | <0.001 | <0.001 | 0.76 | 0.54 | -0.22 |
| cg03127558 | -0.30 | -19.8 | <0.001 | <0.001 | 0.87 | 0.58 | -0.30 |
| cg20076468 | -0.29 | -19.8 | <0.001 | <0.001 | 0.84 | 0.56 | -0.29 |
| cg14698646 | -0.22 | -19.7 | <0.001 | <0.001 | 0.88 | 0.65 | -0.22 |
| cg10949007 | 0.25 | 19.7 | <0.001 | <0.001 | 0.11 | 0.36 | 0.25 |
| cg05070571 | -0.27 | -19.7 | <0.001 | <0.001 | 0.83 | 0.56 | -0.27 |
| cg00952822 | -0.21 | -19.7 | <0.001 | <0.001 | 0.75 | 0.54 | -0.21 |
| cg18424208 | -0.21 | -19.7 | <0.001 | <0.001 | 0.89 | 0.68 | -0.21 |
| cg09986353 | -0.24 | -19.7 | <0.001 | <0.001 | 0.66 | 0.42 | -0.24 |
| cg01760189 | -0.23 | -19.7 | <0.001 | <0.001 | 0.89 | 0.66 | -0.23 |
| cg09761224 | -0.22 | -19.7 | <0.001 | <0.001 | 0.93 | 0.71 | -0.22 |
| cg00298065 | -0.22 | -19.7 | <0.001 | <0.001 | 0.62 | 0.40 | -0.22 |
| cg07159758 | -0.37 | -19.6 | <0.001 | <0.001 | 0.70 | 0.33 | -0.37 |
| cg12098750 | -0.34 | -19.6 | <0.001 | <0.001 | 0.66 | 0.31 | -0.34 |
| cg04149978 | -0.21 | -19.6 | <0.001 | <0.001 | 0.73 | 0.53 | -0.21 |
| cg03799405 | -0.32 | -19.6 | <0.001 | <0.001 | 0.69 | 0.37 | -0.32 |
| cg06581978 | 0.26 | 19.6 | <0.001 | <0.001 | 0.19 | 0.45 | 0.26 |
| cg19149899 | -0.24 | -19.6 | <0.001 | <0.001 | 0.79 | 0.54 | -0.24 |
| cg24761525 | -0.22 | -19.6 | <0.001 | <0.001 | 0.68 | 0.46 | -0.22 |
| cg10483525 | -0.21 | -19.6 | <0.001 | <0.001 | 0.61 | 0.41 | -0.21 |
| cg13005636 | -0.23 | -19.6 | <0.001 | <0.001 | 0.89 | 0.65 | -0.23 |
| cg27082921 | -0.35 | -19.6 | <0.001 | <0.001 | 0.77 | 0.43 | -0.35 |
| cg23752985 | -0.25 | -19.6 | <0.001 | <0.001 | 0.77 | 0.52 | -0.25 |
| cg02693328 | -0.21 | -19.6 | <0.001 | <0.001 | 0.76 | 0.55 | -0.21 |
| cg01202976 | -0.25 | -19.6 | <0.001 | <0.001 | 0.72 | 0.47 | -0.25 |
| cg20617977 | -0.20 | -19.6 | <0.001 | <0.001 | 0.74 | 0.54 | -0.20 |
| cg16583552 | -0.25 | -19.5 | <0.001 | <0.001 | 0.79 | 0.54 | -0.25 |
| cg17335199 | -0.31 | -19.5 | <0.001 | <0.001 | 0.61 | 0.30 | -0.31 |
| cg12148129 | 0.21 | 19.5 | <0.001 | <0.001 | 0.09 | 0.30 | 0.21 |
| cg19717773 | -0.33 | -19.5 | <0.001 | <0.001 | 0.75 | 0.43 | -0.33 |
| cg08384999 | -0.31 | -19.5 | <0.001 | <0.001 | 0.76 | 0.45 | -0.31 |
| cg09027601 | -0.25 | -19.5 | <0.001 | <0.001 | 0.64 | 0.39 | -0.25 |
| cg01180479 | -0.20 | -19.5 | <0.001 | <0.001 | 0.67 | 0.47 | -0.20 |
| cg07138437 | -0.23 | -19.5 | <0.001 | <0.001 | 0.74 | 0.51 | -0.23 |
| cg06984255 | -0.31 | -19.5 | <0.001 | <0.001 | 0.61 | 0.30 | -0.31 |
| cg14622443 | -0.23 | -19.5 | <0.001 | <0.001 | 0.81 | 0.58 | -0.23 |
| cg10655396 | 0.23 | 19.5 | <0.001 | <0.001 | 0.16 | 0.39 | 0.23 |
| cg13320257 | -0.25 | -19.5 | <0.001 | <0.001 | 0.82 | 0.57 | -0.25 |
| cg07338464 | -0.22 | -19.5 | <0.001 | <0.001 | 0.74 | 0.52 | -0.22 |
| cg14171944 | -0.26 | -19.5 | <0.001 | <0.001 | 0.87 | 0.61 | -0.26 |
| cg04998634 | 0.23 | 19.5 | <0.001 | <0.001 | 0.10 | 0.33 | 0.23 |
| cg17374433 | -0.36 | -19.5 | <0.001 | <0.001 | 0.67 | 0.31 | -0.36 |
| cg07922606 | -0.24 | -19.4 | <0.001 | <0.001 | 0.62 | 0.39 | -0.24 |
| cg15061330 | -0.24 | -19.4 | <0.001 | <0.001 | 0.74 | 0.50 | -0.24 |
| cg19476788 | -0.26 | -19.4 | <0.001 | <0.001 | 0.72 | 0.46 | -0.26 |
| cg20918393 | 0.25 | 19.4 | <0.001 | <0.001 | 0.19 | 0.44 | 0.25 |
| cg10336039 | -0.24 | -19.4 | <0.001 | <0.001 | 0.91 | 0.67 | -0.24 |
| cg13098379 | -0.25 | -19.4 | <0.001 | <0.001 | 0.85 | 0.60 | -0.25 |
| cg02546607 | -0.22 | -19.4 | <0.001 | <0.001 | 0.76 | 0.55 | -0.22 |
| cg12668523 | -0.31 | -19.4 | <0.001 | <0.001 | 0.79 | 0.49 | -0.31 |
| cg13875506 | -0.27 | -19.3 | <0.001 | <0.001 | 0.71 | 0.43 | -0.27 |
| cg22889914 | -0.25 | -19.3 | <0.001 | <0.001 | 0.83 | 0.58 | -0.25 |
| cg03899721 | -0.29 | -19.3 | <0.001 | <0.001 | 0.90 | 0.61 | -0.29 |
| cg20887442 | -0.24 | -19.3 | <0.001 | <0.001 | 0.89 | 0.65 | -0.24 |
| cg10279487 | 0.21 | 19.3 | <0.001 | <0.001 | 0.09 | 0.30 | 0.21 |
| cg18271964 | -0.27 | -19.3 | <0.001 | <0.001 | 0.66 | 0.39 | -0.27 |
| cg22730327 | -0.31 | -19.3 | <0.001 | <0.001 | 0.60 | 0.29 | -0.31 |
| cg15365426 | -0.21 | -19.3 | <0.001 | <0.001 | 0.82 | 0.61 | -0.21 |
| cg09545918 | -0.27 | -19.3 | <0.001 | <0.001 | 0.73 | 0.46 | -0.27 |
| cg27157482 | -0.22 | -19.3 | <0.001 | <0.001 | 0.64 | 0.42 | -0.22 |
| cg19411952 | -0.21 | -19.3 | <0.001 | <0.001 | 0.62 | 0.41 | -0.21 |
| cg14195216 | -0.32 | -19.3 | <0.001 | <0.001 | 0.63 | 0.31 | -0.32 |
| cg11076954 | -0.22 | -19.3 | <0.001 | <0.001 | 0.60 | 0.38 | -0.22 |
| cg16644803 | -0.21 | -19.3 | <0.001 | <0.001 | 0.80 | 0.59 | -0.21 |
| cg24853589 | -0.21 | -19.2 | <0.001 | <0.001 | 0.82 | 0.60 | -0.21 |
| cg10090414 | -0.27 | -19.2 | <0.001 | <0.001 | 0.79 | 0.52 | -0.27 |
| cg04340928 | -0.20 | -19.2 | <0.001 | <0.001 | 0.94 | 0.74 | -0.20 |
| cg01322214 | -0.23 | -19.2 | <0.001 | <0.001 | 0.69 | 0.47 | -0.23 |
| cg12313892 | -0.36 | -19.2 | <0.001 | <0.001 | 0.63 | 0.27 | -0.36 |
| cg09704116 | -0.26 | -19.2 | <0.001 | <0.001 | 0.64 | 0.38 | -0.26 |
| cg05555337 | -0.23 | -19.2 | <0.001 | <0.001 | 0.88 | 0.65 | -0.23 |
| cg12280664 | -0.37 | -19.2 | <0.001 | <0.001 | 0.71 | 0.34 | -0.37 |
| cg01206211 | -0.30 | -19.2 | <0.001 | <0.001 | 0.64 | 0.33 | -0.30 |
| cg24705426 | -0.32 | -19.2 | <0.001 | <0.001 | 0.61 | 0.29 | -0.32 |
| cg03308399 | -0.20 | -19.2 | <0.001 | <0.001 | 0.69 | 0.49 | -0.20 |
| cg16710348 | -0.20 | -19.2 | <0.001 | <0.001 | 0.87 | 0.67 | -0.20 |
| cg27428208 | -0.29 | -19.2 | <0.001 | <0.001 | 0.72 | 0.43 | -0.29 |
| cg10982443 | -0.34 | -19.2 | <0.001 | <0.001 | 0.68 | 0.34 | -0.34 |
| cg21106505 | -0.24 | -19.2 | <0.001 | <0.001 | 0.84 | 0.60 | -0.24 |
| cg00527307 | -0.29 | -19.2 | <0.001 | <0.001 | 0.71 | 0.42 | -0.29 |
| cg11173579 | -0.25 | -19.1 | <0.001 | <0.001 | 0.88 | 0.63 | -0.25 |
| cg20704654 | -0.24 | -19.1 | <0.001 | <0.001 | 0.79 | 0.55 | -0.24 |
| cg10369242 | -0.25 | -19.1 | <0.001 | <0.001 | 0.82 | 0.58 | -0.25 |
| cg01152726 | -0.21 | -19.1 | <0.001 | <0.001 | 0.84 | 0.64 | -0.21 |
| cg23250540 | -0.24 | -19.1 | <0.001 | <0.001 | 0.84 | 0.60 | -0.24 |
| cg00880157 | -0.22 | -19.1 | <0.001 | <0.001 | 0.64 | 0.42 | -0.22 |
| cg17156570 | -0.27 | -19.1 | <0.001 | <0.001 | 0.62 | 0.35 | -0.27 |
| cg27151770 | -0.27 | -19.1 | <0.001 | <0.001 | 0.73 | 0.46 | -0.27 |
| cg23021076 | -0.21 | -19.1 | <0.001 | <0.001 | 0.80 | 0.60 | -0.21 |
| cg05446471 | -0.24 | -19.1 | <0.001 | <0.001 | 0.85 | 0.61 | -0.24 |
| cg09825979 | -0.26 | -19.1 | <0.001 | <0.001 | 0.66 | 0.41 | -0.26 |
| cg13661519 | -0.24 | -19.1 | <0.001 | <0.001 | 0.74 | 0.49 | -0.24 |
| cg26174326 | -0.24 | -19.1 | <0.001 | <0.001 | 0.84 | 0.61 | -0.24 |
| cg00341885 | -0.25 | -19.1 | <0.001 | <0.001 | 0.89 | 0.64 | -0.25 |
| cg06893296 | -0.28 | -19.1 | <0.001 | <0.001 | 0.65 | 0.38 | -0.28 |
| cg27059228 | 0.23 | 19.1 | <0.001 | <0.001 | 0.18 | 0.40 | 0.23 |
| cg26407571 | -0.29 | -19.1 | <0.001 | <0.001 | 0.80 | 0.51 | -0.29 |
| cg20299414 | -0.24 | -19.1 | <0.001 | <0.001 | 0.74 | 0.49 | -0.24 |
| cg09341491 | -0.21 | -19.1 | <0.001 | <0.001 | 0.67 | 0.46 | -0.21 |
| cg05457480 | -0.23 | -19.0 | <0.001 | <0.001 | 0.61 | 0.37 | -0.23 |
| cg26396492 | 0.21 | 19.0 | <0.001 | <0.001 | 0.16 | 0.36 | 0.21 |
| cg27598107 | -0.31 | -19.0 | <0.001 | <0.001 | 0.65 | 0.34 | -0.31 |
| cg06691343 | -0.22 | -19.0 | <0.001 | <0.001 | 0.80 | 0.58 | -0.22 |
| cg26120787 | -0.39 | -19.0 | <0.001 | <0.001 | 0.64 | 0.25 | -0.39 |
| cg23238315 | -0.36 | -19.0 | <0.001 | <0.001 | 0.71 | 0.35 | -0.36 |
| cg22241045 | -0.25 | -19.0 | <0.001 | <0.001 | 0.75 | 0.49 | -0.25 |
| cg11668133 | -0.23 | -19.0 | <0.001 | <0.001 | 0.81 | 0.58 | -0.23 |
| cg07532353 | -0.40 | -19.0 | <0.001 | <0.001 | 0.64 | 0.25 | -0.40 |
| cg01414882 | -0.23 | -19.0 | <0.001 | <0.001 | 0.63 | 0.41 | -0.23 |
| cg09144964 | -0.20 | -19.0 | <0.001 | <0.001 | 0.61 | 0.41 | -0.20 |
| cg02342906 | -0.21 | -19.0 | <0.001 | <0.001 | 0.85 | 0.64 | -0.21 |
| cg25726128 | -0.22 | -19.0 | <0.001 | <0.001 | 0.89 | 0.67 | -0.22 |
| cg01351315 | -0.26 | -19.0 | <0.001 | <0.001 | 0.77 | 0.51 | -0.26 |
| cg09658497 | -0.32 | -19.0 | <0.001 | <0.001 | 0.85 | 0.52 | -0.32 |
| cg26742275 | -0.21 | -19.0 | <0.001 | <0.001 | 0.79 | 0.57 | -0.21 |
| cg01899130 | 0.22 | 19.0 | <0.001 | <0.001 | 0.11 | 0.33 | 0.22 |
| cg05217312 | -0.27 | -19.0 | <0.001 | <0.001 | 0.75 | 0.48 | -0.27 |
| cg08873746 | -0.22 | -19.0 | <0.001 | <0.001 | 0.73 | 0.51 | -0.22 |
| cg08091526 | -0.24 | -18.9 | <0.001 | <0.001 | 0.80 | 0.56 | -0.24 |
| cg19149915 | -0.21 | -18.9 | <0.001 | <0.001 | 0.90 | 0.68 | -0.21 |
| cg08655206 | -0.25 | -18.9 | <0.001 | <0.001 | 0.71 | 0.46 | -0.25 |
| cg14476745 | -0.31 | -18.9 | <0.001 | <0.001 | 0.73 | 0.42 | -0.31 |
| cg16783349 | -0.41 | -18.9 | <0.001 | <0.001 | 0.70 | 0.29 | -0.41 |
| cg04074945 | -0.26 | -18.9 | <0.001 | <0.001 | 0.73 | 0.47 | -0.26 |
| cg23217463 | -0.25 | -18.9 | <0.001 | <0.001 | 0.90 | 0.66 | -0.25 |
| cg23146833 | 0.21 | 18.9 | <0.001 | <0.001 | 0.17 | 0.38 | 0.21 |
| cg09987620 | -0.22 | -18.9 | <0.001 | <0.001 | 0.92 | 0.70 | -0.22 |
| cg25967031 | -0.24 | -18.9 | <0.001 | <0.001 | 0.82 | 0.57 | -0.24 |
| cg19883472 | -0.29 | -18.9 | <0.001 | <0.001 | 0.82 | 0.54 | -0.29 |
| cg18992570 | -0.28 | -18.9 | <0.001 | <0.001 | 0.74 | 0.47 | -0.28 |
| cg13342634 | -0.30 | -18.9 | <0.001 | <0.001 | 0.64 | 0.34 | -0.30 |
| cg19075717 | -0.21 | -18.9 | <0.001 | <0.001 | 0.80 | 0.59 | -0.21 |
| cg24002541 | -0.21 | -18.9 | <0.001 | <0.001 | 0.70 | 0.50 | -0.21 |
| cg01806382 | -0.28 | -18.9 | <0.001 | <0.001 | 0.87 | 0.59 | -0.28 |
| cg23209606 | -0.20 | -18.9 | <0.001 | <0.001 | 0.77 | 0.57 | -0.20 |
| cg14897263 | -0.25 | -18.8 | <0.001 | <0.001 | 0.61 | 0.36 | -0.25 |
| cg01469132 | -0.23 | -18.8 | <0.001 | <0.001 | 0.88 | 0.65 | -0.23 |
| cg11863380 | -0.29 | -18.8 | <0.001 | <0.001 | 0.79 | 0.50 | -0.29 |
| cg19572487 | 0.23 | 18.8 | <0.001 | <0.001 | 0.15 | 0.37 | 0.23 |
| cg01319323 | -0.23 | -18.8 | <0.001 | <0.001 | 0.83 | 0.60 | -0.23 |
| cg02836478 | -0.27 | -18.8 | <0.001 | <0.001 | 0.77 | 0.50 | -0.27 |
| cg14852082 | -0.28 | -18.8 | <0.001 | <0.001 | 0.61 | 0.33 | -0.28 |
| cg16797901 | -0.29 | -18.8 | <0.001 | <0.001 | 0.66 | 0.37 | -0.29 |
| cg06789445 | -0.20 | -18.8 | <0.001 | <0.001 | 0.80 | 0.60 | -0.20 |
| cg00143527 | 0.24 | 18.8 | <0.001 | <0.001 | 0.20 | 0.44 | 0.24 |
| cg19304273 | -0.24 | -18.8 | <0.001 | <0.001 | 0.69 | 0.45 | -0.24 |
| cg20490341 | -0.22 | -18.8 | <0.001 | <0.001 | 0.85 | 0.63 | -0.22 |
| cg06385583 | 0.22 | 18.8 | <0.001 | <0.001 | 0.16 | 0.39 | 0.22 |
| cg06962787 | -0.25 | -18.8 | <0.001 | <0.001 | 0.73 | 0.48 | -0.25 |
| cg25814293 | -0.24 | -18.8 | <0.001 | <0.001 | 0.89 | 0.65 | -0.24 |
| cg14649262 | -0.29 | -18.7 | <0.001 | <0.001 | 0.68 | 0.39 | -0.29 |
| cg03785076 | -0.21 | -18.7 | <0.001 | <0.001 | 0.61 | 0.40 | -0.21 |
| cg09040942 | -0.25 | -18.7 | <0.001 | <0.001 | 0.92 | 0.67 | -0.25 |
| cg17109042 | -0.24 | -18.7 | <0.001 | <0.001 | 0.68 | 0.44 | -0.24 |
| cg27460824 | -0.21 | -18.7 | <0.001 | <0.001 | 0.82 | 0.61 | -0.21 |
| cg02722596 | -0.32 | -18.7 | <0.001 | <0.001 | 0.67 | 0.35 | -0.32 |
| cg18064917 | -0.21 | -18.7 | <0.001 | <0.001 | 0.84 | 0.63 | -0.21 |
| cg20631820 | -0.20 | -18.7 | <0.001 | <0.001 | 0.89 | 0.69 | -0.20 |
| cg02650667 | -0.26 | -18.7 | <0.001 | <0.001 | 0.78 | 0.52 | -0.26 |
| cg10642330 | -0.22 | -18.7 | <0.001 | <0.001 | 0.77 | 0.55 | -0.22 |
| cg21553980 | -0.25 | -18.7 | <0.001 | <0.001 | 0.79 | 0.54 | -0.25 |
| cg17207736 | -0.26 | -18.7 | <0.001 | <0.001 | 0.69 | 0.42 | -0.26 |
| cg03447699 | -0.20 | -18.7 | <0.001 | <0.001 | 0.65 | 0.45 | -0.20 |
| cg17646499 | -0.22 | -18.7 | <0.001 | <0.001 | 0.86 | 0.65 | -0.22 |
| cg03615683 | -0.21 | -18.7 | <0.001 | <0.001 | 0.62 | 0.41 | -0.21 |
| cg25902889 | -0.33 | -18.6 | <0.001 | <0.001 | 0.68 | 0.36 | -0.33 |
| cg23781334 | -0.29 | -18.6 | <0.001 | <0.001 | 0.82 | 0.53 | -0.29 |
| cg10941445 | -0.21 | -18.6 | <0.001 | <0.001 | 0.70 | 0.49 | -0.21 |
| cg13635184 | -0.20 | -18.6 | <0.001 | <0.001 | 0.88 | 0.67 | -0.20 |
| cg26075259 | -0.20 | -18.6 | <0.001 | <0.001 | 0.77 | 0.56 | -0.20 |
| cg14523979 | -0.25 | -18.6 | <0.001 | <0.001 | 0.72 | 0.47 | -0.25 |
| cg00551143 | -0.21 | -18.6 | <0.001 | <0.001 | 0.89 | 0.69 | -0.21 |
| cg12664749 | -0.27 | -18.6 | <0.001 | <0.001 | 0.67 | 0.40 | -0.27 |
| cg05477823 | -0.27 | -18.6 | <0.001 | <0.001 | 0.69 | 0.42 | -0.27 |
| cg14266527 | -0.26 | -18.6 | <0.001 | <0.001 | 0.63 | 0.37 | -0.26 |
| cg18658151 | -0.26 | -18.6 | <0.001 | <0.001 | 0.83 | 0.58 | -0.26 |
| cg04233230 | -0.22 | -18.6 | <0.001 | <0.001 | 0.63 | 0.41 | -0.22 |
| cg06492744 | 0.25 | 18.6 | <0.001 | <0.001 | 0.18 | 0.43 | 0.25 |
| cg16989822 | -0.22 | -18.6 | <0.001 | <0.001 | 0.87 | 0.65 | -0.22 |
| cg14089503 | -0.26 | -18.5 | <0.001 | <0.001 | 0.65 | 0.39 | -0.26 |
| cg23263937 | -0.25 | -18.5 | <0.001 | <0.001 | 0.76 | 0.51 | -0.25 |
| cg20911718 | -0.21 | -18.5 | <0.001 | <0.001 | 0.66 | 0.45 | -0.21 |
| cg16829244 | -0.22 | -18.5 | <0.001 | <0.001 | 0.80 | 0.58 | -0.22 |
| cg26979299 | -0.24 | -18.5 | <0.001 | <0.001 | 0.70 | 0.46 | -0.24 |
| cg22365906 | -0.27 | -18.5 | <0.001 | <0.001 | 0.67 | 0.40 | -0.27 |
| cg14791525 | -0.21 | -18.5 | <0.001 | <0.001 | 0.87 | 0.66 | -0.21 |
| cg11869862 | -0.21 | -18.5 | <0.001 | <0.001 | 0.62 | 0.41 | -0.21 |
| cg21211730 | -0.24 | -18.5 | <0.001 | <0.001 | 0.74 | 0.50 | -0.24 |
| cg07355507 | -0.24 | -18.5 | <0.001 | <0.001 | 0.89 | 0.65 | -0.24 |
| cg17616537 | -0.24 | -18.5 | <0.001 | <0.001 | 0.85 | 0.61 | -0.24 |
| cg14158583 | -0.23 | -18.5 | <0.001 | <0.001 | 0.80 | 0.57 | -0.23 |
| cg25824127 | -0.22 | -18.5 | <0.001 | <0.001 | 0.87 | 0.65 | -0.22 |
| cg09096555 | -0.23 | -18.5 | <0.001 | <0.001 | 0.63 | 0.40 | -0.23 |
| cg19439043 | -0.23 | -18.5 | <0.001 | <0.001 | 0.80 | 0.58 | -0.23 |
| cg07288447 | -0.24 | -18.4 | <0.001 | <0.001 | 0.76 | 0.51 | -0.24 |
| cg25894019 | -0.27 | -18.4 | <0.001 | <0.001 | 0.83 | 0.57 | -0.27 |
| cg25316769 | 0.22 | 18.4 | <0.001 | <0.001 | 0.19 | 0.41 | 0.22 |
| cg05728282 | -0.20 | -18.4 | <0.001 | <0.001 | 0.63 | 0.43 | -0.20 |
| cg08848462 | -0.22 | -18.4 | <0.001 | <0.001 | 0.63 | 0.41 | -0.22 |
| cg16922039 | -0.22 | -18.4 | <0.001 | <0.001 | 0.67 | 0.46 | -0.22 |
| cg13288195 | -0.20 | -18.4 | <0.001 | <0.001 | 0.73 | 0.52 | -0.20 |
| cg00028211 | -0.30 | -18.4 | <0.001 | <0.001 | 0.70 | 0.40 | -0.30 |
| cg20492034 | -0.26 | -18.4 | <0.001 | <0.001 | 0.68 | 0.42 | -0.26 |
| cg25351606 | -0.24 | -18.4 | <0.001 | <0.001 | 0.69 | 0.46 | -0.24 |
| cg13077031 | -0.22 | -18.4 | <0.001 | <0.001 | 0.72 | 0.50 | -0.22 |
| cg10165801 | -0.25 | -18.4 | <0.001 | <0.001 | 0.70 | 0.45 | -0.25 |
| cg26645242 | -0.29 | -18.3 | <0.001 | <0.001 | 0.76 | 0.46 | -0.29 |
| cg00103299 | -0.24 | -18.3 | <0.001 | <0.001 | 0.70 | 0.46 | -0.24 |
| cg00188627 | -0.22 | -18.3 | <0.001 | <0.001 | 0.64 | 0.42 | -0.22 |
| cg01239928 | -0.24 | -18.3 | <0.001 | <0.001 | 0.77 | 0.53 | -0.24 |
| cg16692735 | -0.24 | -18.3 | <0.001 | <0.001 | 0.69 | 0.45 | -0.24 |
| cg02164615 | -0.29 | -18.3 | <0.001 | <0.001 | 0.62 | 0.34 | -0.29 |
| cg21840233 | -0.21 | -18.3 | <0.001 | <0.001 | 0.82 | 0.61 | -0.21 |
| cg02520281 | -0.21 | -18.3 | <0.001 | <0.001 | 0.64 | 0.44 | -0.21 |
| cg15531536 | -0.25 | -18.3 | <0.001 | <0.001 | 0.82 | 0.57 | -0.25 |
| cg01109574 | -0.30 | -18.3 | <0.001 | <0.001 | 0.71 | 0.41 | -0.30 |
| cg04208996 | -0.20 | -18.3 | <0.001 | <0.001 | 0.87 | 0.67 | -0.20 |
| cg05648614 | -0.24 | -18.3 | <0.001 | <0.001 | 0.74 | 0.50 | -0.24 |
| cg00988037 | -0.20 | -18.3 | <0.001 | <0.001 | 0.61 | 0.41 | -0.20 |
| cg08842032 | -0.22 | -18.3 | <0.001 | <0.001 | 0.81 | 0.59 | -0.22 |
| cg06870213 | -0.27 | -18.3 | <0.001 | <0.001 | 0.60 | 0.33 | -0.27 |
| cg18971175 | -0.21 | -18.2 | <0.001 | <0.001 | 0.76 | 0.55 | -0.21 |
| cg03679504 | -0.25 | -18.2 | <0.001 | <0.001 | 0.89 | 0.64 | -0.25 |
| cg19022697 | -0.20 | -18.2 | <0.001 | <0.001 | 0.66 | 0.46 | -0.20 |
| cg20782724 | -0.20 | -18.2 | <0.001 | <0.001 | 0.73 | 0.53 | -0.20 |
| cg12573502 | -0.21 | -18.2 | <0.001 | <0.001 | 0.88 | 0.67 | -0.21 |
| cg04719574 | 0.24 | 18.2 | <0.001 | <0.001 | 0.18 | 0.42 | 0.24 |
| cg24369989 | -0.21 | -18.2 | <0.001 | <0.001 | 0.70 | 0.49 | -0.21 |
| cg21663666 | -0.20 | -18.2 | <0.001 | <0.001 | 0.94 | 0.74 | -0.20 |
| cg18623658 | -0.22 | -18.2 | <0.001 | <0.001 | 0.79 | 0.57 | -0.22 |
| cg08949329 | -0.22 | -18.2 | <0.001 | <0.001 | 0.73 | 0.51 | -0.22 |
| cg15196806 | -0.20 | -18.2 | <0.001 | <0.001 | 0.74 | 0.54 | -0.20 |
| cg02936049 | -0.22 | -18.2 | <0.001 | <0.001 | 0.77 | 0.55 | -0.22 |
| cg18899220 | -0.27 | -18.2 | <0.001 | <0.001 | 0.79 | 0.52 | -0.27 |
| cg10013501 | -0.28 | -18.2 | <0.001 | <0.001 | 0.61 | 0.33 | -0.28 |
| cg13248581 | -0.26 | -18.2 | <0.001 | <0.001 | 0.61 | 0.35 | -0.26 |
| cg01699740 | -0.24 | -18.2 | <0.001 | <0.001 | 0.86 | 0.62 | -0.24 |
| cg11331739 | -0.25 | -18.2 | <0.001 | <0.001 | 0.67 | 0.43 | -0.25 |
| cg09515921 | -0.23 | -18.2 | <0.001 | <0.001 | 0.90 | 0.67 | -0.23 |
| cg03025473 | -0.21 | -18.2 | <0.001 | <0.001 | 0.82 | 0.60 | -0.21 |
| cg16591381 | -0.21 | -18.2 | <0.001 | <0.001 | 0.88 | 0.67 | -0.21 |
| cg15635633 | -0.26 | -18.2 | <0.001 | <0.001 | 0.76 | 0.49 | -0.26 |
| cg12476188 | -0.22 | -18.1 | <0.001 | <0.001 | 0.67 | 0.45 | -0.22 |
| cg03717503 | -0.22 | -18.1 | <0.001 | <0.001 | 0.84 | 0.62 | -0.22 |
| cg01156249 | 0.29 | 18.1 | <0.001 | <0.001 | 0.13 | 0.42 | 0.29 |
| cg01993208 | -0.25 | -18.1 | <0.001 | <0.001 | 0.61 | 0.37 | -0.25 |
| cg08884928 | -0.21 | -18.1 | <0.001 | <0.001 | 0.64 | 0.42 | -0.21 |
| cg06544693 | -0.21 | -18.1 | <0.001 | <0.001 | 0.79 | 0.58 | -0.21 |
| cg17837069 | 0.22 | 18.1 | <0.001 | <0.001 | 0.16 | 0.38 | 0.22 |
| cg11974498 | -0.25 | -18.1 | <0.001 | <0.001 | 0.64 | 0.39 | -0.25 |
| cg08138586 | -0.21 | -18.1 | <0.001 | <0.001 | 0.80 | 0.60 | -0.21 |
| cg03763508 | -0.24 | -18.1 | <0.001 | <0.001 | 0.72 | 0.48 | -0.24 |
| cg16018002 | -0.26 | -18.1 | <0.001 | <0.001 | 0.65 | 0.39 | -0.26 |
| cg17482089 | -0.24 | -18.1 | <0.001 | <0.001 | 0.64 | 0.40 | -0.24 |
| cg24137774 | -0.34 | -18.1 | <0.001 | <0.001 | 0.74 | 0.39 | -0.34 |
| cg12200124 | 0.21 | 18.1 | <0.001 | <0.001 | 0.20 | 0.40 | 0.21 |
| cg16151082 | -0.21 | -18.1 | <0.001 | <0.001 | 0.60 | 0.39 | -0.21 |
| cg06736000 | -0.28 | -18.1 | <0.001 | <0.001 | 0.69 | 0.41 | -0.28 |
| cg12057127 | -0.23 | -18.1 | <0.001 | <0.001 | 0.68 | 0.45 | -0.23 |
| cg08067346 | -0.21 | -18.1 | <0.001 | <0.001 | 0.71 | 0.49 | -0.21 |
| cg27580229 | -0.22 | -18.0 | <0.001 | <0.001 | 0.81 | 0.59 | -0.22 |
| cg15022051 | -0.25 | -18.0 | <0.001 | <0.001 | 0.80 | 0.55 | -0.25 |
| cg16878595 | -0.24 | -18.0 | <0.001 | <0.001 | 0.89 | 0.65 | -0.24 |
| cg12910797 | -0.26 | -18.0 | <0.001 | <0.001 | 0.82 | 0.55 | -0.26 |
| cg22198044 | -0.30 | -18.0 | <0.001 | <0.001 | 0.63 | 0.33 | -0.30 |
| cg11152302 | -0.21 | -18.0 | <0.001 | <0.001 | 0.65 | 0.44 | -0.21 |
| cg01286133 | -0.27 | -18.0 | <0.001 | <0.001 | 0.73 | 0.45 | -0.27 |
| cg26508200 | -0.22 | -18.0 | <0.001 | <0.001 | 0.88 | 0.66 | -0.22 |
| cg00240860 | -0.25 | -18.0 | <0.001 | <0.001 | 0.68 | 0.43 | -0.25 |
| cg15518113 | -0.22 | -18.0 | <0.001 | <0.001 | 0.85 | 0.63 | -0.22 |
| cg01149264 | -0.23 | -18.0 | <0.001 | <0.001 | 0.84 | 0.61 | -0.23 |
| cg10093067 | -0.24 | -18.0 | <0.001 | <0.001 | 0.71 | 0.46 | -0.24 |
| cg20592766 | -0.21 | -18.0 | <0.001 | <0.001 | 0.70 | 0.48 | -0.21 |
| cg24042578 | -0.21 | -18.0 | <0.001 | <0.001 | 0.71 | 0.50 | -0.21 |
| cg20095656 | -0.26 | -18.0 | <0.001 | <0.001 | 0.72 | 0.45 | -0.26 |
| cg22408430 | -0.23 | -18.0 | <0.001 | <0.001 | 0.84 | 0.61 | -0.23 |
| cg01969701 | -0.28 | -18.0 | <0.001 | <0.001 | 0.63 | 0.35 | -0.28 |
| cg04088289 | -0.25 | -17.9 | <0.001 | <0.001 | 0.89 | 0.63 | -0.25 |
| cg19106893 | -0.22 | -17.9 | <0.001 | <0.001 | 0.76 | 0.53 | -0.22 |
| cg26904049 | -0.21 | -17.9 | <0.001 | <0.001 | 0.67 | 0.45 | -0.21 |
| cg18446336 | -0.23 | -17.9 | <0.001 | <0.001 | 0.65 | 0.42 | -0.23 |
| cg13419925 | -0.24 | -17.9 | <0.001 | <0.001 | 0.68 | 0.44 | -0.24 |
| cg19351954 | -0.38 | -17.9 | <0.001 | <0.001 | 0.60 | 0.22 | -0.38 |
| cg21609526 | -0.30 | -17.9 | <0.001 | <0.001 | 0.69 | 0.39 | -0.30 |
| cg03466525 | -0.23 | -17.9 | <0.001 | <0.001 | 0.66 | 0.42 | -0.23 |
| cg00626312 | -0.22 | -17.9 | <0.001 | <0.001 | 0.73 | 0.51 | -0.22 |
| cg04042861 | -0.23 | -17.9 | <0.001 | <0.001 | 0.68 | 0.46 | -0.23 |
| cg11827925 | 0.25 | 17.9 | <0.001 | <0.001 | 0.20 | 0.45 | 0.25 |
| cg10483275 | -0.21 | -17.9 | <0.001 | <0.001 | 0.70 | 0.49 | -0.21 |
| cg11349594 | -0.27 | -17.9 | <0.001 | <0.001 | 0.70 | 0.43 | -0.27 |
| cg02711510 | -0.22 | -17.9 | <0.001 | <0.001 | 0.83 | 0.61 | -0.22 |
| cg02906741 | -0.24 | -17.9 | <0.001 | <0.001 | 0.85 | 0.61 | -0.24 |
| cg22689909 | 0.26 | 17.9 | <0.001 | <0.001 | 0.15 | 0.42 | 0.26 |
| cg19076587 | -0.22 | -17.9 | <0.001 | <0.001 | 0.80 | 0.59 | -0.22 |
| cg25023684 | -0.20 | -17.9 | <0.001 | <0.001 | 0.69 | 0.49 | -0.20 |
| cg01020475 | -0.25 | -17.9 | <0.001 | <0.001 | 0.92 | 0.68 | -0.25 |
| cg05387167 | -0.26 | -17.9 | <0.001 | <0.001 | 0.82 | 0.55 | -0.26 |
| cg15012214 | -0.28 | -17.9 | <0.001 | <0.001 | 0.62 | 0.35 | -0.28 |
| cg08085909 | -0.25 | -17.8 | <0.001 | <0.001 | 0.76 | 0.52 | -0.25 |
| cg20087519 | -0.20 | -17.8 | <0.001 | <0.001 | 0.63 | 0.43 | -0.20 |
| cg26291600 | -0.23 | -17.8 | <0.001 | <0.001 | 0.67 | 0.43 | -0.23 |
| cg24553170 | -0.25 | -17.8 | <0.001 | <0.001 | 0.65 | 0.40 | -0.25 |
| cg14496169 | -0.25 | -17.8 | <0.001 | <0.001 | 0.74 | 0.49 | -0.25 |
| cg17976205 | -0.27 | -17.8 | <0.001 | <0.001 | 0.77 | 0.50 | -0.27 |
| cg07836226 | -0.21 | -17.8 | <0.001 | <0.001 | 0.80 | 0.59 | -0.21 |
| cg03771840 | -0.23 | -17.8 | <0.001 | <0.001 | 0.72 | 0.49 | -0.23 |
| cg05131696 | -0.29 | -17.8 | <0.001 | <0.001 | 0.68 | 0.39 | -0.29 |
| cg20941258 | -0.29 | -17.8 | <0.001 | <0.001 | 0.64 | 0.35 | -0.29 |
| cg02657828 | -0.22 | -17.8 | <0.001 | <0.001 | 0.63 | 0.41 | -0.22 |
| cg24587601 | 0.24 | 17.8 | <0.001 | <0.001 | 0.20 | 0.44 | 0.24 |
| cg18512948 | -0.21 | -17.8 | <0.001 | <0.001 | 0.65 | 0.44 | -0.21 |
| cg11363972 | -0.22 | -17.8 | <0.001 | <0.001 | 0.61 | 0.39 | -0.22 |
| cg06391412 | -0.23 | -17.8 | <0.001 | <0.001 | 0.65 | 0.42 | -0.23 |
| cg26345105 | -0.24 | -17.8 | <0.001 | <0.001 | 0.61 | 0.37 | -0.24 |
| cg25317315 | -0.20 | -17.8 | <0.001 | <0.001 | 0.68 | 0.48 | -0.20 |
| cg12430062 | -0.26 | -17.8 | <0.001 | <0.001 | 0.81 | 0.56 | -0.26 |
| cg18864179 | -0.25 | -17.8 | <0.001 | <0.001 | 0.61 | 0.36 | -0.25 |
| cg01834541 | -0.25 | -17.7 | <0.001 | <0.001 | 0.70 | 0.45 | -0.25 |
| cg15303382 | -0.20 | -17.7 | <0.001 | <0.001 | 0.70 | 0.50 | -0.20 |
| cg14988680 | -0.21 | -17.7 | <0.001 | <0.001 | 0.61 | 0.40 | -0.21 |
| cg07927257 | -0.22 | -17.7 | <0.001 | <0.001 | 0.61 | 0.39 | -0.22 |
| cg19698309 | -0.21 | -17.7 | <0.001 | <0.001 | 0.77 | 0.56 | -0.21 |
| cg16857548 | -0.22 | -17.7 | <0.001 | <0.001 | 0.60 | 0.38 | -0.22 |
| cg17980404 | 0.22 | 17.7 | <0.001 | <0.001 | 0.20 | 0.42 | 0.22 |
| cg10251229 | -0.20 | -17.7 | <0.001 | <0.001 | 0.75 | 0.55 | -0.20 |
| cg22718636 | -0.27 | -17.7 | <0.001 | <0.001 | 0.68 | 0.41 | -0.27 |
| cg20823348 | -0.27 | -17.7 | <0.001 | <0.001 | 0.61 | 0.33 | -0.27 |
| cg20481640 | -0.24 | -17.7 | <0.001 | <0.001 | 0.71 | 0.47 | -0.24 |
| cg04947680 | -0.21 | -17.7 | <0.001 | <0.001 | 0.60 | 0.40 | -0.21 |
| cg02452639 | -0.26 | -17.7 | <0.001 | <0.001 | 0.72 | 0.46 | -0.26 |
| cg02137183 | -0.21 | -17.7 | <0.001 | <0.001 | 0.81 | 0.60 | -0.21 |
| cg06154311 | -0.27 | -17.7 | <0.001 | <0.001 | 0.64 | 0.37 | -0.27 |
| cg10709708 | -0.24 | -17.7 | <0.001 | <0.001 | 0.75 | 0.51 | -0.24 |
| cg06689816 | -0.23 | -17.7 | <0.001 | <0.001 | 0.70 | 0.47 | -0.23 |
| cg02723291 | -0.23 | -17.7 | <0.001 | <0.001 | 0.83 | 0.60 | -0.23 |
| cg25075794 | 0.25 | 17.7 | <0.001 | <0.001 | 0.15 | 0.40 | 0.25 |
| cg16861241 | -0.21 | -17.7 | <0.001 | <0.001 | 0.75 | 0.53 | -0.21 |
| cg13144143 | -0.23 | -17.7 | <0.001 | <0.001 | 0.78 | 0.55 | -0.23 |
| cg10458494 | -0.23 | -17.6 | <0.001 | <0.001 | 0.78 | 0.55 | -0.23 |
| cg21213593 | 0.26 | 17.6 | <0.001 | <0.001 | 0.17 | 0.43 | 0.26 |
| cg25228995 | -0.21 | -17.6 | <0.001 | <0.001 | 0.95 | 0.74 | -0.21 |
| cg10357060 | -0.24 | -17.6 | <0.001 | <0.001 | 0.76 | 0.52 | -0.24 |
| cg08843902 | -0.20 | -17.6 | <0.001 | <0.001 | 0.75 | 0.55 | -0.20 |
| cg20337028 | -0.22 | -17.6 | <0.001 | <0.001 | 0.73 | 0.51 | -0.22 |
| cg22058452 | -0.28 | -17.6 | <0.001 | <0.001 | 0.81 | 0.53 | -0.28 |
| cg22720790 | -0.27 | -17.6 | <0.001 | <0.001 | 0.67 | 0.40 | -0.27 |
| cg26983744 | -0.20 | -17.6 | <0.001 | <0.001 | 0.81 | 0.60 | -0.20 |
| cg11182874 | -0.26 | -17.6 | <0.001 | <0.001 | 0.79 | 0.53 | -0.26 |
| cg14546128 | -0.31 | -17.6 | <0.001 | <0.001 | 0.89 | 0.59 | -0.31 |
| cg05794556 | -0.20 | -17.6 | <0.001 | <0.001 | 0.75 | 0.55 | -0.20 |
| cg09408937 | -0.22 | -17.5 | <0.001 | <0.001 | 0.75 | 0.53 | -0.22 |
| cg13829089 | -0.30 | -17.5 | <0.001 | <0.001 | 0.65 | 0.34 | -0.30 |
| cg14652095 | -0.21 | -17.5 | <0.001 | <0.001 | 0.63 | 0.42 | -0.21 |
| cg24628744 | -0.21 | -17.5 | <0.001 | <0.001 | 0.62 | 0.42 | -0.21 |
| cg15215348 | -0.33 | -17.5 | <0.001 | <0.001 | 0.66 | 0.33 | -0.33 |
| cg01359274 | -0.23 | -17.5 | <0.001 | <0.001 | 0.83 | 0.60 | -0.23 |
| cg15169829 | -0.26 | -17.5 | <0.001 | <0.001 | 0.62 | 0.37 | -0.26 |
| cg21870668 | -0.22 | -17.5 | <0.001 | <0.001 | 0.68 | 0.46 | -0.22 |
| cg13116789 | -0.21 | -17.5 | <0.001 | <0.001 | 0.66 | 0.45 | -0.21 |
| cg21139003 | -0.21 | -17.5 | <0.001 | <0.001 | 0.66 | 0.46 | -0.21 |
| cg19375854 | -0.25 | -17.5 | <0.001 | <0.001 | 0.81 | 0.56 | -0.25 |
| cg00324128 | -0.22 | -17.5 | <0.001 | <0.001 | 0.76 | 0.54 | -0.22 |
| cg05745631 | -0.32 | -17.4 | <0.001 | <0.001 | 0.63 | 0.31 | -0.32 |
| cg03969515 | -0.25 | -17.4 | <0.001 | <0.001 | 0.65 | 0.40 | -0.25 |
| cg15958715 | -0.22 | -17.4 | <0.001 | <0.001 | 0.69 | 0.46 | -0.22 |
| cg11233163 | -0.22 | -17.4 | <0.001 | <0.001 | 0.63 | 0.40 | -0.22 |
| cg23014425 | -0.22 | -17.4 | <0.001 | <0.001 | 0.93 | 0.70 | -0.22 |
| cg00886669 | -0.22 | -17.4 | <0.001 | <0.001 | 0.84 | 0.62 | -0.22 |
| cg19010490 | -0.23 | -17.4 | <0.001 | <0.001 | 0.65 | 0.42 | -0.23 |
| cg08943180 | -0.24 | -17.4 | <0.001 | <0.001 | 0.67 | 0.43 | -0.24 |
| cg07788437 | -0.28 | -17.4 | <0.001 | <0.001 | 0.62 | 0.34 | -0.28 |
| cg13609544 | -0.22 | -17.4 | <0.001 | <0.001 | 0.87 | 0.65 | -0.22 |
| cg16625671 | -0.21 | -17.4 | <0.001 | <0.001 | 0.73 | 0.53 | -0.21 |
| cg23605961 | -0.25 | -17.4 | <0.001 | <0.001 | 0.62 | 0.36 | -0.25 |
| cg16326902 | -0.21 | -17.4 | <0.001 | <0.001 | 0.74 | 0.53 | -0.21 |
| cg14743812 | 0.22 | 17.4 | <0.001 | <0.001 | 0.20 | 0.42 | 0.22 |
| cg11828163 | -0.21 | -17.4 | <0.001 | <0.001 | 0.83 | 0.63 | -0.21 |
| cg17493885 | -0.32 | -17.4 | <0.001 | <0.001 | 0.61 | 0.28 | -0.32 |
| cg21385746 | -0.30 | -17.4 | <0.001 | <0.001 | 0.69 | 0.39 | -0.30 |
| cg02749463 | -0.26 | -17.4 | <0.001 | <0.001 | 0.70 | 0.43 | -0.26 |
| cg27123351 | -0.23 | -17.4 | <0.001 | <0.001 | 0.66 | 0.43 | -0.23 |
| cg26923862 | -0.20 | -17.3 | <0.001 | <0.001 | 0.95 | 0.74 | -0.20 |
| cg11043002 | -0.23 | -17.3 | <0.001 | <0.001 | 0.78 | 0.55 | -0.23 |
| cg16235582 | -0.22 | -17.3 | <0.001 | <0.001 | 0.61 | 0.39 | -0.22 |
| cg22053945 | -0.25 | -17.3 | <0.001 | <0.001 | 0.85 | 0.61 | -0.25 |
| cg14652773 | -0.23 | -17.3 | <0.001 | <0.001 | 0.76 | 0.53 | -0.23 |
| cg23297477 | 0.27 | 17.3 | <0.001 | <0.001 | 0.13 | 0.40 | 0.27 |
| cg12057563 | -0.29 | -17.3 | <0.001 | <0.001 | 0.65 | 0.36 | -0.29 |
| cg19636302 | -0.25 | -17.3 | <0.001 | <0.001 | 0.68 | 0.42 | -0.25 |
| cg04025970 | -0.23 | -17.3 | <0.001 | <0.001 | 0.75 | 0.52 | -0.23 |
| cg05353884 | -0.27 | -17.2 | <0.001 | <0.001 | 0.71 | 0.44 | -0.27 |
| cg19376973 | -0.21 | -17.2 | <0.001 | <0.001 | 0.83 | 0.63 | -0.21 |
| cg10009968 | -0.34 | -17.2 | <0.001 | <0.001 | 0.66 | 0.32 | -0.34 |
| cg04385523 | -0.21 | -17.2 | <0.001 | <0.001 | 0.80 | 0.59 | -0.21 |
| cg10531918 | -0.20 | -17.2 | <0.001 | <0.001 | 0.64 | 0.44 | -0.20 |
| cg22786811 | -0.23 | -17.2 | <0.001 | <0.001 | 0.63 | 0.40 | -0.23 |
| cg18596947 | -0.22 | -17.2 | <0.001 | <0.001 | 0.83 | 0.60 | -0.22 |
| cg18757468 | -0.27 | -17.2 | <0.001 | <0.001 | 0.66 | 0.39 | -0.27 |
| cg22690322 | -0.22 | -17.2 | <0.001 | <0.001 | 0.86 | 0.64 | -0.22 |
| cg08637446 | -0.21 | -17.2 | <0.001 | <0.001 | 0.79 | 0.58 | -0.21 |
| cg13939055 | -0.24 | -17.1 | <0.001 | <0.001 | 0.60 | 0.37 | -0.24 |
| cg15651925 | -0.28 | -17.1 | <0.001 | <0.001 | 0.67 | 0.39 | -0.28 |
| cg24840099 | -0.24 | -17.1 | <0.001 | <0.001 | 0.67 | 0.43 | -0.24 |
| cg21221690 | -0.21 | -17.1 | <0.001 | <0.001 | 0.79 | 0.58 | -0.21 |
| cg25750507 | -0.23 | -17.1 | <0.001 | <0.001 | 0.83 | 0.60 | -0.23 |
| cg23389215 | -0.21 | -17.1 | <0.001 | <0.001 | 0.65 | 0.44 | -0.21 |
| cg25356456 | -0.22 | -17.1 | <0.001 | <0.001 | 0.64 | 0.42 | -0.22 |
| cg13762244 | -0.25 | -17.1 | <0.001 | <0.001 | 0.68 | 0.43 | -0.25 |
| cg13960999 | -0.24 | -17.1 | <0.001 | <0.001 | 0.68 | 0.44 | -0.24 |
| cg26638341 | -0.25 | -17.1 | <0.001 | <0.001 | 0.68 | 0.43 | -0.25 |
| cg11610925 | -0.30 | -17.1 | <0.001 | <0.001 | 0.71 | 0.41 | -0.30 |
| cg15077792 | -0.23 | -17.1 | <0.001 | <0.001 | 0.70 | 0.47 | -0.23 |
| cg23931049 | -0.26 | -17.1 | <0.001 | <0.001 | 0.69 | 0.43 | -0.26 |
| cg03185655 | -0.20 | -17.1 | <0.001 | <0.001 | 0.83 | 0.63 | -0.20 |
| cg21089930 | -0.21 | -17.1 | <0.001 | <0.001 | 0.63 | 0.42 | -0.21 |
| cg18492926 | -0.20 | -17.0 | <0.001 | <0.001 | 0.73 | 0.53 | -0.20 |
| cg13492337 | -0.20 | -17.0 | <0.001 | <0.001 | 0.75 | 0.55 | -0.20 |
| cg18883033 | -0.22 | -17.0 | <0.001 | <0.001 | 0.71 | 0.49 | -0.22 |
| cg22521151 | -0.21 | -17.0 | <0.001 | <0.001 | 0.75 | 0.54 | -0.21 |
| cg06814287 | -0.26 | -17.0 | <0.001 | <0.001 | 0.69 | 0.43 | -0.26 |
| cg18473521 | -0.22 | -17.0 | <0.001 | <0.001 | 0.75 | 0.52 | -0.22 |
| cg12937186 | -0.27 | -17.0 | <0.001 | <0.001 | 0.75 | 0.48 | -0.27 |
| cg10057940 | -0.24 | -17.0 | <0.001 | <0.001 | 0.62 | 0.38 | -0.24 |
| cg02546477 | -0.26 | -17.0 | <0.001 | <0.001 | 0.63 | 0.36 | -0.26 |
| cg13750214 | -0.25 | -17.0 | <0.001 | <0.001 | 0.73 | 0.48 | -0.25 |
| cg01157780 | -0.25 | -16.9 | <0.001 | <0.001 | 0.68 | 0.42 | -0.25 |
| cg03479211 | -0.23 | -16.9 | <0.001 | <0.001 | 0.69 | 0.46 | -0.23 |
| cg14994521 | -0.23 | -16.9 | <0.001 | <0.001 | 0.90 | 0.67 | -0.23 |
| cg14785392 | -0.24 | -16.9 | <0.001 | <0.001 | 0.74 | 0.49 | -0.24 |
| cg13679679 | -0.23 | -16.9 | <0.001 | <0.001 | 0.60 | 0.37 | -0.23 |
| cg20365336 | 0.21 | 16.9 | <0.001 | <0.001 | 0.19 | 0.40 | 0.21 |
| cg12213811 | -0.23 | -16.9 | <0.001 | <0.001 | 0.74 | 0.51 | -0.23 |
| cg03561416 | -0.22 | -16.9 | <0.001 | <0.001 | 0.76 | 0.54 | -0.22 |
| cg23003225 | -0.20 | -16.9 | <0.001 | <0.001 | 0.80 | 0.60 | -0.20 |
| cg06312072 | -0.23 | -16.9 | <0.001 | <0.001 | 0.75 | 0.52 | -0.23 |
| cg14214165 | -0.22 | -16.9 | <0.001 | <0.001 | 0.62 | 0.40 | -0.22 |
| cg10396171 | -0.25 | -16.9 | <0.001 | <0.001 | 0.78 | 0.53 | -0.25 |
| cg24485696 | -0.28 | -16.9 | <0.001 | <0.001 | 0.64 | 0.36 | -0.28 |
| cg07665510 | 0.33 | 16.9 | <0.001 | <0.001 | 0.18 | 0.51 | 0.33 |
| cg20373544 | -0.25 | -16.8 | <0.001 | <0.001 | 0.80 | 0.56 | -0.25 |
| cg15452135 | -0.21 | -16.8 | <0.001 | <0.001 | 0.83 | 0.62 | -0.21 |
| cg16190209 | -0.21 | -16.8 | <0.001 | <0.001 | 0.92 | 0.71 | -0.21 |
| cg00254258 | -0.26 | -16.8 | <0.001 | <0.001 | 0.75 | 0.49 | -0.26 |
| cg26947448 | -0.22 | -16.8 | <0.001 | <0.001 | 0.68 | 0.47 | -0.22 |
| cg26232247 | 0.24 | 16.8 | <0.001 | <0.001 | 0.13 | 0.37 | 0.24 |
| cg17777592 | -0.26 | -16.8 | <0.001 | <0.001 | 0.76 | 0.50 | -0.26 |
| cg25138553 | -0.26 | -16.8 | <0.001 | <0.001 | 0.61 | 0.35 | -0.26 |
| cg01227246 | -0.22 | -16.8 | <0.001 | <0.001 | 0.80 | 0.57 | -0.22 |
| cg04850999 | -0.22 | -16.8 | <0.001 | <0.001 | 0.74 | 0.52 | -0.22 |
| cg12860635 | -0.21 | -16.8 | <0.001 | <0.001 | 0.70 | 0.50 | -0.21 |
| cg09480190 | -0.31 | -16.8 | <0.001 | <0.001 | 0.75 | 0.44 | -0.31 |
| cg20686446 | -0.24 | -16.8 | <0.001 | <0.001 | 0.84 | 0.60 | -0.24 |
| cg08481464 | -0.23 | -16.8 | <0.001 | <0.001 | 0.79 | 0.55 | -0.23 |
| cg16481280 | -0.30 | -16.7 | <0.001 | <0.001 | 0.64 | 0.34 | -0.30 |
| cg08972170 | -0.25 | -16.7 | <0.001 | <0.001 | 0.78 | 0.53 | -0.25 |
| cg14848772 | 0.25 | 16.7 | <0.001 | <0.001 | 0.18 | 0.43 | 0.25 |
| cg06686742 | -0.22 | -16.7 | <0.001 | <0.001 | 0.76 | 0.53 | -0.22 |
| cg14398228 | -0.23 | -16.7 | <0.001 | <0.001 | 0.68 | 0.45 | -0.23 |
| cg16723800 | -0.31 | -16.7 | <0.001 | <0.001 | 0.71 | 0.40 | -0.31 |
| cg24454741 | -0.23 | -16.7 | <0.001 | <0.001 | 0.60 | 0.37 | -0.23 |
| cg00740389 | -0.23 | -16.7 | <0.001 | <0.001 | 0.83 | 0.59 | -0.23 |
| cg05209917 | -0.24 | -16.7 | <0.001 | <0.001 | 0.65 | 0.40 | -0.24 |
| cg13931285 | -0.24 | -16.7 | <0.001 | <0.001 | 0.70 | 0.45 | -0.24 |
| cg22221831 | -0.29 | -16.7 | <0.001 | <0.001 | 0.69 | 0.40 | -0.29 |
| cg13475578 | -0.22 | -16.6 | <0.001 | <0.001 | 0.70 | 0.48 | -0.22 |
| cg04372674 | 0.23 | 16.6 | <0.001 | <0.001 | 0.16 | 0.39 | 0.23 |
| cg16045897 | -0.20 | -16.6 | <0.001 | <0.001 | 0.83 | 0.63 | -0.20 |
| cg09038266 | -0.21 | -16.6 | <0.001 | <0.001 | 0.80 | 0.59 | -0.21 |
| cg03819089 | -0.23 | -16.6 | <0.001 | <0.001 | 0.63 | 0.40 | -0.23 |
| cg01093854 | -0.22 | -16.6 | <0.001 | <0.001 | 0.84 | 0.61 | -0.22 |
| cg00584026 | -0.28 | -16.6 | <0.001 | <0.001 | 0.82 | 0.54 | -0.28 |
| cg02067022 | -0.22 | -16.6 | <0.001 | <0.001 | 0.67 | 0.46 | -0.22 |
| cg25830307 | -0.28 | -16.6 | <0.001 | <0.001 | 0.87 | 0.59 | -0.28 |
| cg10621809 | -0.24 | -16.6 | <0.001 | <0.001 | 0.61 | 0.36 | -0.24 |
| cg09238868 | -0.21 | -16.6 | <0.001 | <0.001 | 0.69 | 0.47 | -0.21 |
| cg24738346 | -0.23 | -16.6 | <0.001 | <0.001 | 0.72 | 0.49 | -0.23 |
| cg05012239 | -0.24 | -16.5 | <0.001 | <0.001 | 0.81 | 0.57 | -0.24 |
| cg18213495 | -0.22 | -16.5 | <0.001 | <0.001 | 0.71 | 0.49 | -0.22 |
| cg19343034 | -0.21 | -16.5 | <0.001 | <0.001 | 0.89 | 0.68 | -0.21 |
| cg19592637 | -0.23 | -16.5 | <0.001 | <0.001 | 0.62 | 0.39 | -0.23 |
| cg25533993 | -0.21 | -16.5 | <0.001 | <0.001 | 0.72 | 0.50 | -0.21 |
| cg14385362 | 0.20 | 16.5 | <0.001 | <0.001 | 0.16 | 0.36 | 0.20 |
| cg19741660 | -0.22 | -16.5 | <0.001 | <0.001 | 0.71 | 0.49 | -0.22 |
| cg23520688 | -0.25 | -16.5 | <0.001 | <0.001 | 0.72 | 0.47 | -0.25 |
| cg00489772 | -0.23 | -16.5 | <0.001 | <0.001 | 0.62 | 0.39 | -0.23 |
| cg21105318 | -0.25 | -16.5 | <0.001 | <0.001 | 0.78 | 0.53 | -0.25 |
| cg09173768 | -0.22 | -16.5 | <0.001 | <0.001 | 0.76 | 0.54 | -0.22 |
| cg20174393 | -0.20 | -16.5 | <0.001 | <0.001 | 0.84 | 0.64 | -0.20 |
| cg04965934 | -0.24 | -16.5 | <0.001 | <0.001 | 0.68 | 0.44 | -0.24 |
| cg26764345 | -0.22 | -16.5 | <0.001 | <0.001 | 0.88 | 0.66 | -0.22 |
| cg08298946 | -0.23 | -16.5 | <0.001 | <0.001 | 0.68 | 0.44 | -0.23 |
| cg04689178 | -0.23 | -16.5 | <0.001 | <0.001 | 0.78 | 0.55 | -0.23 |
| cg27456203 | -0.32 | -16.5 | <0.001 | <0.001 | 0.65 | 0.33 | -0.32 |
| cg25461801 | -0.24 | -16.5 | <0.001 | <0.001 | 0.64 | 0.40 | -0.24 |
| cg14040679 | -0.21 | -16.5 | <0.001 | <0.001 | 0.71 | 0.49 | -0.21 |
| cg24997744 | -0.22 | -16.5 | <0.001 | <0.001 | 0.90 | 0.68 | -0.22 |
| cg06564875 | -0.30 | -16.5 | <0.001 | <0.001 | 0.63 | 0.33 | -0.30 |
| cg24818200 | -0.24 | -16.5 | <0.001 | <0.001 | 0.66 | 0.42 | -0.24 |
| cg04772968 | -0.20 | -16.4 | <0.001 | <0.001 | 0.73 | 0.53 | -0.20 |
| cg06395298 | -0.23 | -16.4 | <0.001 | <0.001 | 0.84 | 0.61 | -0.23 |
| cg20657864 | -0.21 | -16.4 | <0.001 | <0.001 | 0.87 | 0.66 | -0.21 |
| cg27389454 | -0.23 | -16.4 | <0.001 | <0.001 | 0.65 | 0.43 | -0.23 |
| cg13812230 | -0.25 | -16.4 | <0.001 | <0.001 | 0.63 | 0.37 | -0.25 |
| cg03318906 | -0.22 | -16.4 | <0.001 | <0.001 | 0.75 | 0.54 | -0.22 |
| cg06251500 | -0.20 | -16.4 | <0.001 | <0.001 | 0.75 | 0.54 | -0.20 |
| cg18320766 | 0.20 | 16.4 | <0.001 | <0.001 | 0.06 | 0.26 | 0.20 |
| cg13346441 | -0.27 | -16.4 | <0.001 | <0.001 | 0.78 | 0.51 | -0.27 |
| cg16342949 | -0.24 | -16.4 | <0.001 | <0.001 | 0.74 | 0.50 | -0.24 |
| cg18703066 | -0.21 | -16.4 | <0.001 | <0.001 | 0.73 | 0.52 | -0.21 |
| cg15613420 | -0.27 | -16.4 | <0.001 | <0.001 | 0.72 | 0.45 | -0.27 |
| cg24366168 | -0.22 | -16.4 | <0.001 | <0.001 | 0.74 | 0.52 | -0.22 |
| cg25892587 | -0.22 | -16.4 | <0.001 | <0.001 | 0.73 | 0.51 | -0.22 |
| cg09595185 | -0.24 | -16.4 | <0.001 | <0.001 | 0.64 | 0.40 | -0.24 |
| cg24631526 | 0.27 | 16.4 | <0.001 | <0.001 | 0.14 | 0.41 | 0.27 |
| cg11100933 | 0.20 | 16.4 | <0.001 | <0.001 | 0.17 | 0.37 | 0.20 |
| cg17647091 | -0.25 | -16.4 | <0.001 | <0.001 | 0.65 | 0.40 | -0.25 |
| cg03854238 | -0.28 | -16.4 | <0.001 | <0.001 | 0.65 | 0.36 | -0.28 |
| cg18788664 | -0.30 | -16.4 | <0.001 | <0.001 | 0.62 | 0.32 | -0.30 |
| cg16563255 | -0.22 | -16.4 | <0.001 | <0.001 | 0.65 | 0.43 | -0.22 |
| cg16204205 | -0.21 | -16.3 | <0.001 | <0.001 | 0.77 | 0.56 | -0.21 |
| cg01747796 | -0.25 | -16.3 | <0.001 | <0.001 | 0.87 | 0.62 | -0.25 |
| cg24435747 | -0.31 | -16.3 | <0.001 | <0.001 | 0.63 | 0.32 | -0.31 |
| cg06634717 | -0.26 | -16.3 | <0.001 | <0.001 | 0.84 | 0.57 | -0.26 |
| cg05664938 | -0.20 | -16.3 | <0.001 | <0.001 | 0.89 | 0.69 | -0.20 |
| cg21815337 | -0.22 | -16.3 | <0.001 | <0.001 | 0.74 | 0.53 | -0.22 |
| cg11286742 | -0.23 | -16.3 | <0.001 | <0.001 | 0.83 | 0.60 | -0.23 |
| cg12639429 | -0.22 | -16.3 | <0.001 | <0.001 | 0.79 | 0.56 | -0.22 |
| cg13725340 | -0.27 | -16.3 | <0.001 | <0.001 | 0.73 | 0.46 | -0.27 |
| cg26511075 | -0.26 | -16.3 | <0.001 | <0.001 | 0.73 | 0.46 | -0.26 |
| cg16408593 | -0.22 | -16.3 | <0.001 | <0.001 | 0.76 | 0.55 | -0.22 |
| cg06383135 | -0.24 | -16.3 | <0.001 | <0.001 | 0.72 | 0.48 | -0.24 |
| cg10859192 | -0.21 | -16.3 | <0.001 | <0.001 | 0.82 | 0.61 | -0.21 |
| cg07896068 | -0.26 | -16.3 | <0.001 | <0.001 | 0.72 | 0.46 | -0.26 |
| cg19149132 | -0.29 | -16.3 | <0.001 | <0.001 | 0.70 | 0.41 | -0.29 |
| cg17963461 | -0.22 | -16.2 | <0.001 | <0.001 | 0.82 | 0.60 | -0.22 |
| cg08316831 | -0.23 | -16.2 | <0.001 | <0.001 | 0.77 | 0.54 | -0.23 |
| cg15792134 | -0.30 | -16.2 | <0.001 | <0.001 | 0.81 | 0.52 | -0.30 |
| cg13471209 | -0.22 | -16.2 | <0.001 | <0.001 | 0.81 | 0.59 | -0.22 |
| cg13614409 | -0.29 | -16.2 | <0.001 | <0.001 | 0.61 | 0.33 | -0.29 |
| cg01466348 | -0.22 | -16.2 | <0.001 | <0.001 | 0.82 | 0.60 | -0.22 |
| cg23553912 | -0.23 | -16.2 | <0.001 | <0.001 | 0.65 | 0.42 | -0.23 |
| cg27119612 | -0.22 | -16.2 | <0.001 | <0.001 | 0.68 | 0.47 | -0.22 |
| cg11696200 | -0.29 | -16.2 | <0.001 | <0.001 | 0.72 | 0.43 | -0.29 |
| cg19735250 | -0.26 | -16.2 | <0.001 | <0.001 | 0.66 | 0.39 | -0.26 |
| cg27065717 | -0.22 | -16.1 | <0.001 | <0.001 | 0.70 | 0.48 | -0.22 |
| cg21157873 | -0.26 | -16.1 | <0.001 | <0.001 | 0.63 | 0.37 | -0.26 |
| cg25930644 | -0.20 | -16.1 | <0.001 | <0.001 | 0.72 | 0.52 | -0.20 |
| cg20785796 | -0.22 | -16.1 | <0.001 | <0.001 | 0.67 | 0.45 | -0.22 |
| cg08971637 | -0.21 | -16.1 | <0.001 | <0.001 | 0.79 | 0.58 | -0.21 |
| cg26556008 | -0.24 | -16.1 | <0.001 | <0.001 | 0.72 | 0.48 | -0.24 |
| cg08474826 | -0.22 | -16.1 | <0.001 | <0.001 | 0.65 | 0.43 | -0.22 |
| cg18165065 | -0.24 | -16.1 | <0.001 | <0.001 | 0.77 | 0.53 | -0.24 |
| cg10484211 | -0.25 | -16.1 | <0.001 | <0.001 | 0.67 | 0.41 | -0.25 |
| cg25497530 | -0.21 | -16.1 | <0.001 | <0.001 | 0.83 | 0.62 | -0.21 |
| cg00480115 | -0.20 | -16.1 | <0.001 | <0.001 | 0.86 | 0.66 | -0.20 |
| cg05279513 | -0.25 | -16.1 | <0.001 | <0.001 | 0.67 | 0.43 | -0.25 |
| cg21864868 | -0.21 | -16.0 | <0.001 | <0.001 | 0.74 | 0.53 | -0.21 |
| cg23886165 | -0.23 | -16.0 | <0.001 | <0.001 | 0.76 | 0.52 | -0.23 |
| cg15871215 | -0.25 | -16.0 | <0.001 | <0.001 | 0.63 | 0.38 | -0.25 |
| cg21498785 | -0.23 | -16.0 | <0.001 | <0.001 | 0.75 | 0.52 | -0.23 |
| cg16574155 | -0.21 | -16.0 | <0.001 | <0.001 | 0.78 | 0.57 | -0.21 |
| cg06516502 | -0.32 | -16.0 | <0.001 | <0.001 | 0.65 | 0.33 | -0.32 |
| cg02879438 | -0.21 | -16.0 | <0.001 | <0.001 | 0.93 | 0.71 | -0.21 |
| cg02152631 | -0.27 | -16.0 | <0.001 | <0.001 | 0.64 | 0.38 | -0.27 |
| cg24091104 | -0.23 | -16.0 | <0.001 | <0.001 | 0.83 | 0.60 | -0.23 |
| cg11217960 | -0.23 | -16.0 | <0.001 | <0.001 | 0.63 | 0.40 | -0.23 |
| cg26344026 | -0.22 | -16.0 | <0.001 | <0.001 | 0.76 | 0.54 | -0.22 |
| cg07123182 | -0.21 | -16.0 | <0.001 | <0.001 | 0.61 | 0.40 | -0.21 |
| cg18121224 | -0.26 | -16.0 | <0.001 | <0.001 | 0.64 | 0.39 | -0.26 |
| cg10504000 | 0.21 | 16.0 | <0.001 | <0.001 | 0.19 | 0.39 | 0.21 |
| cg03429569 | -0.22 | -16.0 | <0.001 | <0.001 | 0.66 | 0.44 | -0.22 |
| cg23781276 | -0.22 | -16.0 | <0.001 | <0.001 | 0.70 | 0.48 | -0.22 |
| cg11070172 | -0.21 | -15.9 | <0.001 | <0.001 | 0.88 | 0.67 | -0.21 |
| cg01171339 | -0.23 | -15.9 | <0.001 | <0.001 | 0.64 | 0.41 | -0.23 |
| cg04194947 | -0.22 | -15.9 | <0.001 | <0.001 | 0.80 | 0.58 | -0.22 |
| cg00061185 | -0.21 | -15.9 | <0.001 | <0.001 | 0.91 | 0.70 | -0.21 |
| cg19303187 | 0.21 | 15.9 | <0.001 | <0.001 | 0.17 | 0.38 | 0.21 |
| cg11826295 | -0.22 | -15.9 | <0.001 | <0.001 | 0.71 | 0.49 | -0.22 |
| cg17547742 | -0.30 | -15.9 | <0.001 | <0.001 | 0.63 | 0.34 | -0.30 |
| cg25334934 | -0.30 | -15.9 | <0.001 | <0.001 | 0.73 | 0.43 | -0.30 |
| cg12416856 | -0.20 | -15.9 | <0.001 | <0.001 | 0.81 | 0.61 | -0.20 |
| cg17542408 | -0.24 | -15.9 | <0.001 | <0.001 | 0.61 | 0.37 | -0.24 |
| cg19894382 | -0.20 | -15.8 | <0.001 | <0.001 | 0.66 | 0.45 | -0.20 |
| cg06066587 | -0.27 | -15.8 | <0.001 | <0.001 | 0.75 | 0.48 | -0.27 |
| cg16316394 | -0.20 | -15.8 | <0.001 | <0.001 | 0.70 | 0.49 | -0.20 |
| cg14633742 | -0.25 | -15.8 | <0.001 | <0.001 | 0.69 | 0.45 | -0.25 |
| cg19005446 | -0.24 | -15.8 | <0.001 | <0.001 | 0.62 | 0.38 | -0.24 |
| cg25032595 | -0.24 | -15.8 | <0.001 | <0.001 | 0.60 | 0.36 | -0.24 |
| cg07904475 | -0.23 | -15.8 | <0.001 | <0.001 | 0.70 | 0.47 | -0.23 |
| cg10802005 | -0.21 | -15.8 | <0.001 | <0.001 | 0.74 | 0.53 | -0.21 |
| cg05107535 | -0.24 | -15.8 | <0.001 | <0.001 | 0.64 | 0.41 | -0.24 |
| cg00936626 | -0.20 | -15.8 | <0.001 | <0.001 | 0.63 | 0.43 | -0.20 |
| cg10274108 | -0.21 | -15.8 | <0.001 | <0.001 | 0.63 | 0.42 | -0.21 |
| cg08926365 | -0.25 | -15.8 | <0.001 | <0.001 | 0.63 | 0.38 | -0.25 |
| cg20928429 | -0.21 | -15.7 | <0.001 | <0.001 | 0.78 | 0.57 | -0.21 |
| cg04036329 | -0.21 | -15.7 | <0.001 | <0.001 | 0.63 | 0.42 | -0.21 |
| cg10775039 | -0.20 | -15.7 | <0.001 | <0.001 | 0.80 | 0.60 | -0.20 |
| cg22507154 | -0.22 | -15.7 | <0.001 | <0.001 | 0.69 | 0.47 | -0.22 |
| cg02495743 | 0.22 | 15.7 | <0.001 | <0.001 | 0.18 | 0.40 | 0.22 |
| cg08147813 | -0.24 | -15.7 | <0.001 | <0.001 | 0.63 | 0.39 | -0.24 |
| cg11573170 | -0.20 | -15.7 | <0.001 | <0.001 | 0.80 | 0.60 | -0.20 |
| cg16822666 | -0.20 | -15.7 | <0.001 | <0.001 | 0.62 | 0.42 | -0.20 |
| cg19066391 | -0.20 | -15.7 | <0.001 | <0.001 | 0.63 | 0.43 | -0.20 |
| cg07212702 | -0.20 | -15.7 | <0.001 | <0.001 | 0.71 | 0.51 | -0.20 |
| cg09479341 | -0.20 | -15.7 | <0.001 | <0.001 | 0.71 | 0.51 | -0.20 |
| cg21490444 | -0.24 | -15.7 | <0.001 | <0.001 | 0.74 | 0.50 | -0.24 |
| cg21961149 | -0.26 | -15.7 | <0.001 | <0.001 | 0.68 | 0.42 | -0.26 |
| cg12417775 | -0.22 | -15.7 | <0.001 | <0.001 | 0.66 | 0.44 | -0.22 |
| cg16294280 | -0.22 | -15.7 | <0.001 | <0.001 | 0.61 | 0.39 | -0.22 |
| cg19502812 | -0.21 | -15.7 | <0.001 | <0.001 | 0.80 | 0.60 | -0.21 |
| cg10658779 | -0.22 | -15.7 | <0.001 | <0.001 | 0.82 | 0.60 | -0.22 |
| cg15205441 | -0.23 | -15.6 | <0.001 | <0.001 | 0.80 | 0.57 | -0.23 |
| cg08090385 | -0.21 | -15.6 | <0.001 | <0.001 | 0.74 | 0.52 | -0.21 |
| cg19505458 | -0.26 | -15.6 | <0.001 | <0.001 | 0.63 | 0.36 | -0.26 |
| cg20095851 | -0.23 | -15.6 | <0.001 | <0.001 | 0.81 | 0.58 | -0.23 |
| cg14610217 | -0.21 | -15.6 | <0.001 | <0.001 | 0.63 | 0.43 | -0.21 |
| cg02642822 | -0.22 | -15.6 | <0.001 | <0.001 | 0.62 | 0.40 | -0.22 |
| cg19698340 | -0.20 | -15.6 | <0.001 | <0.001 | 0.75 | 0.55 | -0.20 |
| cg21771679 | -0.21 | -15.6 | <0.001 | <0.001 | 0.66 | 0.45 | -0.21 |
| cg06207120 | -0.21 | -15.6 | <0.001 | <0.001 | 0.64 | 0.43 | -0.21 |
| cg00891611 | -0.21 | -15.6 | <0.001 | <0.001 | 0.66 | 0.45 | -0.21 |
| cg11520719 | -0.22 | -15.6 | <0.001 | <0.001 | 0.68 | 0.46 | -0.22 |
| cg02388453 | -0.25 | -15.6 | <0.001 | <0.001 | 0.79 | 0.54 | -0.25 |
| cg27109043 | -0.24 | -15.6 | <0.001 | <0.001 | 0.71 | 0.47 | -0.24 |
| cg25541653 | -0.25 | -15.5 | <0.001 | <0.001 | 0.70 | 0.45 | -0.25 |
| cg20152430 | -0.25 | -15.5 | <0.001 | <0.001 | 0.75 | 0.50 | -0.25 |
| cg13295262 | -0.22 | -15.5 | <0.001 | <0.001 | 0.65 | 0.43 | -0.22 |
| cg00604771 | -0.20 | -15.5 | <0.001 | <0.001 | 0.75 | 0.55 | -0.20 |
| cg15428140 | -0.24 | -15.5 | <0.001 | <0.001 | 0.61 | 0.37 | -0.24 |
| cg07654588 | -0.20 | -15.5 | <0.001 | <0.001 | 0.65 | 0.44 | -0.20 |
| cg02616418 | -0.26 | -15.5 | <0.001 | <0.001 | 0.61 | 0.36 | -0.26 |
| cg22676516 | -0.22 | -15.5 | <0.001 | <0.001 | 0.62 | 0.41 | -0.22 |
| cg00395579 | -0.22 | -15.5 | <0.001 | <0.001 | 0.63 | 0.40 | -0.22 |
| cg05564086 | 0.20 | 15.5 | <0.001 | <0.001 | 0.08 | 0.28 | 0.20 |
| cg03773198 | -0.20 | -15.5 | <0.001 | <0.001 | 0.64 | 0.44 | -0.20 |
| cg01175550 | -0.22 | -15.5 | <0.001 | <0.001 | 0.75 | 0.54 | -0.22 |
| cg13996750 | -0.23 | -15.5 | <0.001 | <0.001 | 0.62 | 0.39 | -0.23 |
| cg03734783 | -0.20 | -15.5 | <0.001 | <0.001 | 0.70 | 0.50 | -0.20 |
| cg00412554 | -0.27 | -15.5 | <0.001 | <0.001 | 0.66 | 0.39 | -0.27 |
| cg22238122 | -0.26 | -15.5 | <0.001 | <0.001 | 0.67 | 0.41 | -0.26 |
| cg21033440 | -0.26 | -15.5 | <0.001 | <0.001 | 0.63 | 0.37 | -0.26 |
| cg01205935 | -0.24 | -15.5 | <0.001 | <0.001 | 0.65 | 0.42 | -0.24 |
| cg11613427 | -0.20 | -15.5 | <0.001 | <0.001 | 0.77 | 0.57 | -0.20 |
| cg08222513 | -0.27 | -15.4 | <0.001 | <0.001 | 0.63 | 0.36 | -0.27 |
| cg10230442 | -0.23 | -15.4 | <0.001 | <0.001 | 0.72 | 0.49 | -0.23 |
| cg14466759 | -0.20 | -15.4 | <0.001 | <0.001 | 0.81 | 0.61 | -0.20 |
| cg07044282 | -0.22 | -15.4 | <0.001 | <0.001 | 0.75 | 0.53 | -0.22 |
| cg12768523 | -0.22 | -15.4 | <0.001 | <0.001 | 0.69 | 0.47 | -0.22 |
| cg23226168 | -0.27 | -15.4 | <0.001 | <0.001 | 0.62 | 0.36 | -0.27 |
| cg13876553 | -0.22 | -15.4 | <0.001 | <0.001 | 0.79 | 0.57 | -0.22 |
| cg05977462 | -0.22 | -15.4 | <0.001 | <0.001 | 0.62 | 0.41 | -0.22 |
| cg20375320 | -0.21 | -15.4 | <0.001 | <0.001 | 0.89 | 0.68 | -0.21 |
| cg12932102 | -0.29 | -15.3 | <0.001 | <0.001 | 0.78 | 0.49 | -0.29 |
| cg19192626 | -0.24 | -15.3 | <0.001 | <0.001 | 0.64 | 0.40 | -0.24 |
| cg09487101 | -0.21 | -15.3 | <0.001 | <0.001 | 0.63 | 0.42 | -0.21 |
| cg14098223 | -0.20 | -15.3 | <0.001 | <0.001 | 0.69 | 0.49 | -0.20 |
| cg07250758 | -0.20 | -15.3 | <0.001 | <0.001 | 0.64 | 0.44 | -0.20 |
| cg15490801 | -0.24 | -15.3 | <0.001 | <0.001 | 0.63 | 0.38 | -0.24 |
| cg04003871 | -0.22 | -15.3 | <0.001 | <0.001 | 0.70 | 0.48 | -0.22 |
| cg05490023 | -0.20 | -15.3 | <0.001 | <0.001 | 0.67 | 0.47 | -0.20 |
| cg24483493 | -0.25 | -15.3 | <0.001 | <0.001 | 0.64 | 0.39 | -0.25 |
| cg08795640 | -0.21 | -15.2 | <0.001 | <0.001 | 0.67 | 0.46 | -0.21 |
| cg17612681 | -0.22 | -15.2 | <0.001 | <0.001 | 0.62 | 0.40 | -0.22 |
| cg10296718 | -0.23 | -15.2 | <0.001 | <0.001 | 0.78 | 0.55 | -0.23 |
| cg25183214 | -0.20 | -15.2 | <0.001 | <0.001 | 0.87 | 0.67 | -0.20 |
| cg15190451 | -0.25 | -15.2 | <0.001 | <0.001 | 0.85 | 0.60 | -0.25 |
| cg14138884 | 0.21 | 15.2 | <0.001 | <0.001 | 0.19 | 0.40 | 0.21 |
| cg27342919 | -0.28 | -15.2 | <0.001 | <0.001 | 0.61 | 0.34 | -0.28 |
| cg01942797 | -0.23 | -15.2 | <0.001 | <0.001 | 0.77 | 0.53 | -0.23 |
| cg26667091 | -0.22 | -15.2 | <0.001 | <0.001 | 0.68 | 0.46 | -0.22 |
| cg19547192 | -0.22 | -15.2 | <0.001 | <0.001 | 0.91 | 0.69 | -0.22 |
| cg00618626 | -0.23 | -15.2 | <0.001 | <0.001 | 0.74 | 0.51 | -0.23 |
| cg09514366 | -0.22 | -15.2 | <0.001 | <0.001 | 0.67 | 0.45 | -0.22 |
| cg18437633 | -0.21 | -15.1 | <0.001 | <0.001 | 0.80 | 0.60 | -0.21 |
| cg18864124 | -0.21 | -15.1 | <0.001 | <0.001 | 0.74 | 0.53 | -0.21 |
| cg21778193 | -0.25 | -15.1 | <0.001 | <0.001 | 0.66 | 0.42 | -0.25 |
| cg16963138 | -0.21 | -15.1 | <0.001 | <0.001 | 0.78 | 0.57 | -0.21 |
| cg10096100 | -0.22 | -15.1 | <0.001 | <0.001 | 0.79 | 0.58 | -0.22 |
| cg06970290 | -0.20 | -15.1 | <0.001 | <0.001 | 0.85 | 0.64 | -0.20 |
| cg10055817 | -0.22 | -15.1 | <0.001 | <0.001 | 0.86 | 0.64 | -0.22 |
| cg03953626 | 0.27 | 15.1 | <0.001 | <0.001 | 0.18 | 0.44 | 0.27 |
| cg13643006 | -0.23 | -15.1 | <0.001 | <0.001 | 0.81 | 0.58 | -0.23 |
| cg01963906 | -0.20 | -15.1 | <0.001 | <0.001 | 0.70 | 0.50 | -0.20 |
| cg05512157 | -0.23 | -15.1 | <0.001 | <0.001 | 0.64 | 0.42 | -0.23 |
| cg15071463 | -0.22 | -15.0 | <0.001 | <0.001 | 0.75 | 0.54 | -0.22 |
| cg06759874 | -0.20 | -15.0 | <0.001 | <0.001 | 0.72 | 0.52 | -0.20 |
| cg01854776 | -0.23 | -15.0 | <0.001 | <0.001 | 0.87 | 0.65 | -0.23 |
| cg25281029 | -0.24 | -15.0 | <0.001 | <0.001 | 0.70 | 0.46 | -0.24 |
| cg02732915 | -0.21 | -15.0 | <0.001 | <0.001 | 0.67 | 0.46 | -0.21 |
| cg02702693 | -0.26 | -15.0 | <0.001 | <0.001 | 0.64 | 0.38 | -0.26 |
| cg25792518 | -0.23 | -15.0 | <0.001 | <0.001 | 0.72 | 0.49 | -0.23 |
| cg23325335 | -0.21 | -15.0 | <0.001 | <0.001 | 0.65 | 0.44 | -0.21 |
| cg14556256 | -0.21 | -15.0 | <0.001 | <0.001 | 0.86 | 0.64 | -0.21 |
| cg06643156 | -0.21 | -15.0 | <0.001 | <0.001 | 0.70 | 0.49 | -0.21 |
| cg18050804 | -0.20 | -15.0 | <0.001 | <0.001 | 0.63 | 0.43 | -0.20 |
| cg15154628 | -0.21 | -15.0 | <0.001 | <0.001 | 0.79 | 0.58 | -0.21 |
| cg24348495 | -0.22 | -14.9 | <0.001 | <0.001 | 0.76 | 0.54 | -0.22 |
| cg01493728 | -0.27 | -14.9 | <0.001 | <0.001 | 0.62 | 0.35 | -0.27 |
| cg04124457 | -0.22 | -14.9 | <0.001 | <0.001 | 0.72 | 0.50 | -0.22 |
| cg19106932 | -0.22 | -14.9 | <0.001 | <0.001 | 0.61 | 0.39 | -0.22 |
| cg06786372 | -0.21 | -14.9 | <0.001 | <0.001 | 0.69 | 0.47 | -0.21 |
| cg01021196 | -0.21 | -14.9 | <0.001 | <0.001 | 0.80 | 0.59 | -0.21 |
| cg12279968 | -0.23 | -14.9 | <0.001 | <0.001 | 0.81 | 0.58 | -0.23 |
| cg02939659 | -0.26 | -14.9 | <0.001 | <0.001 | 0.81 | 0.55 | -0.26 |
| cg18365406 | -0.27 | -14.9 | <0.001 | <0.001 | 0.73 | 0.46 | -0.27 |
| cg19466906 | -0.22 | -14.9 | <0.001 | <0.001 | 0.67 | 0.45 | -0.22 |
| cg18878432 | -0.26 | -14.9 | <0.001 | <0.001 | 0.72 | 0.46 | -0.26 |
| cg14848450 | -0.20 | -14.9 | <0.001 | <0.001 | 0.82 | 0.62 | -0.20 |
| cg24172509 | -0.27 | -14.8 | <0.001 | <0.001 | 0.63 | 0.36 | -0.27 |
| cg27028202 | -0.20 | -14.8 | <0.001 | <0.001 | 0.84 | 0.64 | -0.20 |
| cg03415545 | -0.22 | -14.8 | <0.001 | <0.001 | 0.79 | 0.58 | -0.22 |
| cg07179981 | -0.24 | -14.8 | <0.001 | <0.001 | 0.67 | 0.43 | -0.24 |
| cg01384290 | -0.22 | -14.8 | <0.001 | <0.001 | 0.75 | 0.53 | -0.22 |
| cg15174906 | -0.21 | -14.8 | <0.001 | <0.001 | 0.64 | 0.43 | -0.21 |
| cg22328208 | -0.21 | -14.8 | <0.001 | <0.001 | 0.66 | 0.45 | -0.21 |
| cg15943038 | -0.22 | -14.8 | <0.001 | <0.001 | 0.67 | 0.45 | -0.22 |
| cg07470694 | -0.24 | -14.7 | <0.001 | <0.001 | 0.83 | 0.59 | -0.24 |
| cg04014328 | -0.21 | -14.7 | <0.001 | <0.001 | 0.74 | 0.53 | -0.21 |
| cg15844419 | -0.22 | -14.7 | <0.001 | <0.001 | 0.64 | 0.42 | -0.22 |
| cg15066837 | -0.24 | -14.7 | <0.001 | <0.001 | 0.73 | 0.50 | -0.24 |
| cg19537719 | -0.28 | -14.7 | <0.001 | <0.001 | 0.61 | 0.34 | -0.28 |
| cg08977931 | -0.20 | -14.7 | <0.001 | <0.001 | 0.63 | 0.43 | -0.20 |
| cg21388639 | -0.22 | -14.7 | <0.001 | <0.001 | 0.71 | 0.49 | -0.22 |
| cg11021321 | -0.21 | -14.7 | <0.001 | <0.001 | 0.78 | 0.57 | -0.21 |
| cg26144594 | -0.22 | -14.7 | <0.001 | <0.001 | 0.65 | 0.43 | -0.22 |
| cg26064470 | -0.21 | -14.7 | <0.001 | <0.001 | 0.69 | 0.48 | -0.21 |
| cg05371552 | -0.22 | -14.7 | <0.001 | <0.001 | 0.64 | 0.42 | -0.22 |
| cg10588962 | -0.23 | -14.6 | <0.001 | <0.001 | 0.67 | 0.43 | -0.23 |
| cg23115390 | -0.28 | -14.6 | <0.001 | <0.001 | 0.68 | 0.40 | -0.28 |
| cg14108567 | -0.26 | -14.6 | <0.001 | <0.001 | 0.75 | 0.49 | -0.26 |
| cg09580336 | -0.25 | -14.6 | <0.001 | <0.001 | 0.60 | 0.35 | -0.25 |
| cg11508406 | -0.22 | -14.6 | <0.001 | <0.001 | 0.65 | 0.43 | -0.22 |
| cg02311374 | -0.20 | -14.6 | <0.001 | <0.001 | 0.70 | 0.49 | -0.20 |
| cg04063345 | -0.26 | -14.6 | <0.001 | <0.001 | 0.65 | 0.39 | -0.26 |
| cg01569664 | -0.20 | -14.6 | <0.001 | <0.001 | 0.71 | 0.51 | -0.20 |
| cg12304520 | -0.22 | -14.5 | <0.001 | <0.001 | 0.64 | 0.42 | -0.22 |
| cg01363324 | -0.21 | -14.5 | <0.001 | <0.001 | 0.60 | 0.39 | -0.21 |
| cg27287593 | -0.24 | -14.5 | <0.001 | <0.001 | 0.70 | 0.46 | -0.24 |
| cg24095120 | -0.21 | -14.5 | <0.001 | <0.001 | 0.79 | 0.58 | -0.21 |
| cg01840128 | -0.20 | -14.5 | <0.001 | <0.001 | 0.80 | 0.60 | -0.20 |
| cg07902749 | 0.21 | 14.5 | <0.001 | <0.001 | 0.16 | 0.37 | 0.21 |
| cg01341751 | -0.22 | -14.5 | <0.001 | <0.001 | 0.69 | 0.46 | -0.22 |
| cg22884714 | -0.22 | -14.5 | <0.001 | <0.001 | 0.78 | 0.56 | -0.22 |
| cg14398214 | -0.24 | -14.5 | <0.001 | <0.001 | 0.71 | 0.48 | -0.24 |
| cg15932961 | -0.20 | -14.5 | <0.001 | <0.001 | 0.67 | 0.46 | -0.20 |
| cg18378955 | -0.22 | -14.4 | <0.001 | <0.001 | 0.73 | 0.51 | -0.22 |
| cg18620306 | -0.22 | -14.4 | <0.001 | <0.001 | 0.81 | 0.60 | -0.22 |
| cg16032894 | -0.20 | -14.4 | <0.001 | <0.001 | 0.70 | 0.50 | -0.20 |
| cg12349571 | -0.22 | -14.3 | <0.001 | <0.001 | 0.83 | 0.61 | -0.22 |
| cg11900509 | -0.23 | -14.3 | <0.001 | <0.001 | 0.80 | 0.57 | -0.23 |
| cg24202131 | -0.20 | -14.3 | <0.001 | <0.001 | 0.64 | 0.44 | -0.20 |
| cg13746854 | -0.20 | -14.3 | <0.001 | <0.001 | 0.68 | 0.47 | -0.20 |
| cg24150623 | -0.23 | -14.3 | <0.001 | <0.001 | 0.70 | 0.47 | -0.23 |
| cg01936839 | -0.22 | -14.3 | <0.001 | <0.001 | 0.72 | 0.51 | -0.22 |
| cg04539515 | -0.21 | -14.3 | <0.001 | <0.001 | 0.64 | 0.43 | -0.21 |
| cg22993195 | -0.21 | -14.3 | <0.001 | <0.001 | 0.66 | 0.45 | -0.21 |
| cg02227036 | -0.23 | -14.3 | <0.001 | <0.001 | 0.77 | 0.54 | -0.23 |
| cg16266893 | -0.21 | -14.3 | <0.001 | <0.001 | 0.70 | 0.48 | -0.21 |
| cg15684563 | -0.23 | -14.3 | <0.001 | <0.001 | 0.76 | 0.53 | -0.23 |
| cg04043455 | -0.20 | -14.3 | <0.001 | <0.001 | 0.71 | 0.50 | -0.20 |
| cg01948202 | 0.24 | 14.2 | <0.001 | <0.001 | 0.19 | 0.42 | 0.24 |
| cg02124887 | 0.22 | 14.2 | <0.001 | <0.001 | 0.17 | 0.39 | 0.22 |
| cg01889169 | -0.26 | -14.2 | <0.001 | <0.001 | 0.75 | 0.49 | -0.26 |
| cg04608582 | -0.23 | -14.2 | <0.001 | <0.001 | 0.61 | 0.39 | -0.23 |
| cg16324958 | -0.22 | -14.2 | <0.001 | <0.001 | 0.61 | 0.39 | -0.22 |
| cg16409977 | -0.20 | -14.2 | <0.001 | <0.001 | 0.71 | 0.51 | -0.20 |
| cg26860604 | -0.24 | -14.2 | <0.001 | <0.001 | 0.73 | 0.49 | -0.24 |
| cg23300486 | -0.22 | -14.2 | <0.001 | <0.001 | 0.79 | 0.58 | -0.22 |
| cg08577953 | -0.21 | -14.2 | <0.001 | <0.001 | 0.65 | 0.44 | -0.21 |
| cg19839825 | -0.22 | -14.1 | <0.001 | <0.001 | 0.73 | 0.52 | -0.22 |
| cg06766016 | -0.27 | -14.1 | <0.001 | <0.001 | 0.63 | 0.36 | -0.27 |
| cg24187351 | -0.24 | -14.1 | <0.001 | <0.001 | 0.73 | 0.49 | -0.24 |
| cg00424967 | -0.21 | -14.1 | <0.001 | <0.001 | 0.73 | 0.51 | -0.21 |
| cg07124972 | -0.21 | -14.1 | <0.001 | <0.001 | 0.74 | 0.53 | -0.21 |
| cg05342782 | -0.21 | -14.1 | <0.001 | <0.001 | 0.73 | 0.52 | -0.21 |
| cg17868307 | -0.21 | -14.1 | <0.001 | <0.001 | 0.76 | 0.55 | -0.21 |
| cg13294846 | -0.20 | -14.1 | <0.001 | <0.001 | 0.71 | 0.51 | -0.20 |
| cg23401796 | -0.24 | -14.1 | <0.001 | <0.001 | 0.64 | 0.41 | -0.24 |
| cg03654727 | -0.24 | -14.1 | <0.001 | <0.001 | 0.85 | 0.61 | -0.24 |
| cg13493043 | -0.21 | -14.0 | <0.001 | <0.001 | 0.62 | 0.41 | -0.21 |
| cg10323433 | -0.22 | -14.0 | <0.001 | <0.001 | 0.67 | 0.46 | -0.22 |
| cg08560074 | -0.23 | -14.0 | <0.001 | <0.001 | 0.77 | 0.54 | -0.23 |
| cg19506623 | -0.23 | -14.0 | <0.001 | <0.001 | 0.73 | 0.50 | -0.23 |
| cg09671951 | 0.22 | 14.0 | <0.001 | <0.001 | 0.12 | 0.35 | 0.22 |
| cg17778888 | -0.23 | -14.0 | <0.001 | <0.001 | 0.92 | 0.69 | -0.23 |
| cg19592277 | -0.23 | -14.0 | <0.001 | <0.001 | 0.66 | 0.43 | -0.23 |
| cg13260976 | -0.22 | -14.0 | <0.001 | <0.001 | 0.65 | 0.43 | -0.22 |
| cg02442693 | -0.23 | -14.0 | <0.001 | <0.001 | 0.60 | 0.37 | -0.23 |
| cg10286969 | 0.20 | 13.9 | <0.001 | <0.001 | 0.18 | 0.38 | 0.20 |
| cg10824810 | -0.20 | -13.9 | <0.001 | <0.001 | 0.71 | 0.51 | -0.20 |
| cg05522011 | -0.25 | -13.8 | <0.001 | <0.001 | 0.62 | 0.37 | -0.25 |
| cg23907051 | -0.25 | -13.8 | <0.001 | <0.001 | 0.67 | 0.42 | -0.25 |
| cg16548605 | -0.22 | -13.8 | <0.001 | <0.001 | 0.75 | 0.53 | -0.22 |
| cg18454133 | -0.20 | -13.8 | <0.001 | <0.001 | 0.78 | 0.58 | -0.20 |
| cg10495931 | -0.21 | -13.8 | <0.001 | <0.001 | 0.62 | 0.41 | -0.21 |
| cg10868156 | -0.20 | -13.8 | <0.001 | <0.001 | 0.69 | 0.49 | -0.20 |
| cg16752592 | -0.24 | -13.8 | <0.001 | <0.001 | 0.69 | 0.45 | -0.24 |
| cg10605735 | -0.22 | -13.7 | <0.001 | <0.001 | 0.66 | 0.45 | -0.22 |
| cg15366127 | -0.21 | -13.7 | <0.001 | <0.001 | 0.82 | 0.60 | -0.21 |
| cg14167415 | -0.20 | -13.7 | <0.001 | <0.001 | 0.60 | 0.40 | -0.20 |
| cg10533161 | -0.24 | -13.7 | <0.001 | <0.001 | 0.86 | 0.62 | -0.24 |
| cg01961105 | -0.21 | -13.7 | <0.001 | <0.001 | 0.61 | 0.39 | -0.21 |
| cg22022580 | -0.28 | -13.6 | <0.001 | <0.001 | 0.65 | 0.37 | -0.28 |
| cg14126601 | -0.21 | -13.6 | <0.001 | <0.001 | 0.61 | 0.40 | -0.21 |
| cg16291699 | -0.21 | -13.6 | <0.001 | <0.001 | 0.76 | 0.54 | -0.21 |
| cg18173726 | -0.21 | -13.6 | <0.001 | <0.001 | 0.82 | 0.60 | -0.21 |
| cg18812904 | -0.27 | -13.6 | <0.001 | <0.001 | 0.61 | 0.34 | -0.27 |
| cg03729251 | -0.27 | -13.5 | <0.001 | <0.001 | 0.61 | 0.34 | -0.27 |
| cg16579981 | -0.20 | -13.5 | <0.001 | <0.001 | 0.64 | 0.44 | -0.20 |
| cg11214001 | -0.20 | -13.5 | <0.001 | <0.001 | 0.74 | 0.53 | -0.20 |
| cg17228232 | -0.24 | -13.5 | <0.001 | <0.001 | 0.69 | 0.44 | -0.24 |
| cg03625662 | -0.26 | -13.5 | <0.001 | <0.001 | 0.79 | 0.53 | -0.26 |
| cg23245720 | -0.20 | -13.5 | <0.001 | <0.001 | 0.90 | 0.70 | -0.20 |
| cg27573308 | -0.22 | -13.5 | <0.001 | <0.001 | 0.83 | 0.61 | -0.22 |
| cg07687354 | -0.22 | -13.5 | <0.001 | <0.001 | 0.68 | 0.46 | -0.22 |
| cg02526981 | -0.22 | -13.5 | <0.001 | <0.001 | 0.80 | 0.58 | -0.22 |
| cg08006672 | -0.22 | -13.5 | <0.001 | <0.001 | 0.81 | 0.59 | -0.22 |
| cg25196508 | -0.22 | -13.5 | <0.001 | <0.001 | 0.67 | 0.45 | -0.22 |
| cg14701597 | -0.23 | -13.5 | <0.001 | <0.001 | 0.71 | 0.48 | -0.23 |
| cg07613945 | -0.20 | -13.4 | <0.001 | <0.001 | 0.66 | 0.46 | -0.20 |
| cg14972139 | -0.22 | -13.4 | <0.001 | <0.001 | 0.69 | 0.47 | -0.22 |
| cg02484127 | -0.21 | -13.4 | <0.001 | <0.001 | 0.71 | 0.49 | -0.21 |
| cg19511748 | -0.21 | -13.4 | <0.001 | <0.001 | 0.64 | 0.44 | -0.21 |
| cg11085762 | -0.21 | -13.4 | <0.001 | <0.001 | 0.74 | 0.53 | -0.21 |
| cg25809561 | -0.27 | -13.4 | <0.001 | <0.001 | 0.61 | 0.35 | -0.27 |
| cg19486070 | -0.31 | -13.3 | <0.001 | <0.001 | 0.69 | 0.39 | -0.31 |
| cg13406860 | -0.23 | -13.3 | <0.001 | <0.001 | 0.76 | 0.53 | -0.23 |
| cg04567302 | -0.21 | -13.3 | <0.001 | <0.001 | 0.67 | 0.46 | -0.21 |
| cg18232861 | -0.20 | -13.3 | <0.001 | <0.001 | 0.61 | 0.41 | -0.20 |
| cg22369786 | -0.24 | -13.3 | <0.001 | <0.001 | 0.67 | 0.43 | -0.24 |
| cg23166590 | -0.21 | -13.3 | <0.001 | <0.001 | 0.64 | 0.43 | -0.21 |
| cg19810715 | -0.21 | -13.3 | <0.001 | <0.001 | 0.73 | 0.51 | -0.21 |
| cg26348487 | 0.22 | 13.3 | <0.001 | <0.001 | 0.17 | 0.38 | 0.22 |
| cg23863670 | -0.21 | -13.3 | <0.001 | <0.001 | 0.67 | 0.47 | -0.21 |
| cg24830730 | -0.29 | -13.2 | <0.001 | <0.001 | 0.64 | 0.36 | -0.29 |
| cg14358088 | -0.20 | -13.2 | <0.001 | <0.001 | 0.70 | 0.50 | -0.20 |
| cg14517355 | -0.21 | -13.2 | <0.001 | <0.001 | 0.64 | 0.43 | -0.21 |
| cg08610426 | -0.23 | -13.2 | <0.001 | <0.001 | 0.75 | 0.52 | -0.23 |
| cg19986012 | -0.21 | -13.2 | <0.001 | <0.001 | 0.73 | 0.51 | -0.21 |
| cg20761810 | -0.22 | -13.2 | <0.001 | <0.001 | 0.73 | 0.51 | -0.22 |
| cg07591442 | -0.22 | -13.2 | <0.001 | <0.001 | 0.74 | 0.52 | -0.22 |
| cg19113686 | -0.22 | -13.2 | <0.001 | <0.001 | 0.82 | 0.60 | -0.22 |
| cg12456825 | -0.21 | -13.2 | <0.001 | <0.001 | 0.68 | 0.47 | -0.21 |
| cg22605290 | -0.24 | -13.2 | <0.001 | <0.001 | 0.65 | 0.41 | -0.24 |
| cg16695576 | -0.22 | -13.1 | <0.001 | <0.001 | 0.62 | 0.41 | -0.22 |
| cg22291711 | -0.27 | -13.1 | <0.001 | <0.001 | 0.67 | 0.40 | -0.27 |
| cg07970752 | -0.26 | -13.1 | <0.001 | <0.001 | 0.65 | 0.39 | -0.26 |
| cg00731304 | -0.21 | -13.1 | <0.001 | <0.001 | 0.83 | 0.62 | -0.21 |
| cg10946573 | -0.20 | -13.1 | <0.001 | <0.001 | 0.69 | 0.49 | -0.20 |
| cg14574910 | -0.24 | -13.0 | <0.001 | <0.001 | 0.73 | 0.49 | -0.24 |
| cg03548062 | -0.24 | -13.0 | <0.001 | <0.001 | 0.69 | 0.45 | -0.24 |
| cg23336695 | -0.21 | -13.0 | <0.001 | <0.001 | 0.61 | 0.40 | -0.21 |
| cg20703790 | -0.20 | -13.0 | <0.001 | <0.001 | 0.74 | 0.54 | -0.20 |
| cg11784799 | -0.23 | -13.0 | <0.001 | <0.001 | 0.62 | 0.40 | -0.23 |
| cg24015747 | -0.20 | -12.9 | <0.001 | <0.001 | 0.66 | 0.46 | -0.20 |
| cg11552829 | -0.24 | -12.8 | <0.001 | <0.001 | 0.81 | 0.56 | -0.24 |
| cg16201883 | -0.20 | -12.8 | <0.001 | <0.001 | 0.73 | 0.53 | -0.20 |
| cg21270847 | -0.27 | -12.8 | <0.001 | <0.001 | 0.76 | 0.48 | -0.27 |
| cg27185423 | -0.20 | -12.7 | <0.001 | <0.001 | 0.75 | 0.54 | -0.20 |
| cg15326452 | -0.21 | -12.6 | <0.001 | <0.001 | 0.74 | 0.53 | -0.21 |
| cg16287284 | -0.21 | -12.6 | <0.001 | <0.001 | 0.69 | 0.49 | -0.21 |
| cg07783843 | -0.24 | -12.5 | <0.001 | <0.001 | 0.65 | 0.42 | -0.24 |
| cg12452800 | -0.20 | -12.5 | <0.001 | <0.001 | 0.66 | 0.45 | -0.20 |
| cg18473117 | -0.23 | -12.5 | <0.001 | <0.001 | 0.67 | 0.44 | -0.23 |
| cg13471599 | -0.22 | -12.4 | <0.001 | <0.001 | 0.61 | 0.39 | -0.22 |
| cg09307985 | -0.21 | -12.4 | <0.001 | <0.001 | 0.64 | 0.44 | -0.21 |
| cg23657179 | -0.21 | -12.4 | <0.001 | <0.001 | 0.61 | 0.41 | -0.21 |
| cg00730077 | -0.21 | -12.3 | <0.001 | <0.001 | 0.69 | 0.48 | -0.21 |
| cg18560551 | -0.20 | -12.3 | <0.001 | <0.001 | 0.69 | 0.49 | -0.20 |
| cg07500347 | -0.21 | -12.3 | <0.001 | <0.001 | 0.73 | 0.51 | -0.21 |
| cg23191354 | -0.20 | -12.2 | <0.001 | <0.001 | 0.66 | 0.46 | -0.20 |
| cg08326019 | -0.21 | -12.2 | <0.001 | <0.001 | 0.78 | 0.56 | -0.21 |
| cg11669516 | -0.22 | -12.1 | <0.001 | <0.001 | 0.68 | 0.46 | -0.22 |
| cg16296438 | -0.21 | -12.1 | <0.001 | <0.001 | 0.72 | 0.51 | -0.21 |
| cg00804179 | -0.21 | -12.1 | <0.001 | <0.001 | 0.81 | 0.61 | -0.21 |
| cg21941030 | -0.20 | -12.1 | <0.001 | <0.001 | 0.62 | 0.42 | -0.20 |
| cg02837536 | -0.22 | -12.0 | <0.001 | <0.001 | 0.65 | 0.43 | -0.22 |
| cg11843238 | -0.22 | -11.8 | <0.001 | <0.001 | 0.62 | 0.40 | -0.22 |
| cg20107996 | -0.20 | -11.8 | <0.001 | <0.001 | 0.79 | 0.59 | -0.20 |
| cg08771171 | -0.20 | -11.8 | <0.001 | <0.001 | 0.63 | 0.43 | -0.20 |
| cg16139202 | -0.20 | -11.7 | <0.001 | <0.001 | 0.65 | 0.45 | -0.20 |
| cg07601068 | -0.20 | -11.7 | <0.001 | <0.001 | 0.68 | 0.47 | -0.20 |
| cg23331981 | -0.21 | -11.6 | <0.001 | <0.001 | 0.70 | 0.49 | -0.21 |
| cg05721877 | -0.24 | -11.5 | <0.001 | <0.001 | 0.66 | 0.42 | -0.24 |
| cg13593391 | -0.25 | -11.5 | <0.001 | <0.001 | 0.63 | 0.39 | -0.25 |
| cg26386273 | -0.22 | -11.0 | <0.001 | <0.001 | 0.74 | 0.52 | -0.22 |
| cg16612423 | -0.21 | -10.4 | <0.001 | <0.001 | 0.63 | 0.42 | -0.21 |
| cg08398691 | -0.22 | -10.3 | <0.001 | <0.001 | 0.73 | 0.51 | -0.22 |
| cg18966688 | -0.21 | -8.4 | <0.001 | <0.001 | 0.65 | 0.44 | -0.21 |

**Supplementary Table S2.** ssGSEA analysis of the epigenetic signature.

| **Term** | **logFC** | **t** | **P.Value** | **adj.P.Val** |
| --- | --- | --- | --- | --- |
| PRIMARY_IMMUNODEFICIENCY | 0.04 | 5.63 | 3.96E-08 | 3.68E-07 |
| ALPHA_LINOLENIC_ACID_METABOLISM | 0.02 | 6.43 | 4.82E-10 | 1.28E-08 |
| GLYCOSAMINOGLYCAN_BIOSYNTHESIS_KERATAN_SULFATE | 0.02 | 5.54 | 6.61E-08 | 5.86E-07 |
| SULFUR_METABOLISM | 0.02 | 4.56 | 7.41E-06 | 3.94E-05 |
| CYTOSOLIC_DNA_SENSING_PATHWAY | 0.02 | 6.21 | 1.66E-09 | 3.42E-08 |
| GLYCOSAMINOGLYCAN_BIOSYNTHESIS_CHONDROITIN_SULFATE | 0.02 | 4.71 | 3.79E-06 | 2.14E-05 |
| CYTOKINE_CYTOKINE_RECEPTOR_INTERACTION | 0.02 | 5.32 | 2.02E-07 | 1.40E-06 |
| P53_SIGNALING_PATHWAY | 0.02 | 7.45 | 9.13E-13 | 5.66E-11 |
| HEMATOPOIETIC_CELL_LINEAGE | 0.02 | 3.95 | 9.50E-05 | 0.000347 |
| NOD_LIKE_RECEPTOR_SIGNALING_PATHWAY | 0.02 | 6.18 | 2.02E-09 | 3.69E-08 |
| ONE_CARBON_POOL_BY_FOLATE | -0.02 | -4.08 | 5.62E-05 | 0.000218 |
| CIRCADIAN_RHYTHM_MAMMAL | -0.02 | -4.51 | 9.05E-06 | 4.68E-05 |
| GLYCOLYSIS_GLUCONEOGENESIS | -0.02 | -5.74 | 2.20E-08 | 2.15E-07 |
| ASCORBATE_AND_ALDARATE_METABOLISM | -0.02 | -2.66 | 0.00825 | 0.01805 |
| CITRATE_CYCLE_TCA_CYCLE | -0.02 | -5.47 | 9.42E-08 | 7.96E-07 |
| HISTIDINE_METABOLISM | -0.02 | -4.14 | 4.51E-05 | 0.000182 |
| BETA_ALANINE_METABOLISM | -0.02 | -4.80 | 2.41E-06 | 1.40E-05 |
| PPAR_SIGNALING_PATHWAY | -0.02 | -5.05 | 7.38E-07 | 4.90E-06 |
| TERPENOID_BACKBONE_BIOSYNTHESIS | -0.02 | -5.36 | 1.61E-07 | 1.25E-06 |
| LIMONENE_AND_PINENE_DEGRADATION | -0.02 | -5.81 | 1.52E-08 | 1.66E-07 |
| BUTANOATE_METABOLISM | -0.02 | -4.37 | 1.67E-05 | 7.75E-05 |
| FATTY_ACID_METABOLISM | -0.03 | -6.38 | 6.59E-10 | 1.53E-08 |
| PROPANOATE_METABOLISM | -0.03 | -6.54 | 2.55E-10 | 7.89E-09 |
| VALINE_LEUCINE_AND_ISOLEUCINE_DEGRADATION | -0.03 | -6.17 | 2.18E-09 | 3.69E-08 |
| PROXIMAL_TUBULE_BICARBONATE_RECLAMATION | -0.03 | -6.79 | 5.67E-11 | 2.11E-09 |
| RENIN_ANGIOTENSIN_SYSTEM | -0.04 | -5.98 | 5.96E-09 | 8.53E-08 |

**Supplementary Information**

**Related file 1. Ethics Committee Approval (number: 2020102).**

**
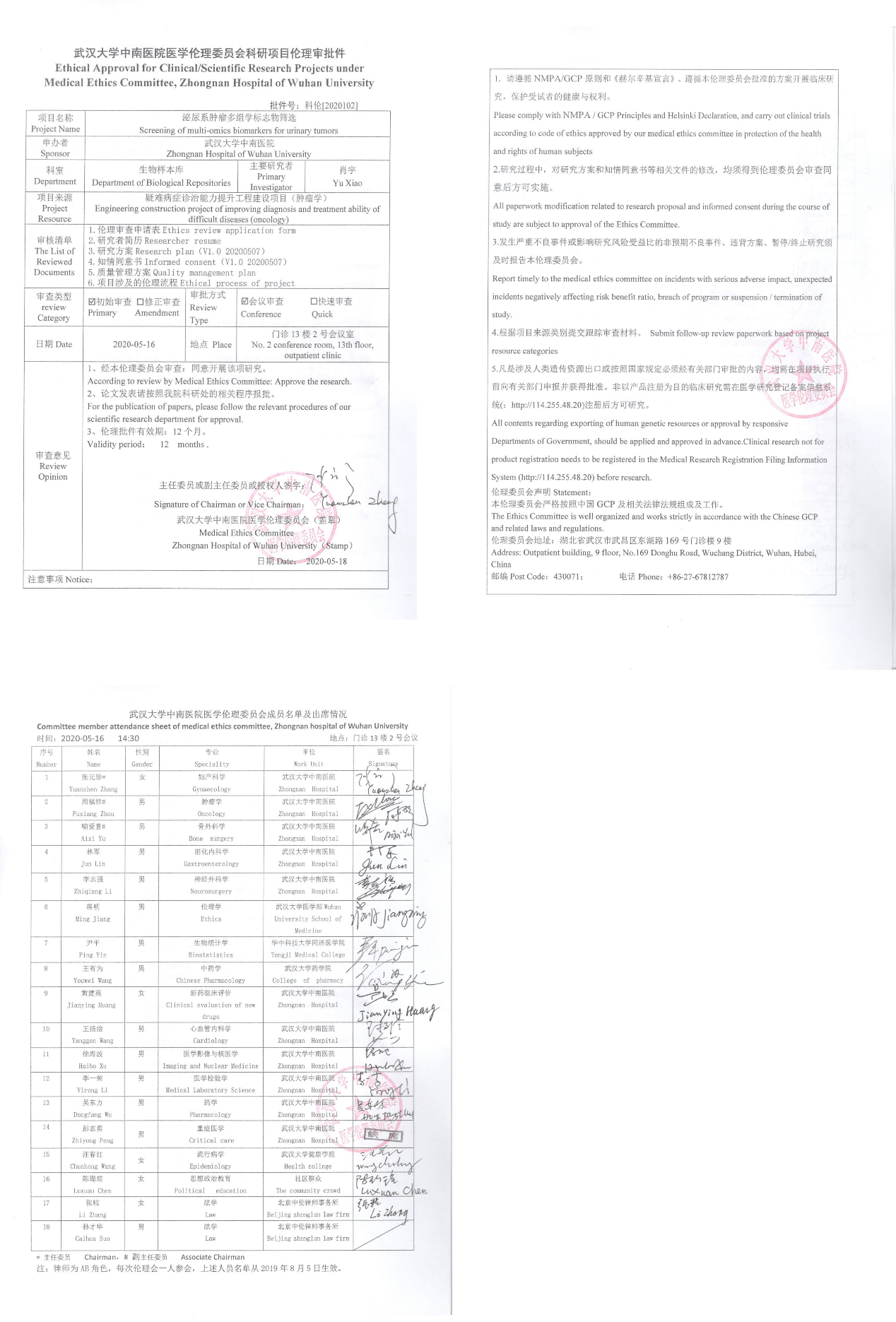
**
