# Supplementary figures and images for: Epigenetic signature predicts overall survival clear cell renal cell carcinoma
Source: Cancer Cell Int. 2020 Nov 23;20:564. doi: 10.1186/s12935-020-01640-x (PMC7686748; doi:10.1186/s12935-020-01640-x)

# A

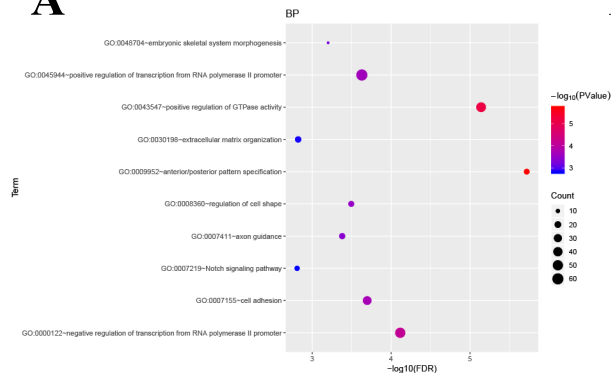

# B

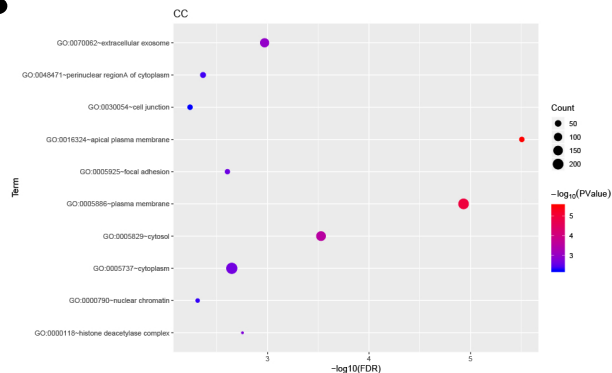

# C

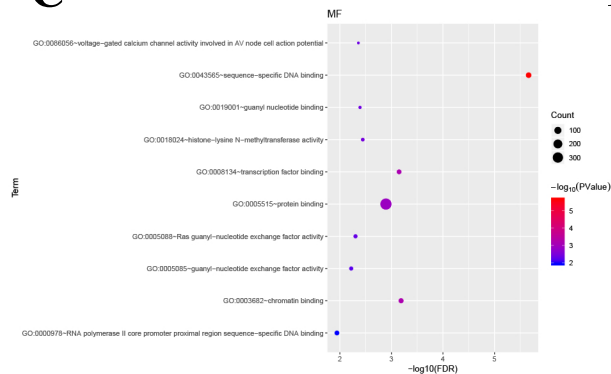

# D

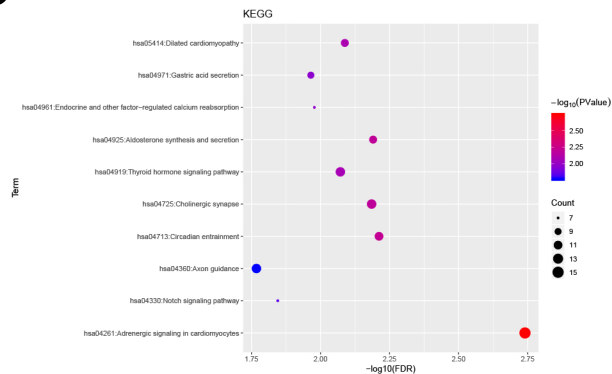

Supplement: Supplementary file 2 — Additional file 2. Supplementary figure S1: Functional annotation of the epigenetic signature. [file 12935_2020_1640_MOESM2_ESM.pdf]

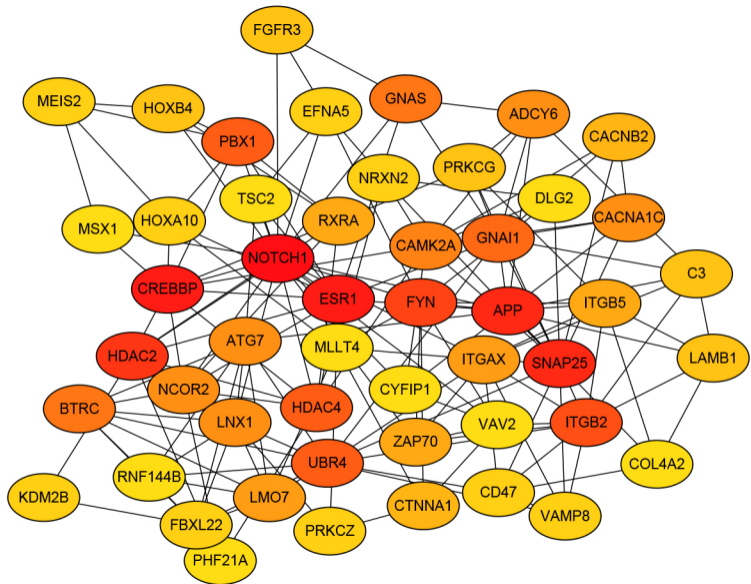

Supplement: Supplementary file 3 — Additional file 3. Supplementary figure S2: The PPI network diagram. [file 12935_2020_1640_MOESM3_ESM.pdf]
